# Supplementary material for: Genetic Alphabet Expansion Provides Versatile Specificities and Activities of Unnatural-Base DNA Aptamers Targeting Cancer Cells
Source: Mol Ther Nucleic Acids. 2018 Nov 29;14:158–70. doi: 10.1016/j.omtn.2018.11.011 (PMC6307347; doi:10.1016/j.omtn.2018.11.011)
Supplement: Document S1. Figures S1–S27 and Tables S1–S5 [file mmc1.pdf]

OMTN, Volume *14*

Supplemental Information

Genetic Alphabet Expansion Provides Versatile Specificities and Activities of Unnatural-Base DNA Aptamers Targeting Cancer Cells

Kazunobu Futami, Michiko Kimoto, Yun Wei Shermane Lim, and Ichiro Hirao

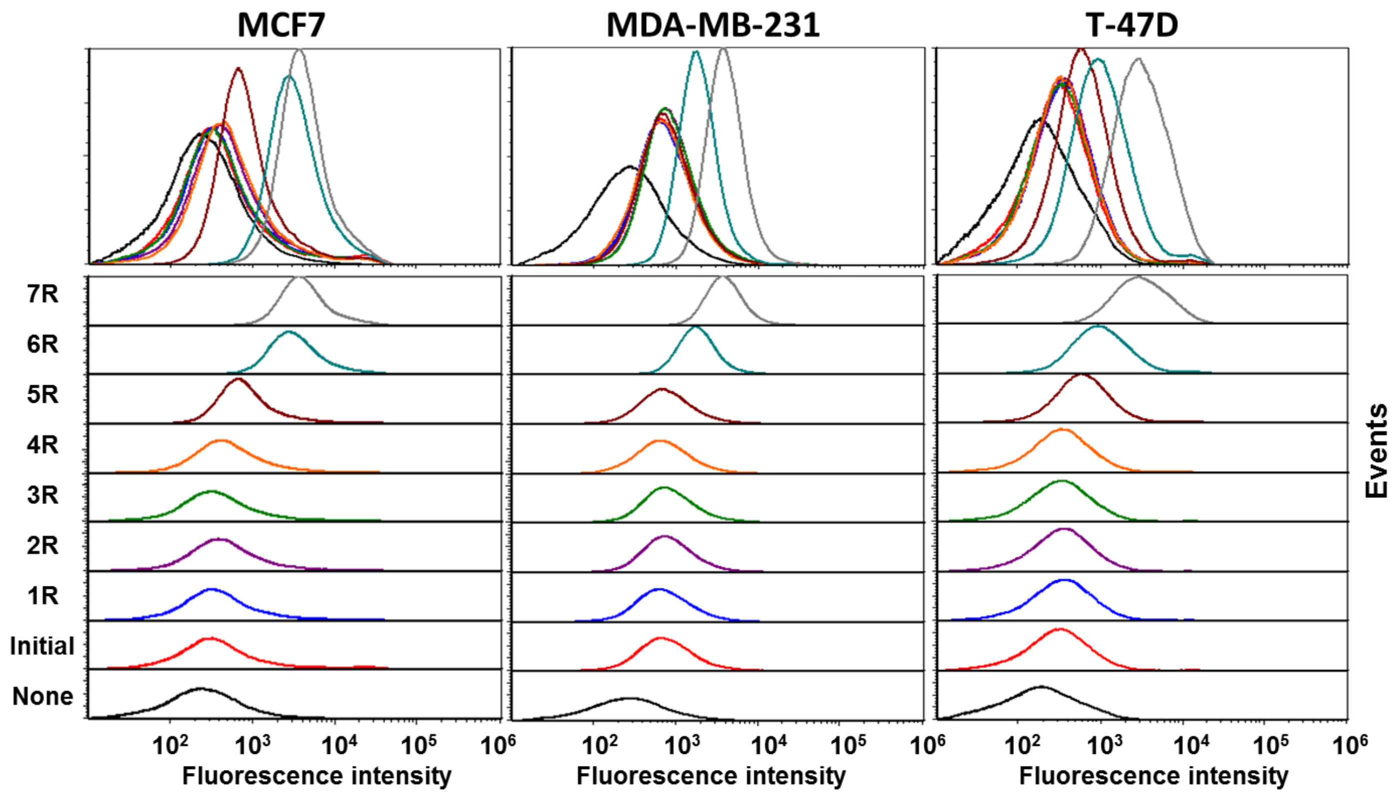

Figure S1: Monitoring the binding ability of the libraries after each cell-ExSELEX round with flow cytometry. Alexa488-labeled libraries (250 nM) were incubated with target cells at 4ºC for 30 min, washed twice, and subjected to flow cytometry analyses.

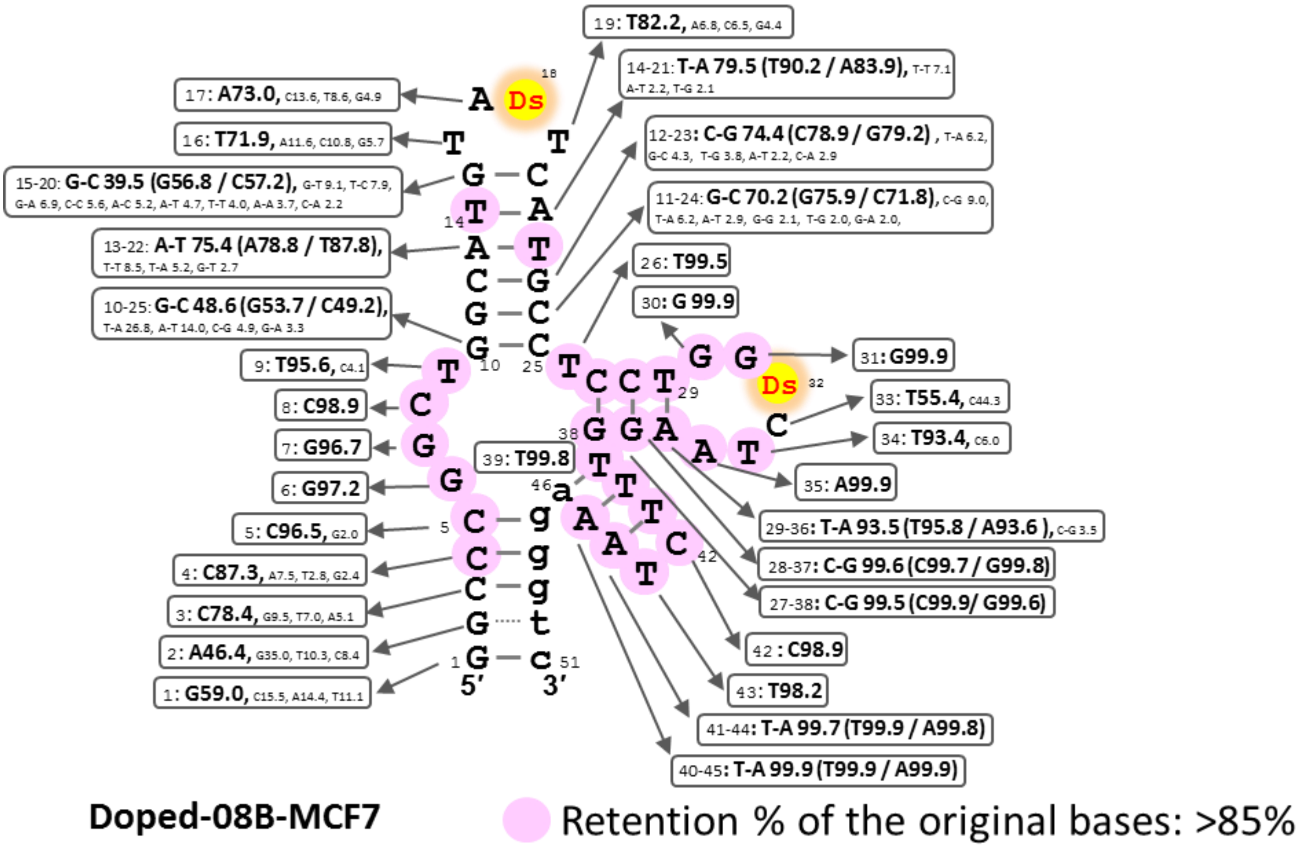

Figure S2: Predicted secondary structure of 08B-MCF7, based on highly retained nucleobases and co-varied nucleobases in sequences obtained in the doped second selection targeting MCF7. Pink circles indicate the bases with more than 85% retention after the doped selection. Small letters belong to the 3′-primer region. The ratios (more than 2%) of base pairs or bases in the putative stem or loop regions are summarized in the enclosed boxes. The base pairs or bases with the highest ratio are shown in bold.

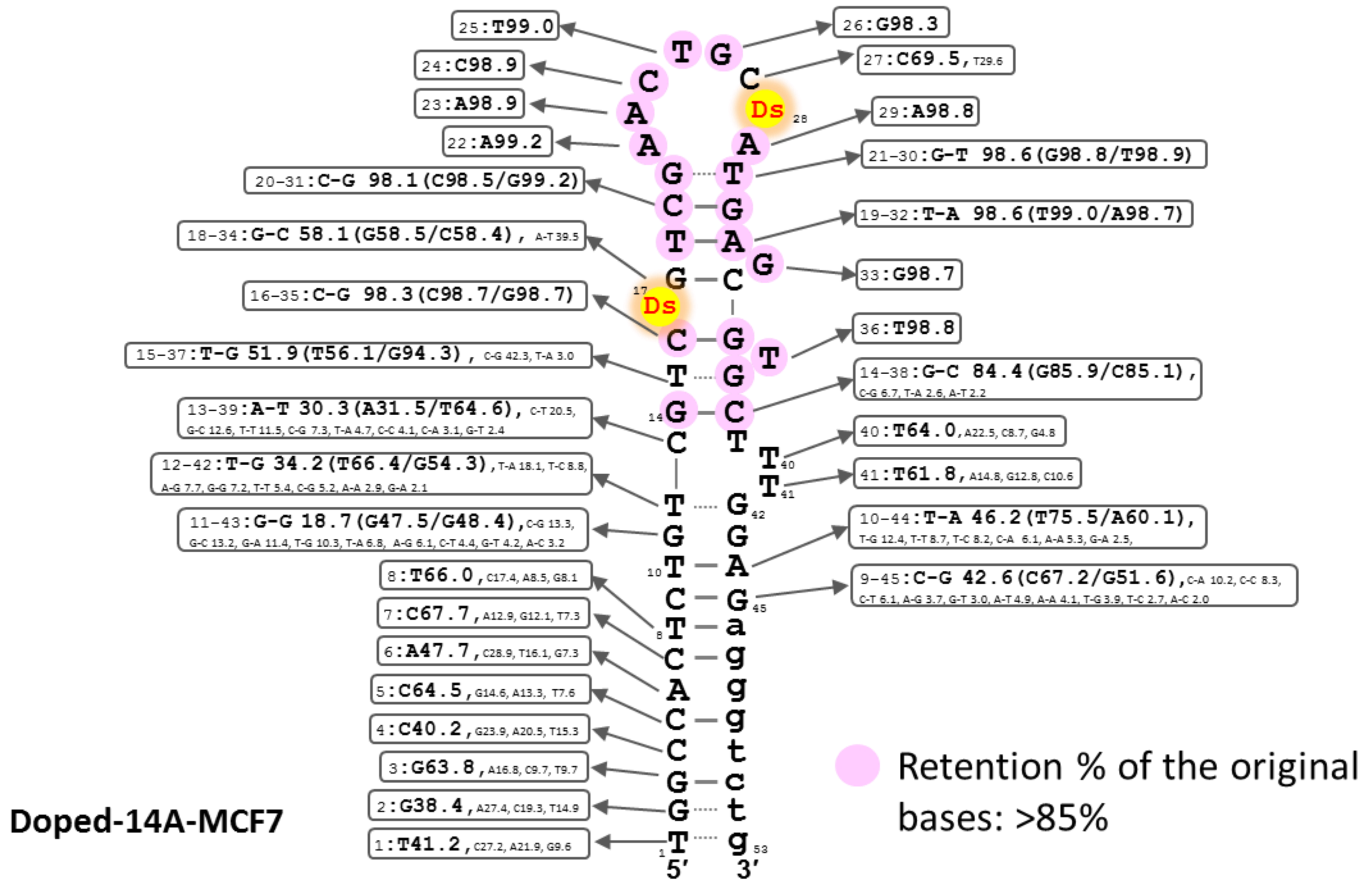

Figure S3: Predicted secondary structure of 14A-MCF7, based on highly retained nucleobases and co-varied nucleobases in sequences obtained in the doped second selection targeting MCF7. Pink circles indicate the bases with more than 85% retention after the doped selection. Small letters belong to the 3′-primer region. The ratios (more than 2%) of base pairs or bases in the putative stem or loop regions are summarized in the enclosed boxes. The base pairs or bases with the highest ratio are shown in bold.

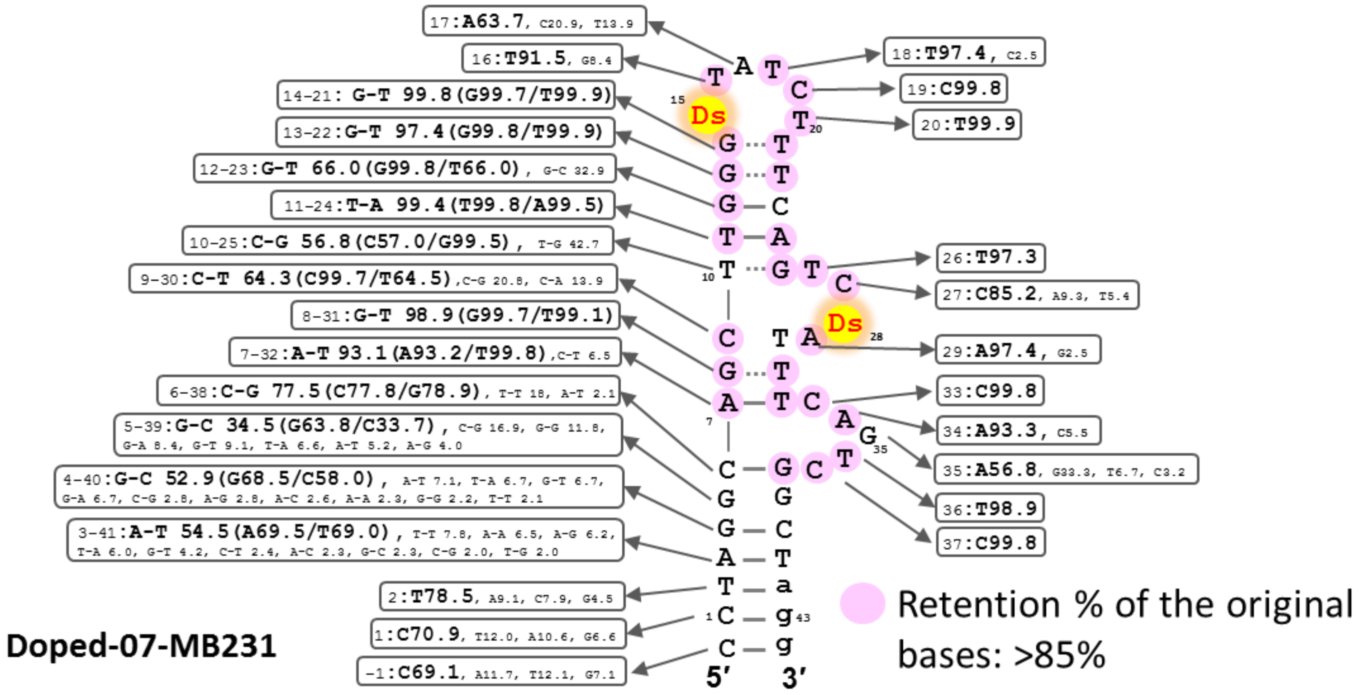

Figure S4: Predicted secondary structure of 07-MB231, based on highly retained nucleobases and co-varied nucleobases in sequences obtained in the doped second selection targeting MDA-MB-231. Pink circles indicate the bases with more than 85% retention after the doped selection. Small letters belong to the 3′-primer region. The ratios (more than 2%) of base pairs or bases in the putative stem or loop regions are summarized in the enclosed boxes. The base pairs or bases with the highest ratio are shown in bold.

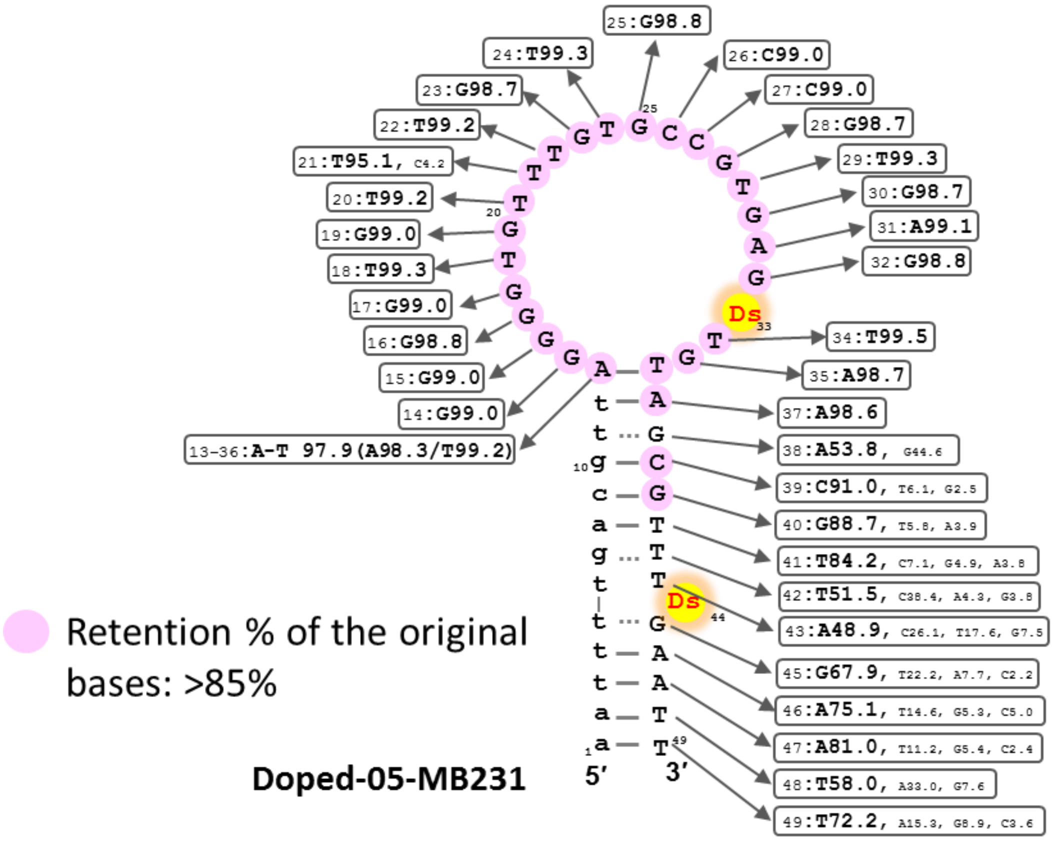

Figure S5: Predicted secondary structure of 05-MB231, based on highly retained nucleobases and co-varied nucleobases in sequences obtained in the doped second selection targeting MDA-MB-231. Pink circles indicate the bases with more than 85% retention after the doped selection. Small letters belong to the 5′-primer region. The ratios (more than 2%) of base pairs or bases in the putative stem or loop regions are summarized in the enclosed boxes. The base pairs or bases with the highest ratio are shown in bold.

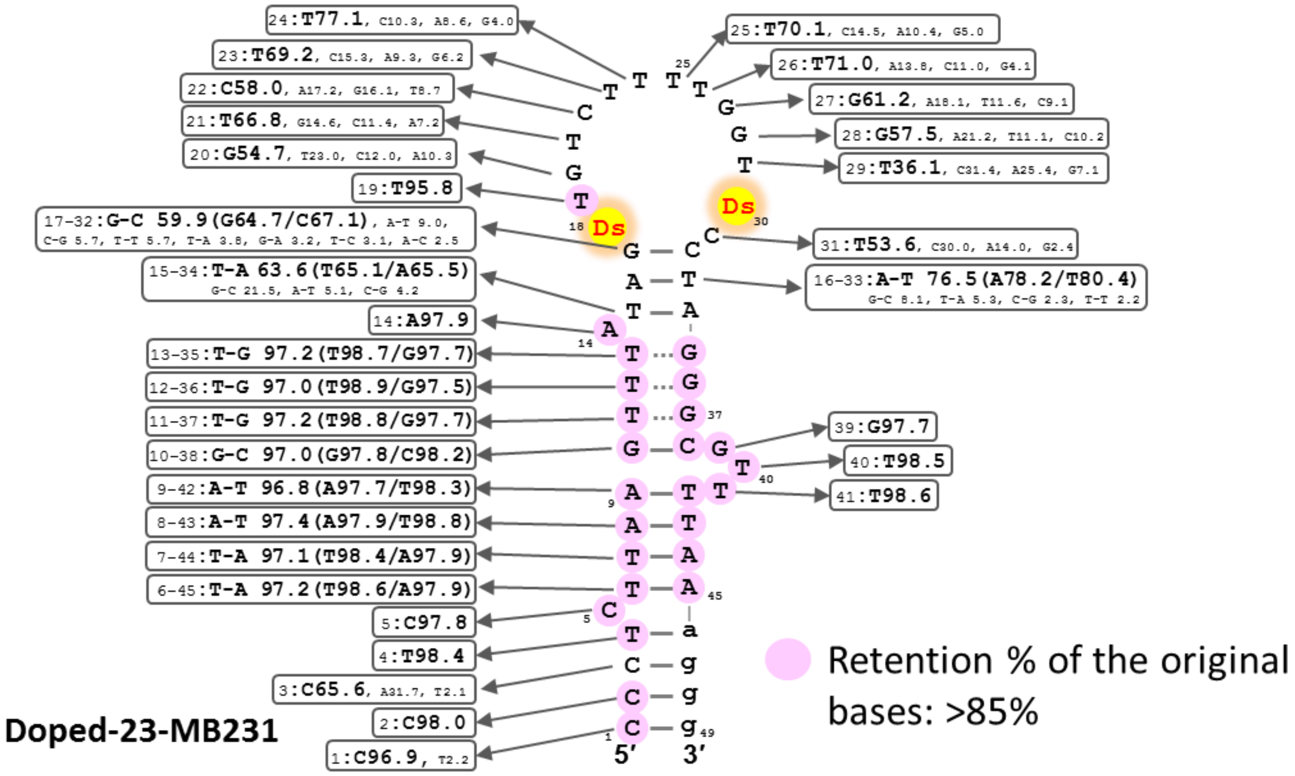

Figure S6: Predicted secondary structure of 23-MB231, based on highly retained nucleobases and co-varied nucleobases in sequences obtained in the doped second selection targeting MDA-MB-231. Pink circles indicate the bases with more than 85% retention after the selection. Small letters belong to the 3′-primer region. The ratios (more than 2%) of base pairs or bases in the putative stem or loop regions are summarized in the enclosed boxes. The base pairs or bases with the highest ratio are shown in bold.

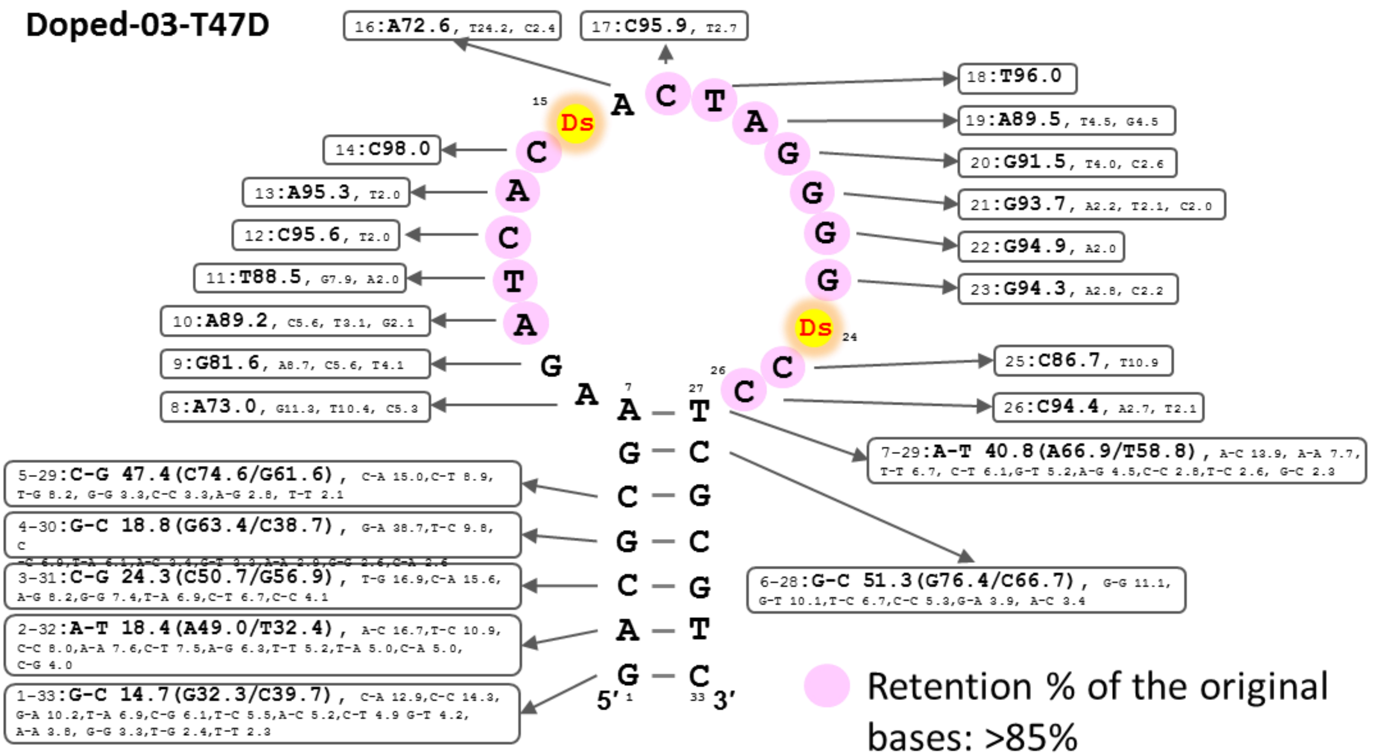

Figure S7: Predicted secondary structure of 03-T47D, based on highly retained nucleobases and co-varied nucleobases in sequences obtained in the doped second selection targeting T-47D. Pink circles indicate the bases with more than 85% retention after the doped selection. The ratios (more than 2%) of base pairs or bases in the putative stem or loop regions are summarized in the enclosed boxes. The base pairs or bases with the highest ratio are shown in bold.

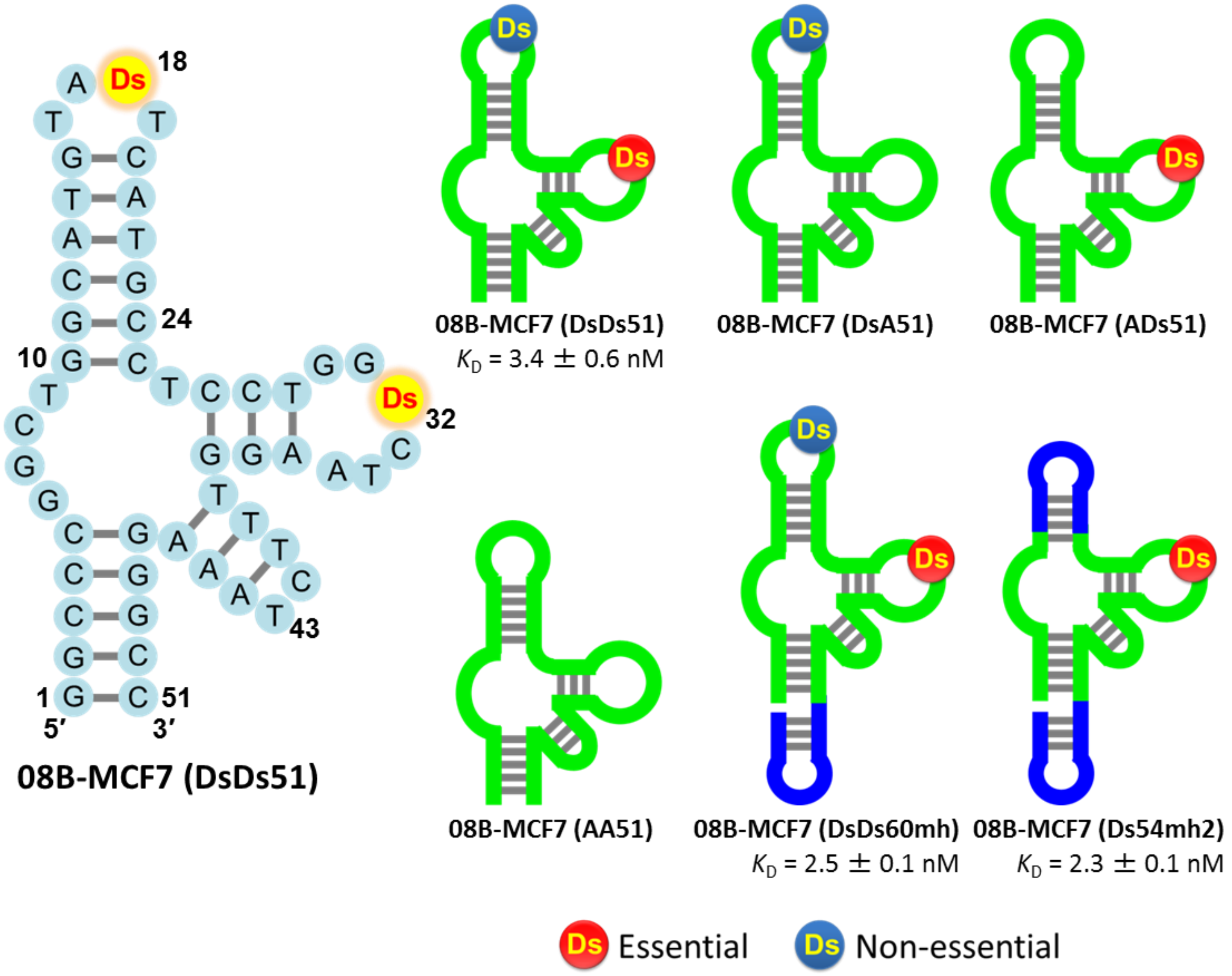

Figure S8: Schematic illustration of the secondary structures of 08B-MCF7 and its variants. The sequence and estimated secondary structures of 08B-MCF7(DsDs51) are shown on the left, and each variant is schematically represented on the right, along with its dissociation constant (K_D) to the target cell MCF7. The regions modified with a mini-hairpin are indicated by blue lines. In the DsA51, ADs51, and AA51 variants, the corresponding Ds base is replaced with A. The importance of the Ds bases for the binding was judged using the flow cytometry analysis results (see Figure S14).

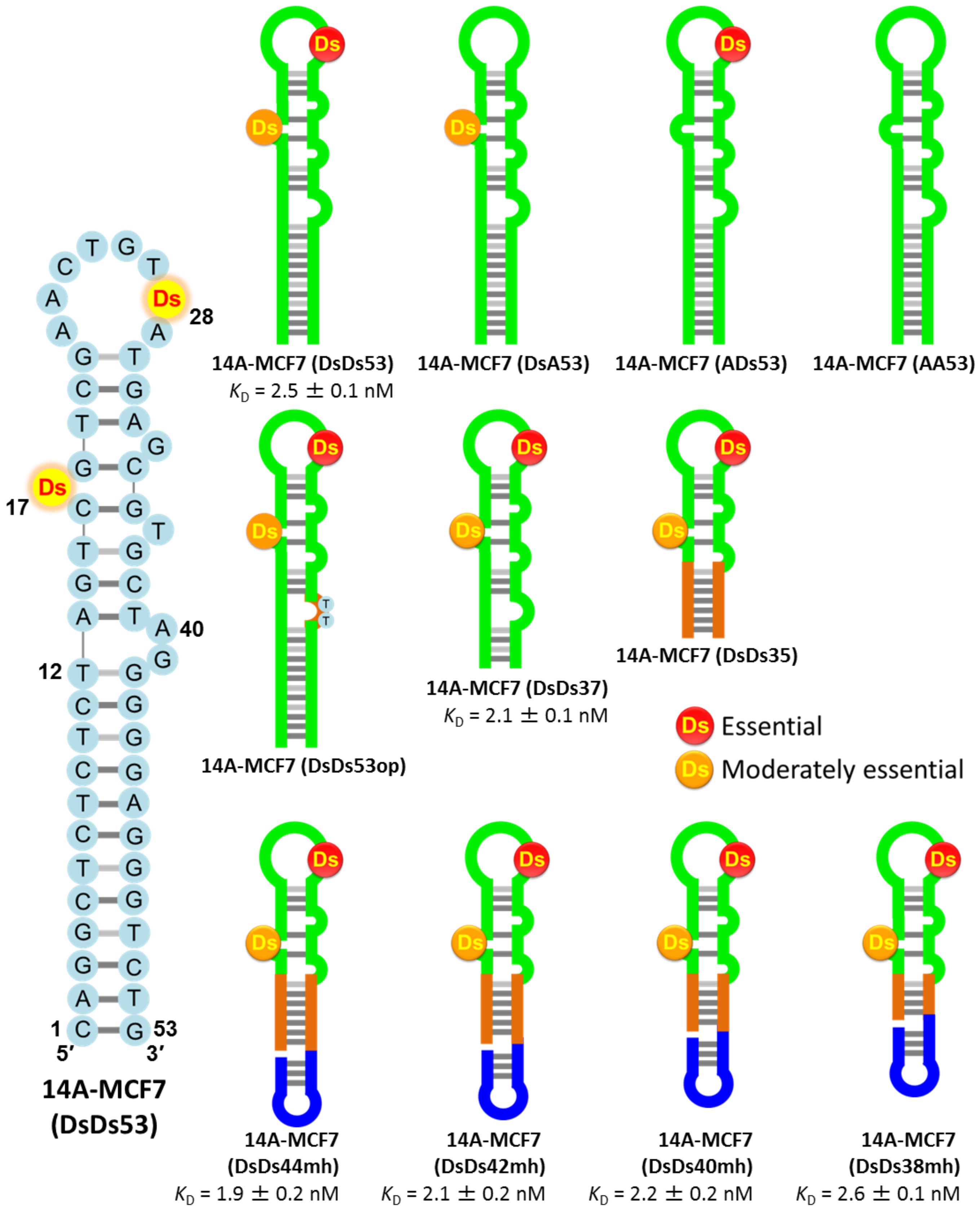

Figure S9: Schematic illustration of the secondary structures of 14A-MCF7 and its variants. The sequence and estimated secondary structures of 14A-MCF7(DsDs53) are shown on the left, and each variant is schematically represented on the right, along with its dissociation constant (K_D) to the target cell MCF7. The regions modified with a mini-hairpin DNA and G–C stem (A–T replaced with G–C) are indicated by blue and brown lines, respectively. The G–T pairing is indicated with light grey lines. In the DsA53, ADs53, and AA53 variants, the corresponding Ds base is replaced with A. The importance of the Ds bases for the binding was judged using the flow cytometry analysis results (see Figure S14).

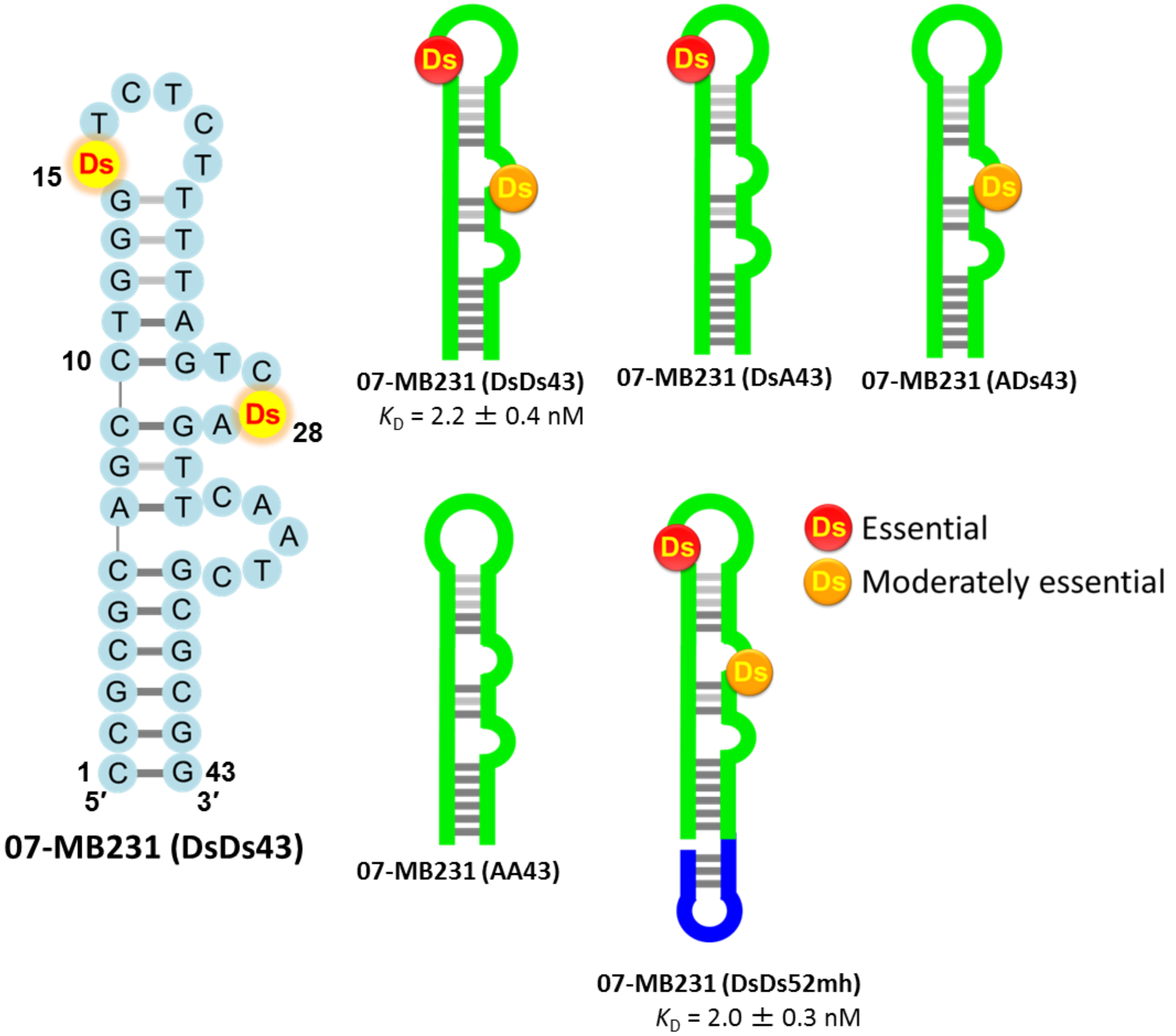

Figure S10: Schematic illustration of the secondary structures of 07-MB231 and its variants. The sequence and estimated secondary structures of 07-MB231(DsDs43) are shown on the left, and each variant is schematically represented on the right, along with its dissociation constant (K_D) to the target cell MDA-MB-231. The region modified with a mini-hairpin DNA is indicated in blue. The G–T pairing is indicated with light grey lines. In the DsA43, ADs43, and AA43 variants, the corresponding Ds base is replaced with A. The importance of the Ds bases for the binding was judged using the flow cytometry analysis results (see Figure S14).

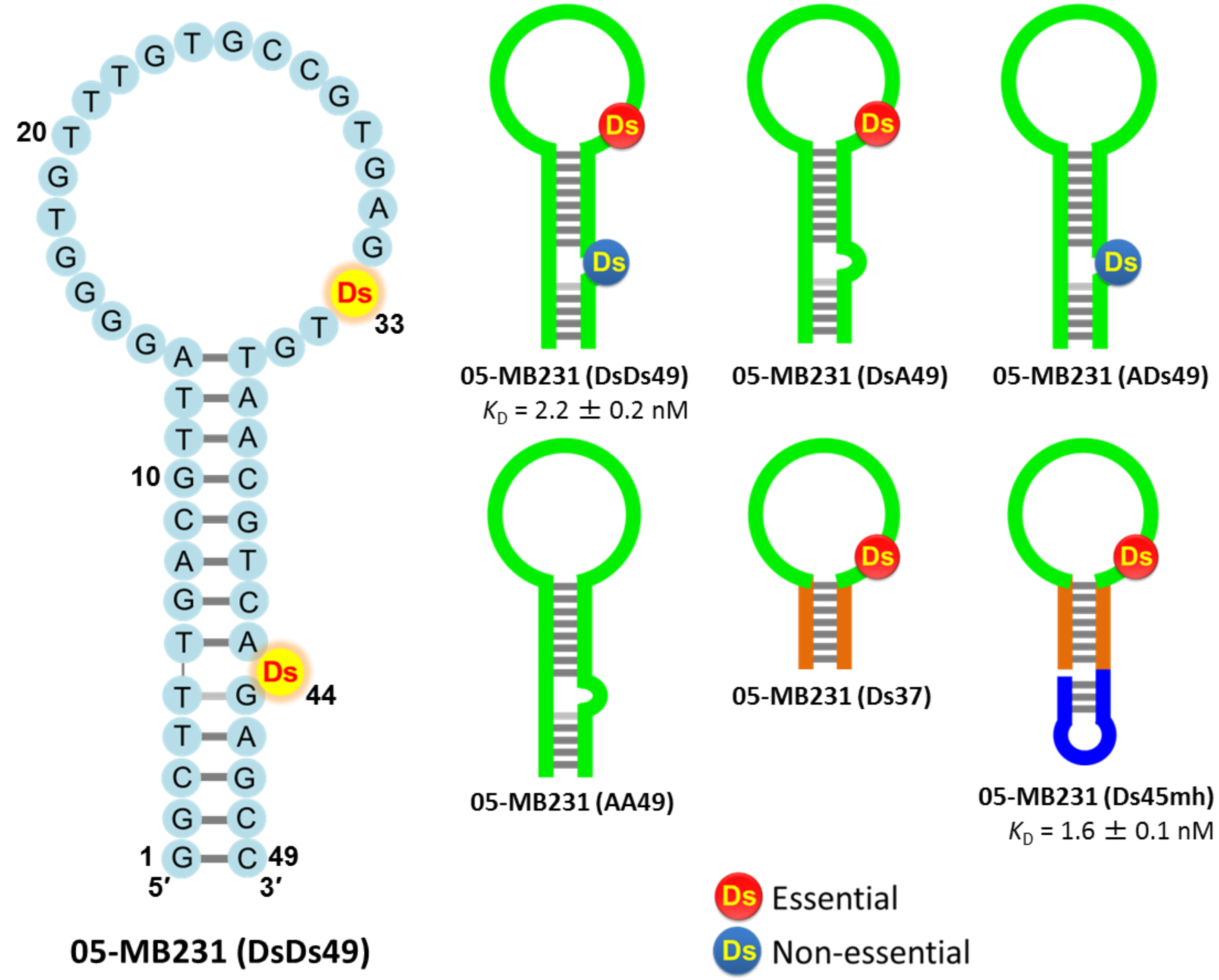

Figure S11: Schematic illustration of the secondary structures of 05-MB231 and its variants. The sequence and estimated secondary structures of 05-MB231(DsDs49) are shown on the left, and each variant is schematically represented on the right, along with its dissociation constant (K_D) to the target cell MDA-MB-231. The regions modified with a mini-hairpin DNA and a G–C stem (A–T replaced with G–C) are indicated by blue and brown lines, respectively. The G–T pairing is indicated with light grey lines. In the DsA49, ADs49, and AA49 variants, the corresponding Ds base is replaced with A. The importance of the Ds bases for the binding was judged using the flow cytometry analysis results (see Figure S14).

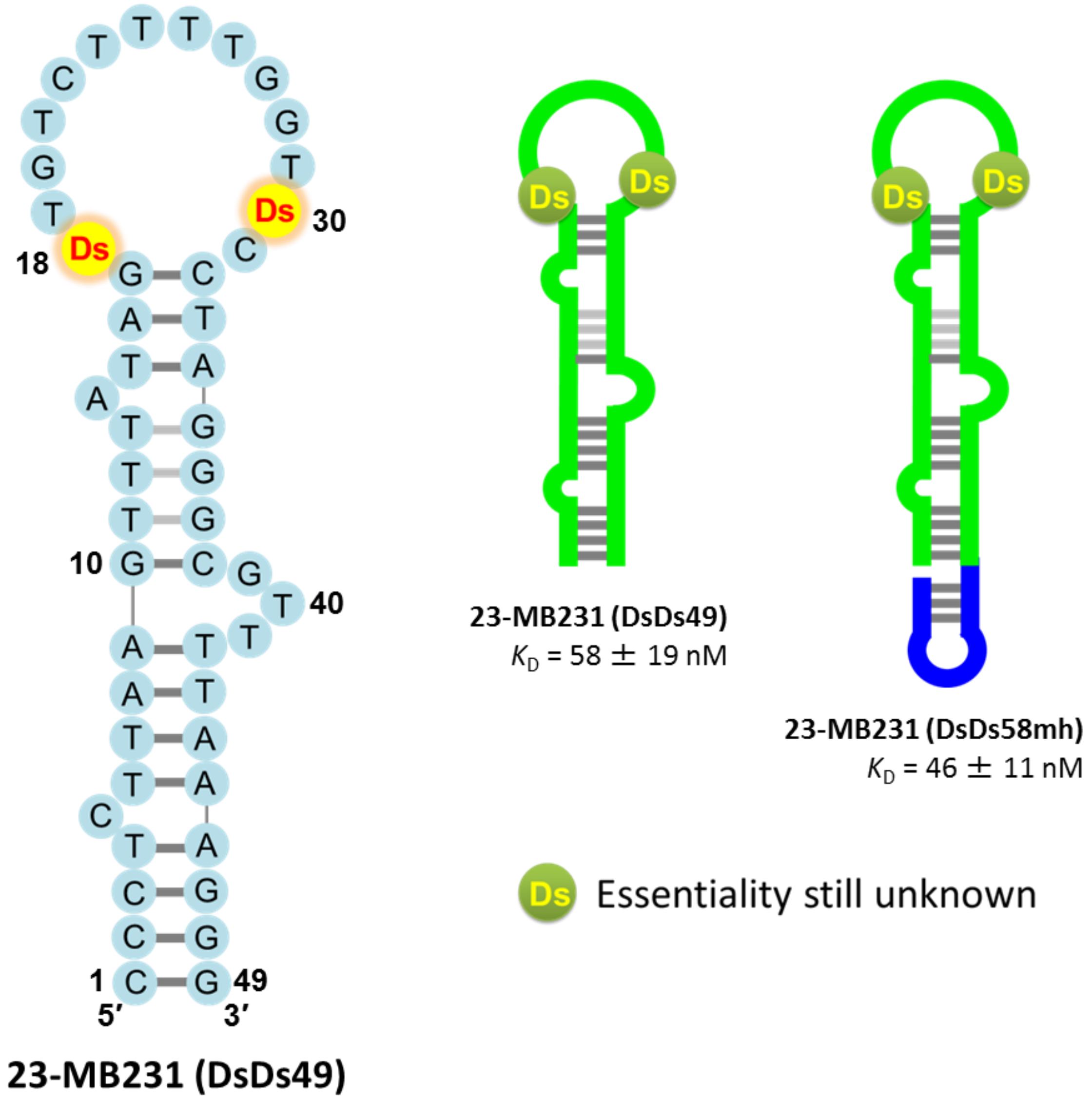

Figure S12: Schematic illustration of the secondary structures of 23-MB231 and its variants. The sequence and estimated secondary structures of 23-MB231(DsDs49) are shown on the left, and each variant is schematically represented on the right, along with its dissociation constant (K_D) to the target cell MDA-MB-231. The region modified with the mini-hairpin DNA is indicated in blue. The G–T pairing is indicated with light grey lines.

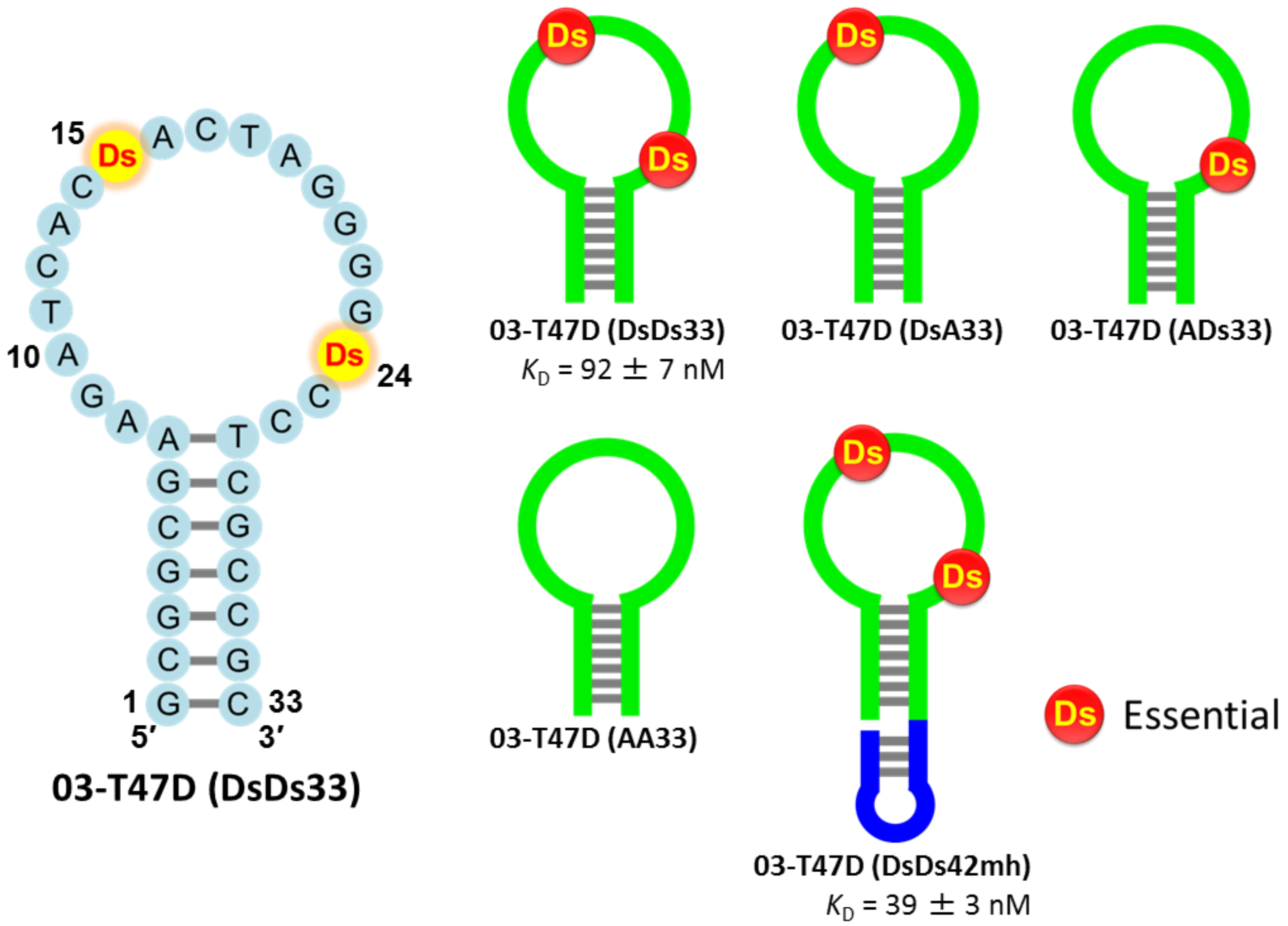

Figure S13: Schematic illustration of the secondary structures of 03-T47D and its variants. The sequence and estimated secondary structures of 03-T47D(DsDs33) are shown on the left, and each variant is schematically represented on the right, along with its dissociation constant (K_D) to the target cell T-47D. The region modified with the mini-hairpin DNA is indicated in blue. In the DsA33, ADs33, and AA33 variants, the corresponding Ds base is replaced with A. The importance of the Ds bases for the binding was judged using the flow cytometry analysis results (see Figure S14).

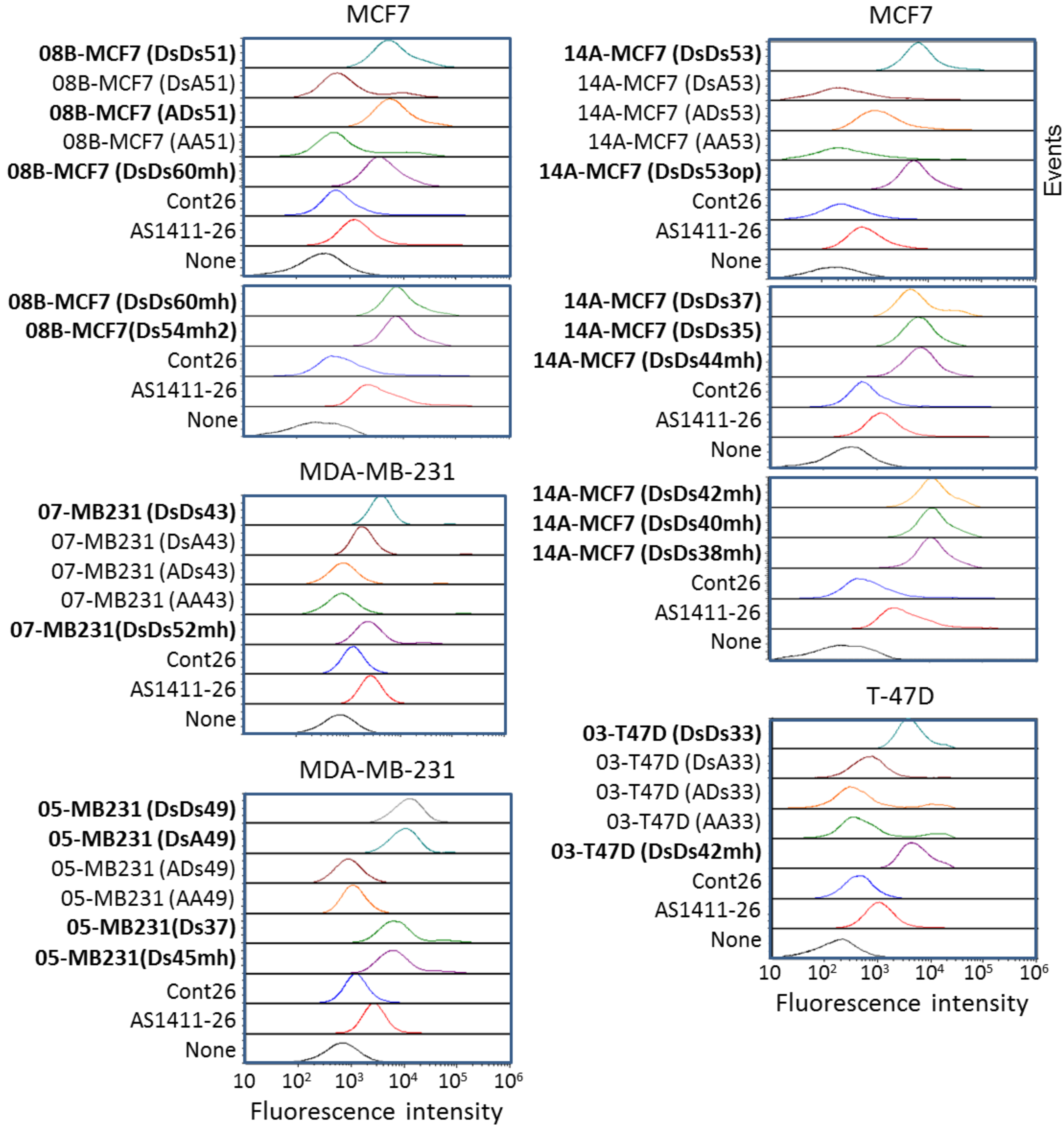

Figure S14: Binding of Ds-DNA aptamers and their variants to target cell lines. The target cells were incubated with the Alexa-488 labeled Ds-DNA aptamer and its variants (250 nM, the sequences are shown in Figures S8–S13) for 30 min at 4ºC, and then analyzed by flow cytometry.

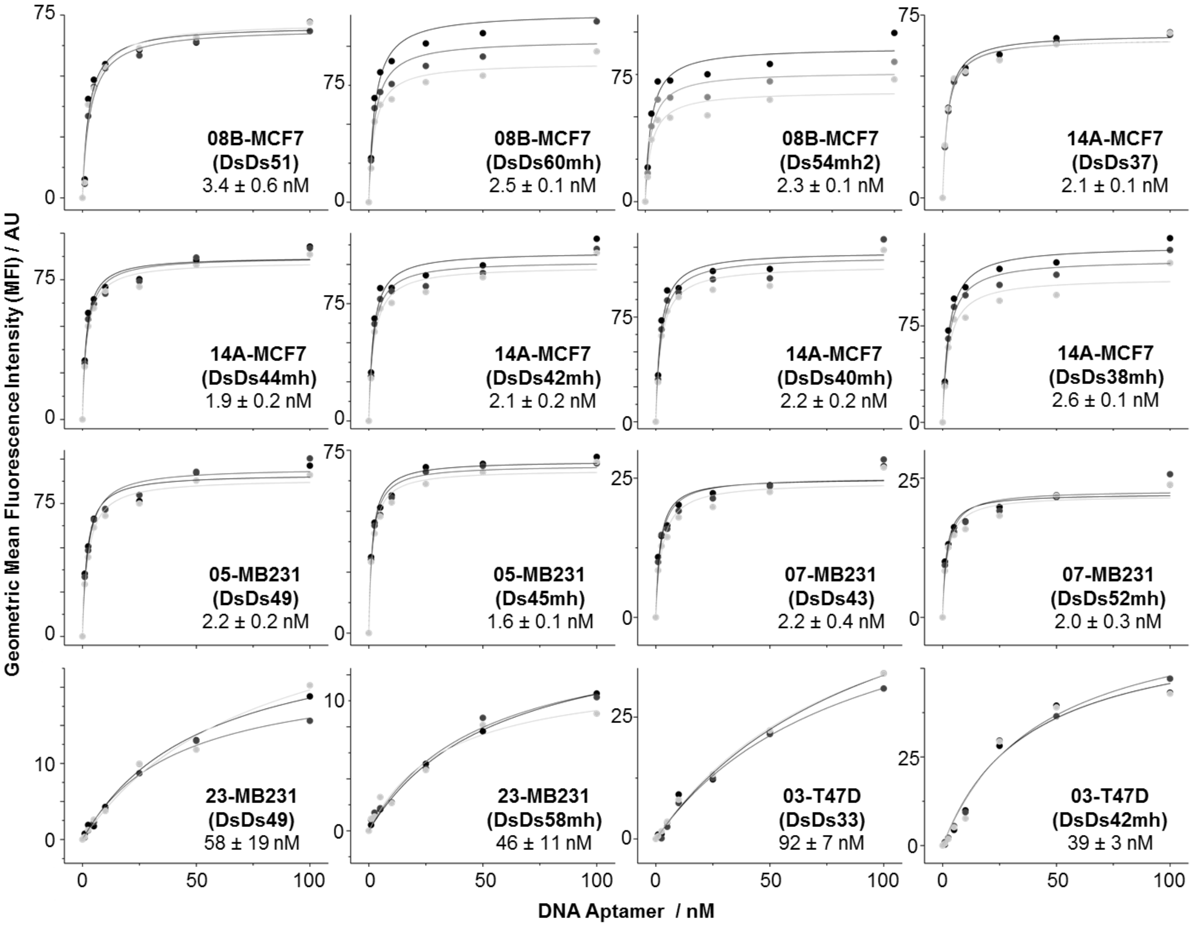

Figure S15: Binding of Ds-DNA aptamers and their variants to target cell lines. The target cells were incubated with the Alexa-488 labeled Ds-DNA aptamer and its variants (1 to 100 nM, the sequences are shown in Figures S8–S13) for 30 min at 4ºC, followed by flow cytometry analyses. The geometric mean fluorescence intensities were plotted against the aptamer concentrations, and the K_D values (average of three experiments) obtained from the curve fitting are shown with the standard deviations.

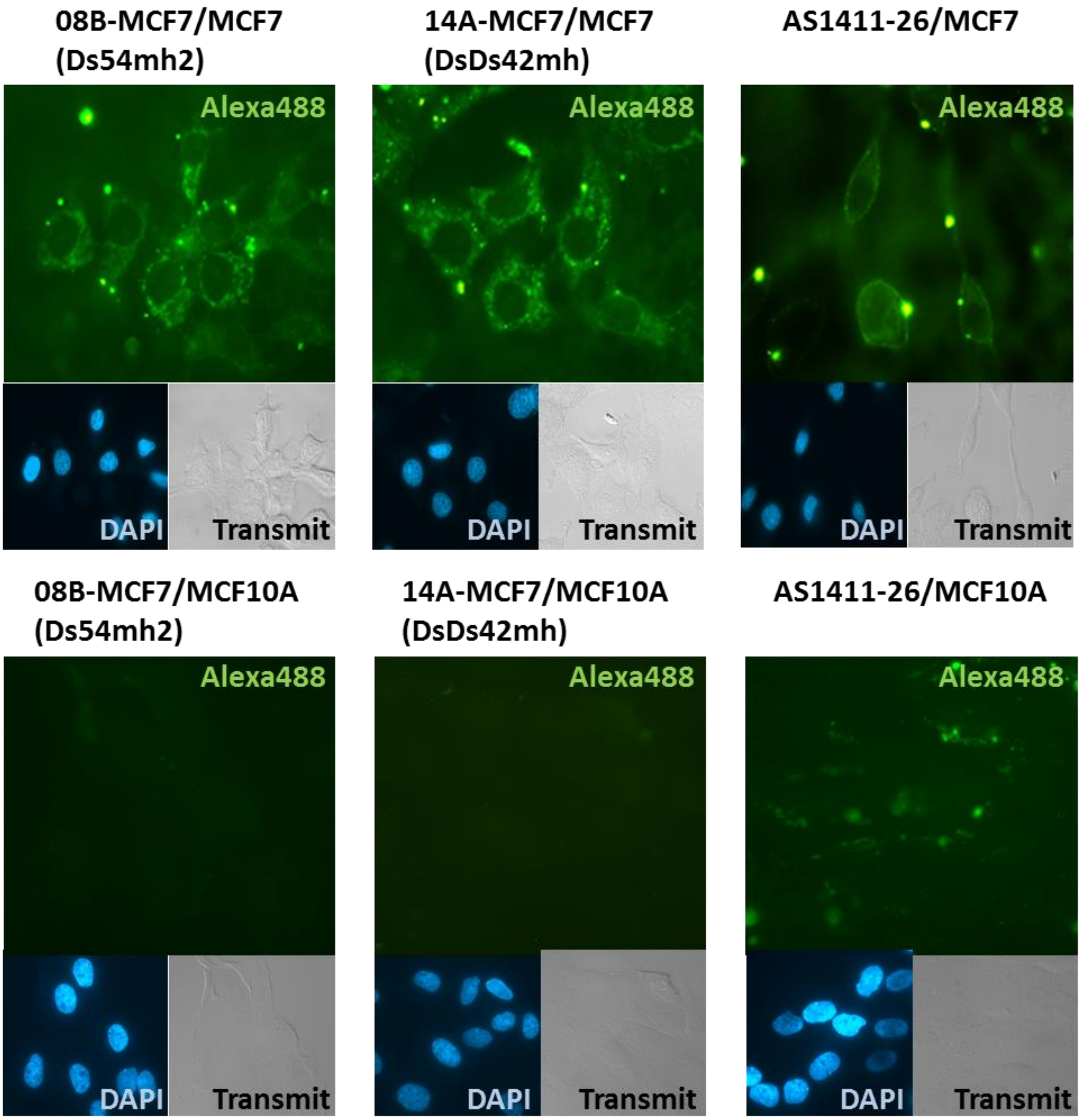

Figure S16: Ds-DNA aptamers 08B-MCF7(Ds54mh2) and 14A-MCF7(DsDs42mh) bound to MCF7 cells, but not to MCF10A cells. MCF7 and MCF10A cells were incubated with 250 nM of Alexa-488 labeled 08B-MCF7(Ds54mh2), 14A-MCF7(DsDs42mh), and AS1411-26 for 30 min at 4ºC, and analyzed by fluorescent microscopy (Alexa488, top; DAPI, lower left; bright-field, lower right).

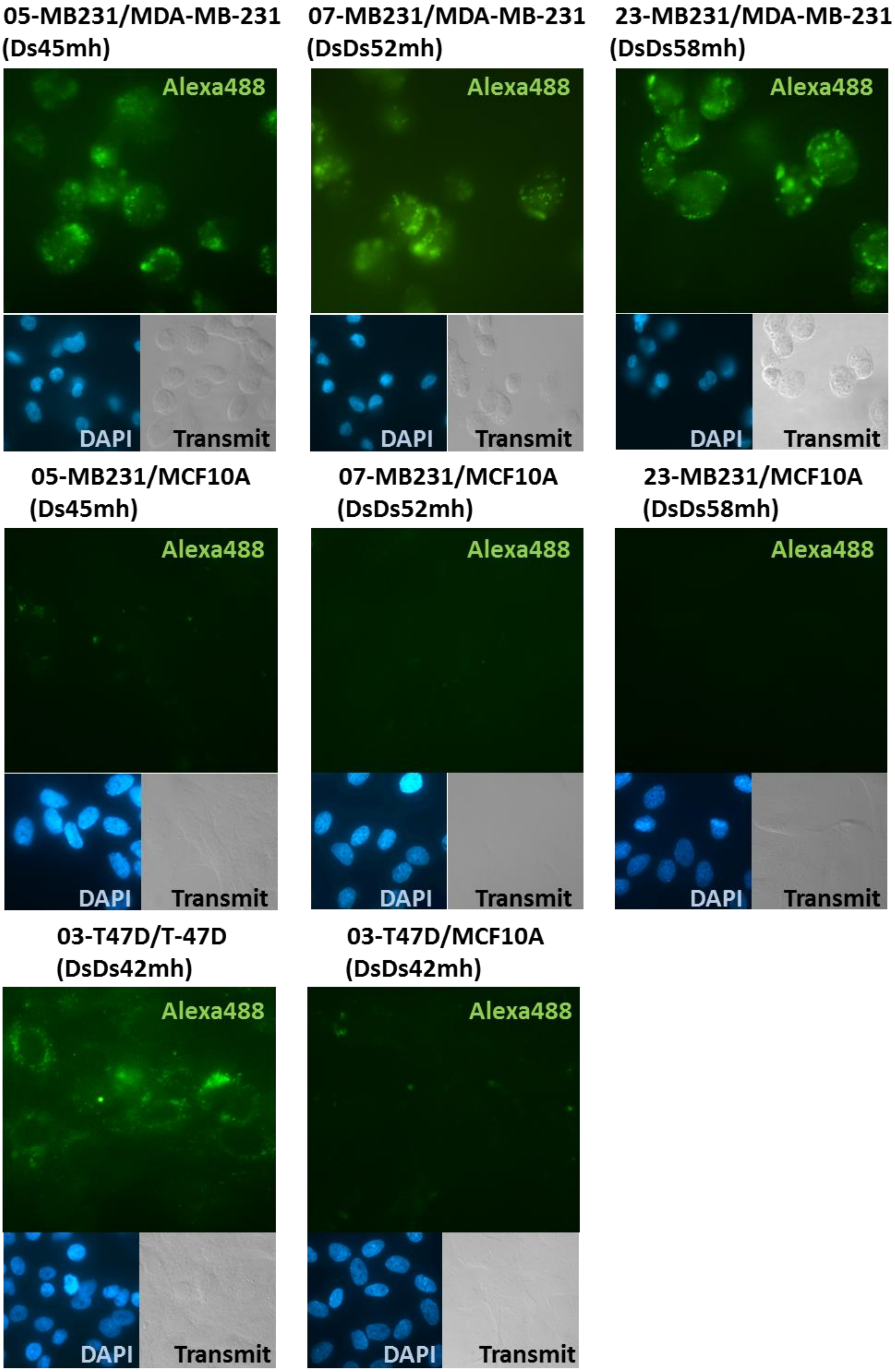

Figure S17: Ds-DNA aptamers 05-MB231(Ds45mh), 07-MB231(DsDs52mh), 23-MB231(DsDs58mh), and 03-T47D(DsDs42mh) bound to the target cells, but not to MCF10A cells. The target cells, MDA-MB-231 and T-47D, as well as MCF10A, were incubated with 250 nM of Alexa-488 labeled 05-MB231 (Ds45mh), 07-MB231 (DsDs52mh), 23-MB231 (DsDs58mh), and 03-T47D (DsDs42mh) for 30 min at 4ºC, and analyzed by fluorescent microscopy (Alexa488, top; DAPI, lower left; bright-field, lower right).

A

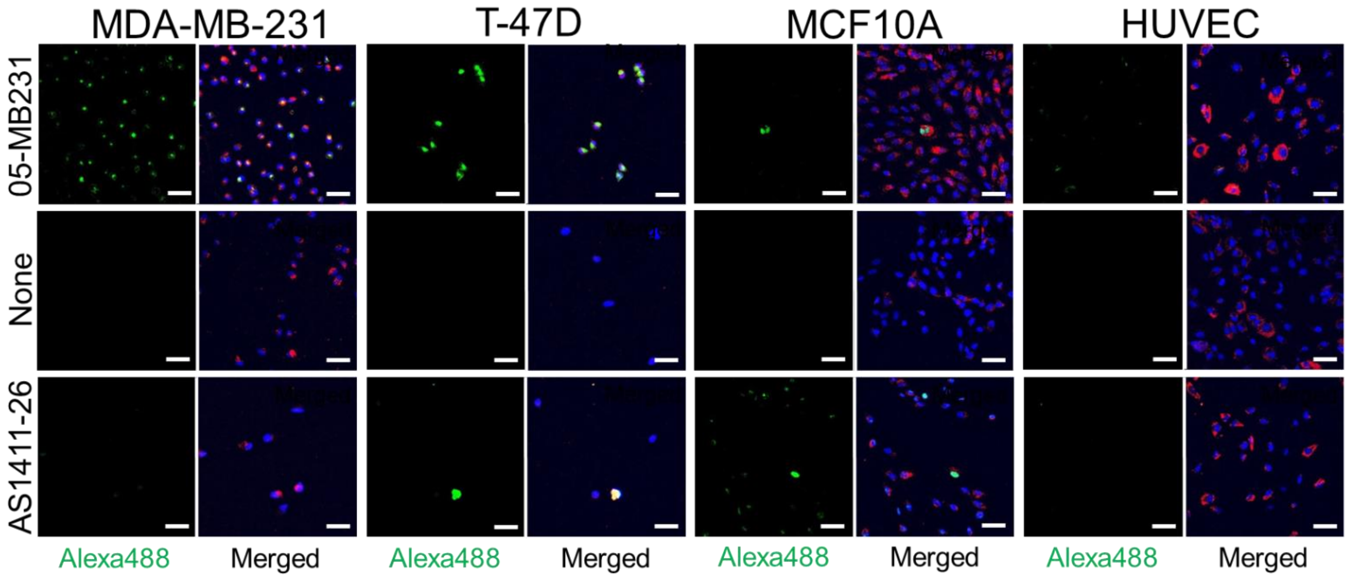

B

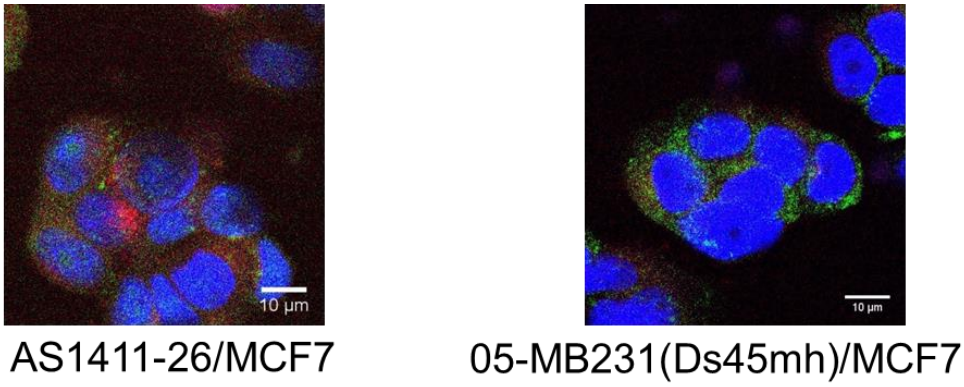

Figure S18: Confocal microscopy imaging. Ds-DNA aptamer 05-MB231(Ds45mh) bound to MDA-MB-231 and T-47D cells more tightly than AS1411 (A), and 05-MB231(Ds45mh) was internalized within the cancer cells (A, B). The cells were incubated in the presence or absence of 250 nM Alexa488-labeled 05-MB231 (Ds45mh) or AS1411-26 for 30 min at 37°C, after the incubation with LysoTracker (for 45 min) to detect acidic organelles inside the cell. The cells were then stained with DAPI and observed under 400 × magnification (A) or under 600 × magnification (B). In the merged images, the DAPI (nuclei, blue), Alexa488 (aptamer, green), and LysoTracker (red) signal images are overlaid. Scale bars: 50 μm (A) and 10 μm (B).

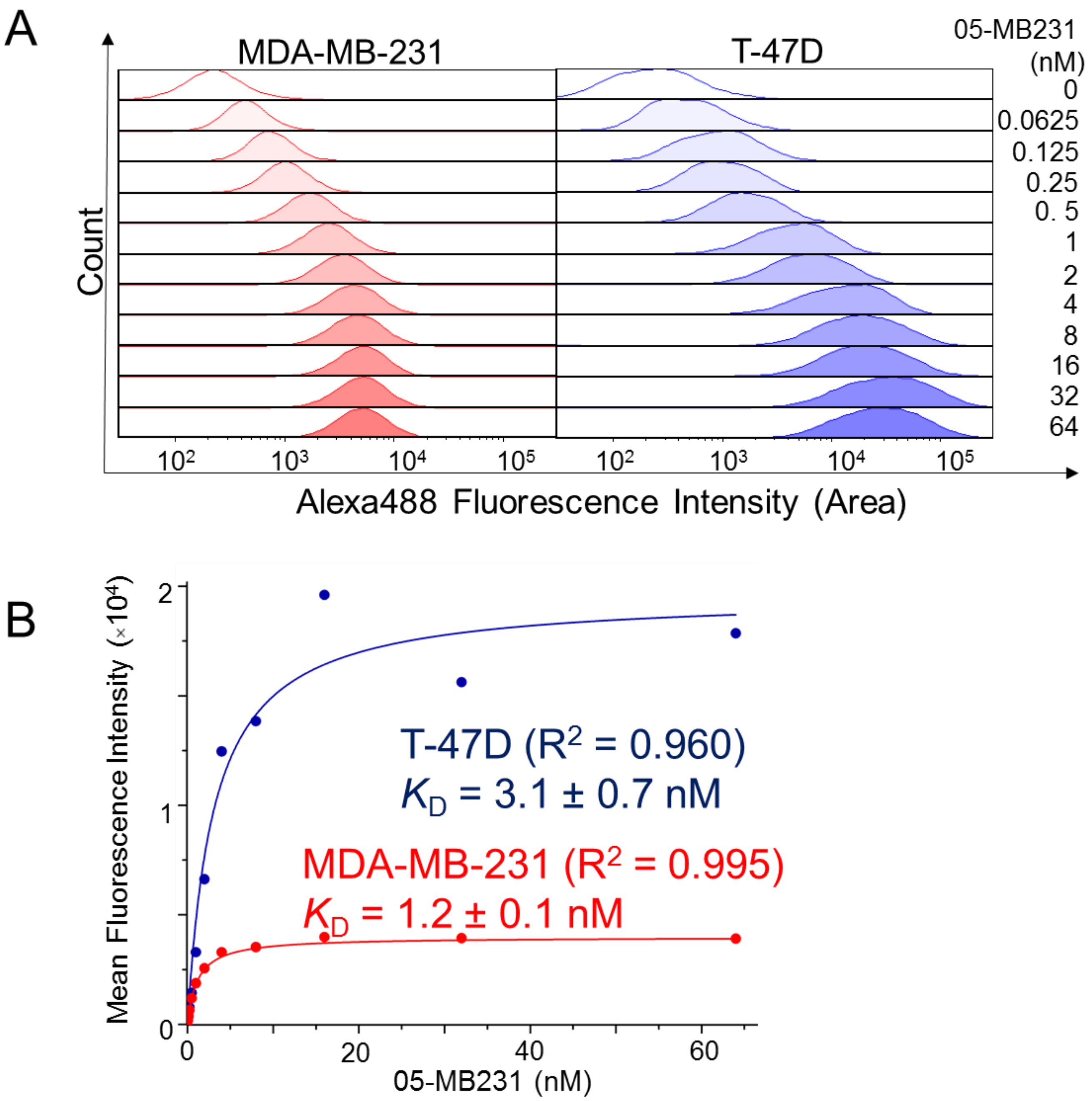

Figure S19: 05-MB231(Ds45mh) binding to MDA-MB-231 and T-47D cells. (A) Binding of 05-MB231(Ds45mh) to MDA-MB-231 (red) and T-47D (blue) cells was analyzed by flow cytometry. 05-MB231(Ds45mh) exhibited low binding affinity at low concentrations (0.0625 nM). (B) Binding curves of 05-MB231(Ds45mh) to MDA-MB-231 (red) and T-47D (blue) cells, showing that 05-MB231(Ds45mh) exhibits similar high binding affinities (K_D = 1–3 nM) to both MDA-MB-231 and T-47D cells.

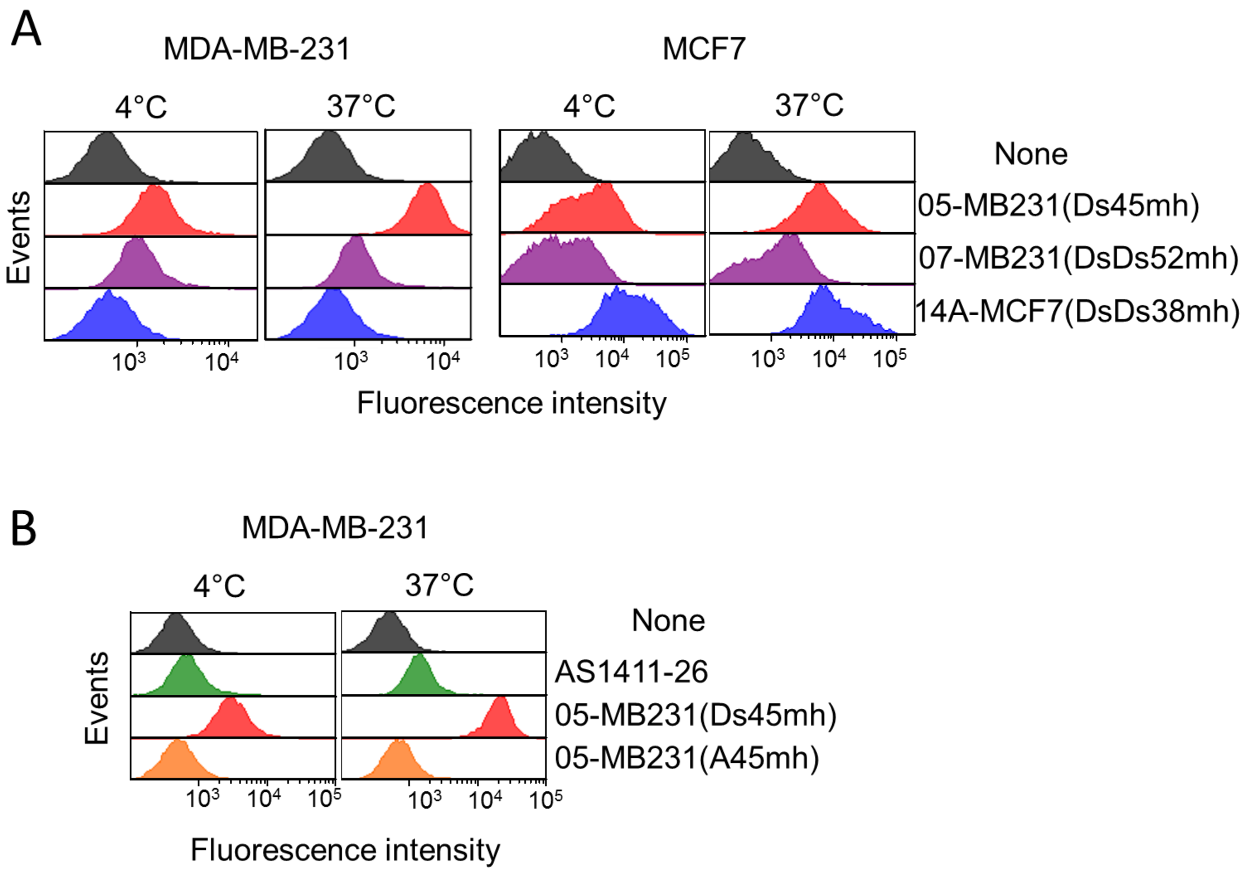

Figure S20: Ds-DNA aptamer binding analysis by flow cytometry. (A) Binding of FAM-labeled Ds-DNA aptamers, 05-MB231(Ds45mh), 07-MB231(DsDs52mh), and 14A-MCF7(DsDs38mh) to MDA-MB-231 and MCF7 cells was analyzed by flow cytometry, after a 30-min incubation at either 4°C or 37°C. 05-MB231(Ds45mh) exhibited higher binding to MDA-MB-231 at 37°C, than at 4°C. (B) Binding of Alexa488-labeled AS1411-26 and 05-MB231(Ds45mh) aptamers to MDA-MB-231 at either 4°C or 37°C. The binding efficiencies increased at 37°C, especially for 05-MB231, as compared with those at 4°C. The Ds-to-A variant (A45mh) did not bind to the cells.

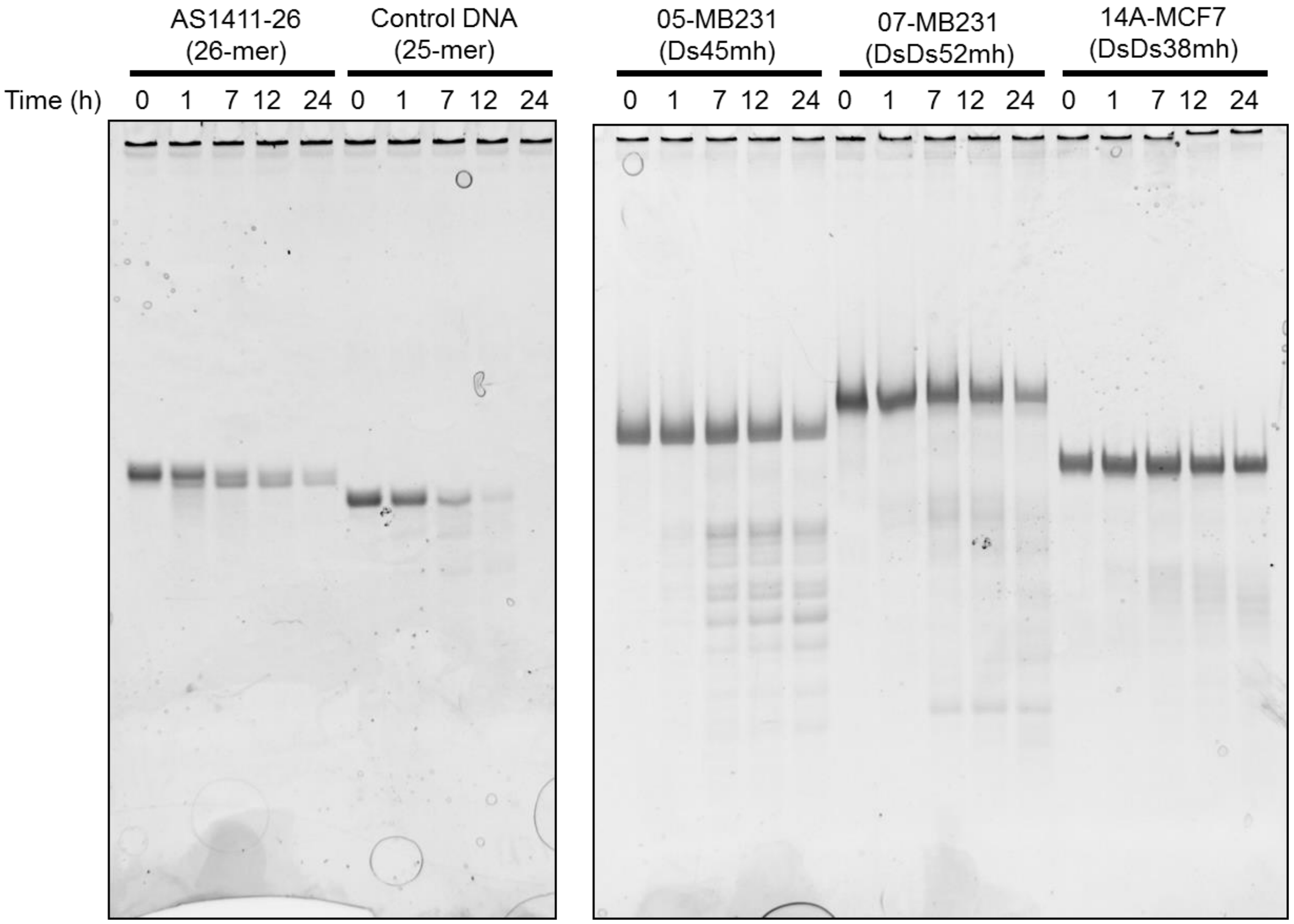

Figure S21: Stability analysis of Ds-DNA aptamers in human serum. Each DNA (250 pmol, 5 µl in 1× D-PBS) was mixed with 120 µl of human serum and incubated at 37°C. After the indicated incubation time, aliquots (10 µl) were obtained from the solutions, immediately mixed with 120 µl of denaturing solution (1× TBE with 10 M urea), and then frozen until PAGE analysis. The prepared samples were denatured at 75°C for 3 min and then a portion (5 µl) was subjected to 15% denaturing PAGE. The DNA on the gels was stained with SYBR Gold and detected with a bio-imaging analyzer (Fuji Film LAS-4000). The sequence of the Control DNA (25-mer) is 5′-CGACGGCGAACCTCCAAGCGGCAGC-3′.

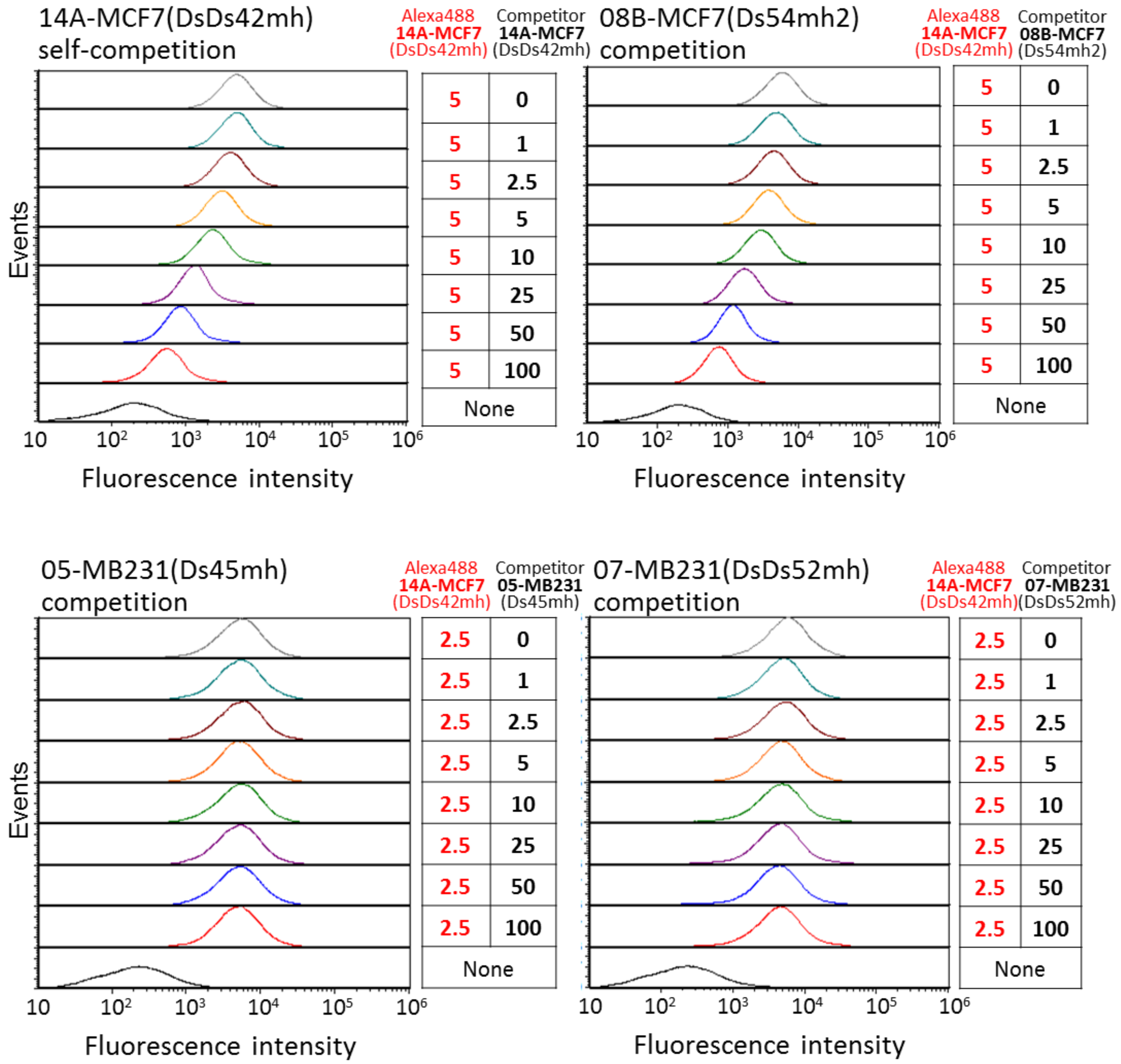

Figure S22: Competitive binding assays. Alexa488-labeled 14A-MCF7(DsDs42mh) (5 or 2.5 nM) was incubated with MCF7 cells, in the presence of non-labeled Ds-DNA aptamer (1 to 100 nM) at 4°C for 30 min, and analyzed by flow cytometry to quantify the Alexa488-labeled 14A-MCF7(DsDs42mh) bound to MCF7 cells.

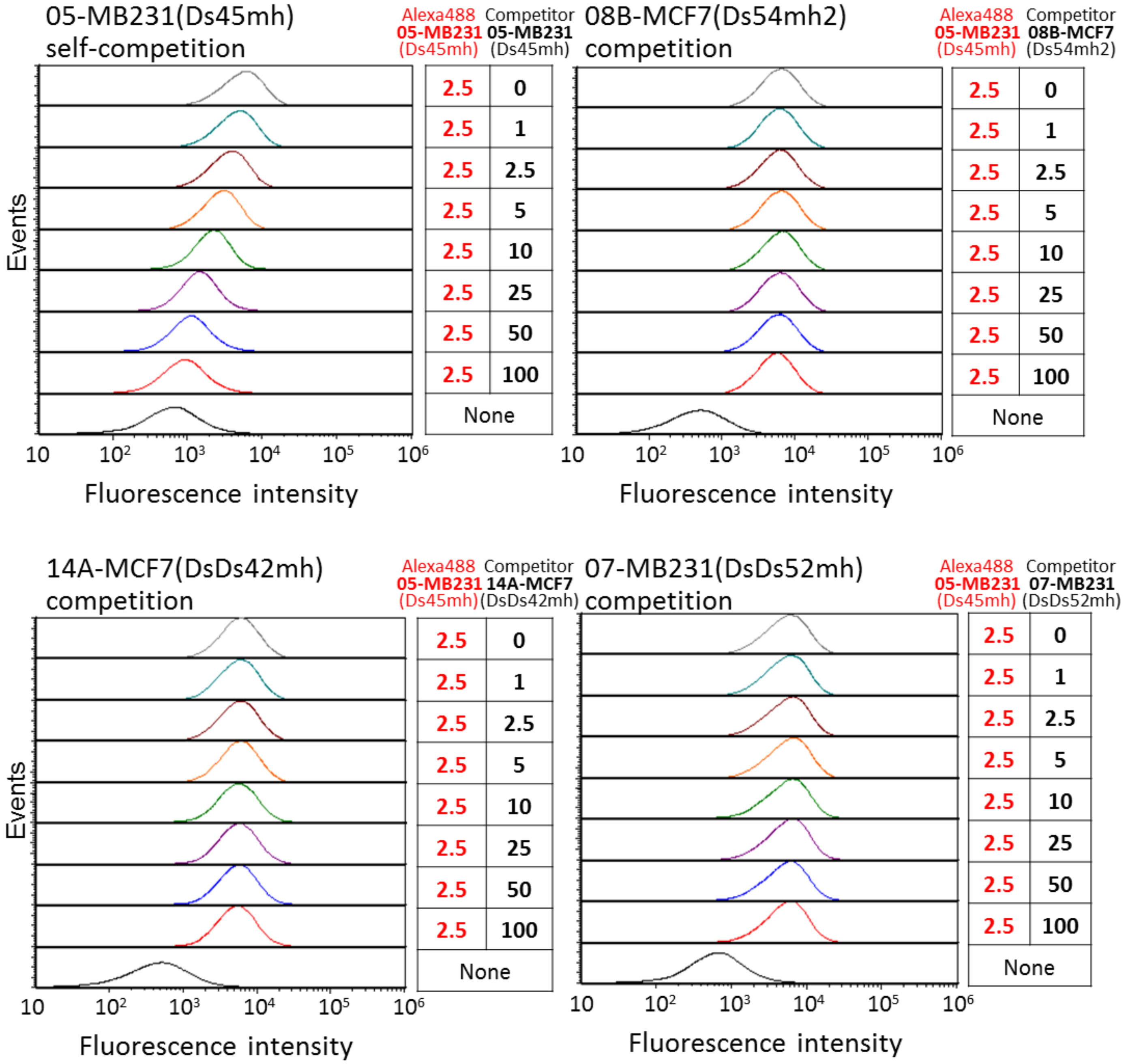

Figure S23: Competitive binding assays. Alexa488-labeled 05-MB231(Ds45mh) (2.5 nM) was incubated with MDA-MB-231 cells, in the presence of non-labeled Ds-DNA aptamer (1 to 100 nM) at 4°C for 30 min, and analyzed by flow cytometry to quantify the Alexa488-labeled 05-MB231(Ds45mh) bound to MDA-MB-231 cells.

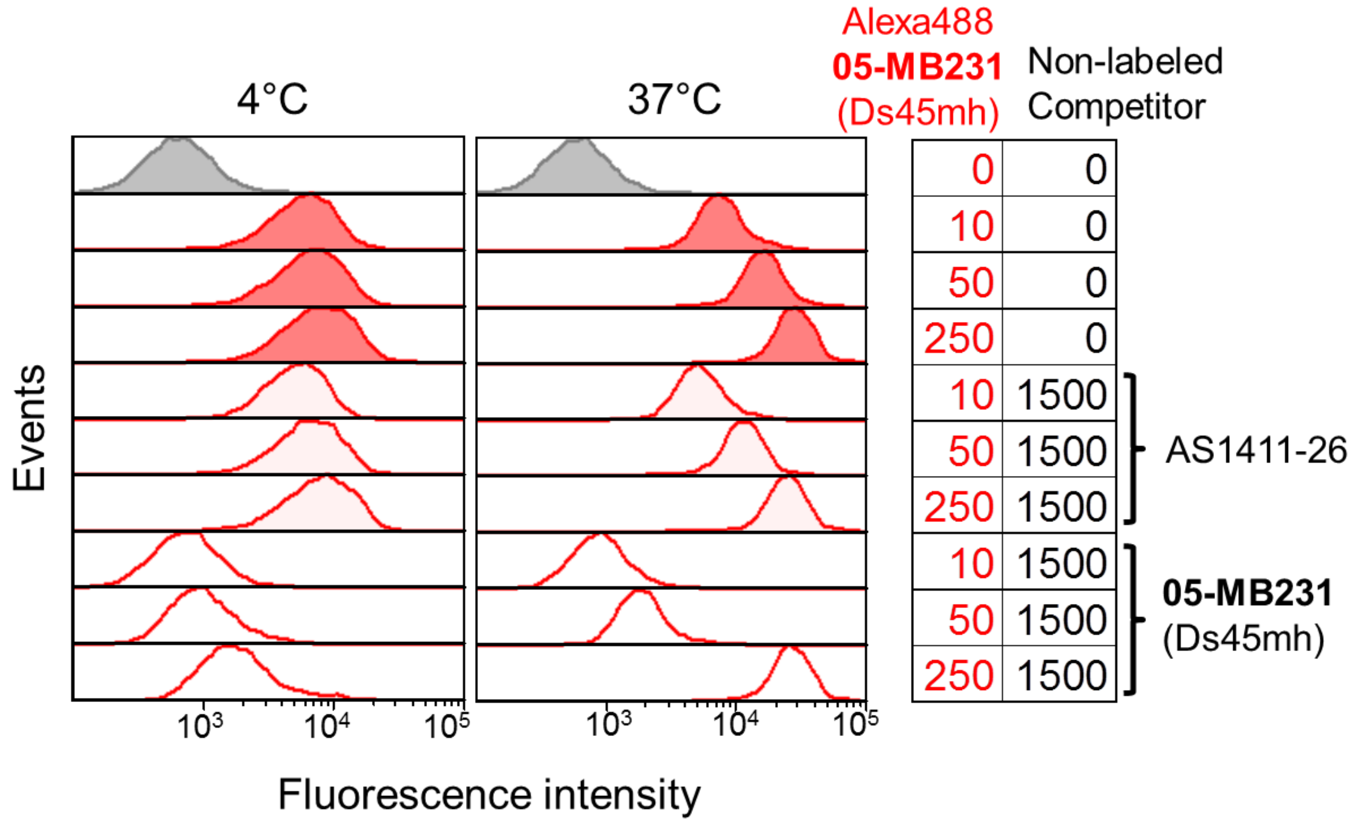

Figure S24: Competitive binding assays. Alexa488-labeled 05-MB231(Ds45mh) (10, 50, or 250 nM) was incubated with MDA-MB-231 cells, in the presence of 1,500 nM non-labeled AS1411-26 or 05-MB231(Ds45mh) at either 4°C or 37°C for 30 min, and analyzed by flow cytometry to quantify the Alexa488-labeled 05-MB231(Ds45mh) bound to MDA-MB-231 cells.

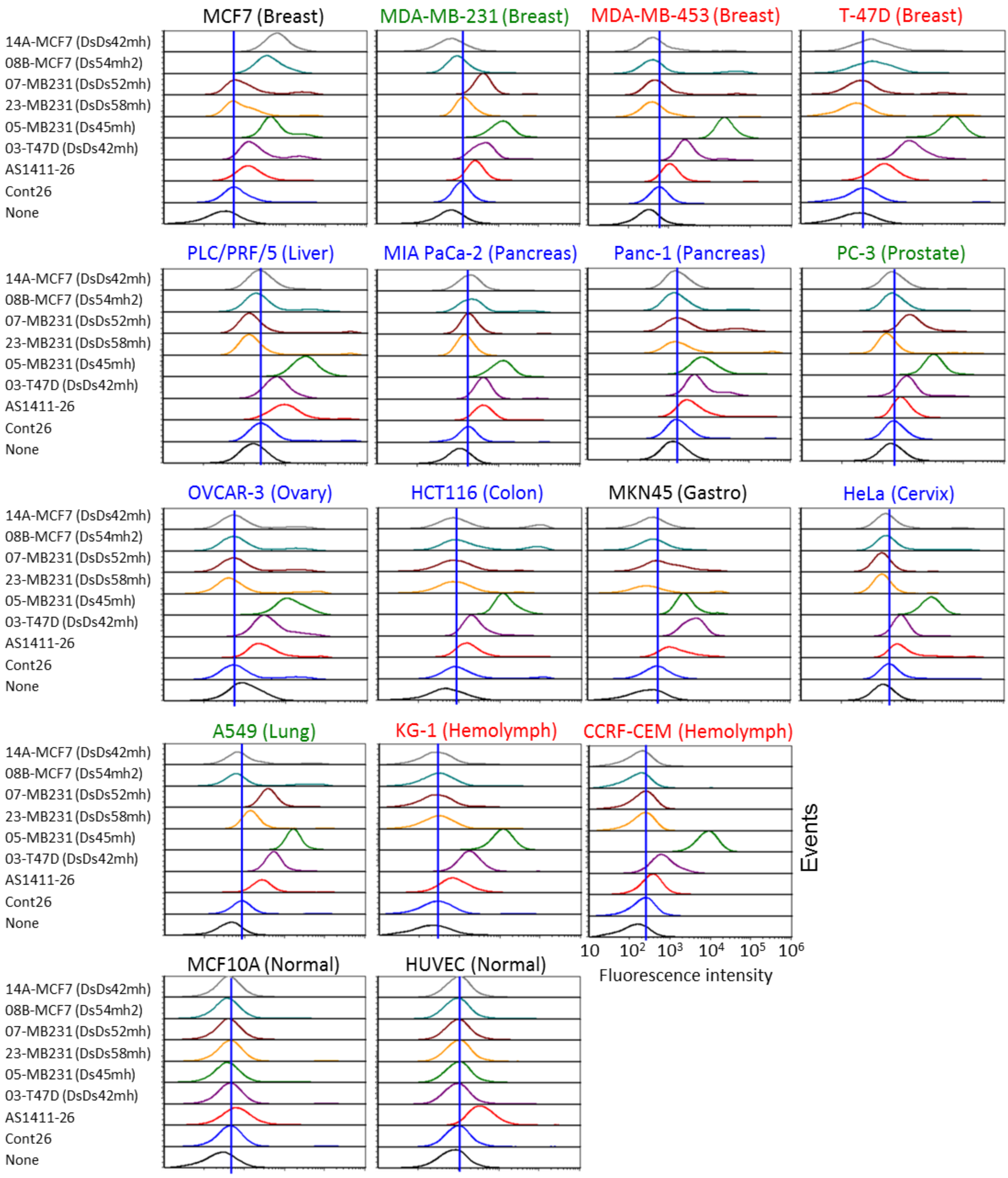

Figure S25: Binding of each Ds-DNA aptamer and AS1411-26 to various cell lines. Fifteen cancer cell lines and two normal cell lines were incubated with 250 nM of each Alexa488-labeled Ds-DNA aptamer, as well as AS1411-26 and Cont26 as controls, at 4°C for 30 min, and analyzed by flow cytometry. Based on the patterns of the fluorescent signals, the cancer cell lines were classified into four categories (the cell lines indicated with green, red, blue, and black). The blue straight line in each panel corresponds to the median fluorescence intensity for Cont26.

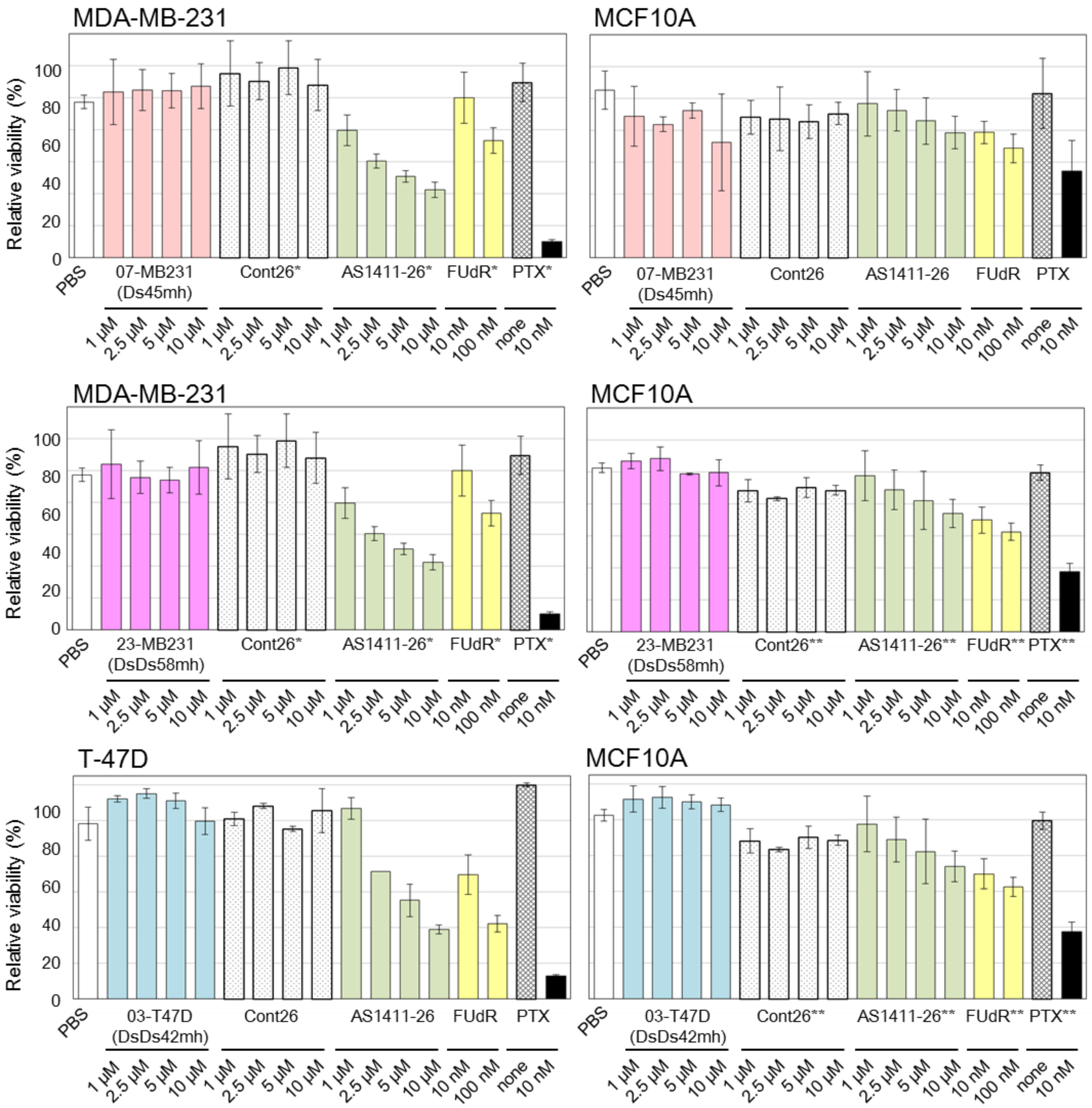

Figure S26: The Ds-DNA aptamers 07-MB231(DsDs52mh), 23-MB231(DsDs58mh), and 03-T47D(DsDs42mh) did not inhibit cell proliferation. The target cells, MDA-MB-231 and T-47D, as well as MCF10A as a control, were cultured in the presence of 07-MB231(DsDs52mh), 23-MB231(DsDs58mh), or 03-T47D(DsDs42mh) for 96 hours, followed by a WST-8 assay to determine relative cell viability. The assays were performed independently twice, and then the relative viabilities were averaged. The error bars represent standard deviations. *, **: The same data were used in the graphs for easy comparison, since the assays using 07-MB231(DsDs52mh) and 23-MB231(DsDs58mh) against MDA-MB-231 cells and those using 23-MB231(DsDs58mh) and 03-T47D(DsDs42mh) were performed within the same 96-well plates. FUdR: 5-fluorodeoxyuridine, PTX: Paclitaxel.

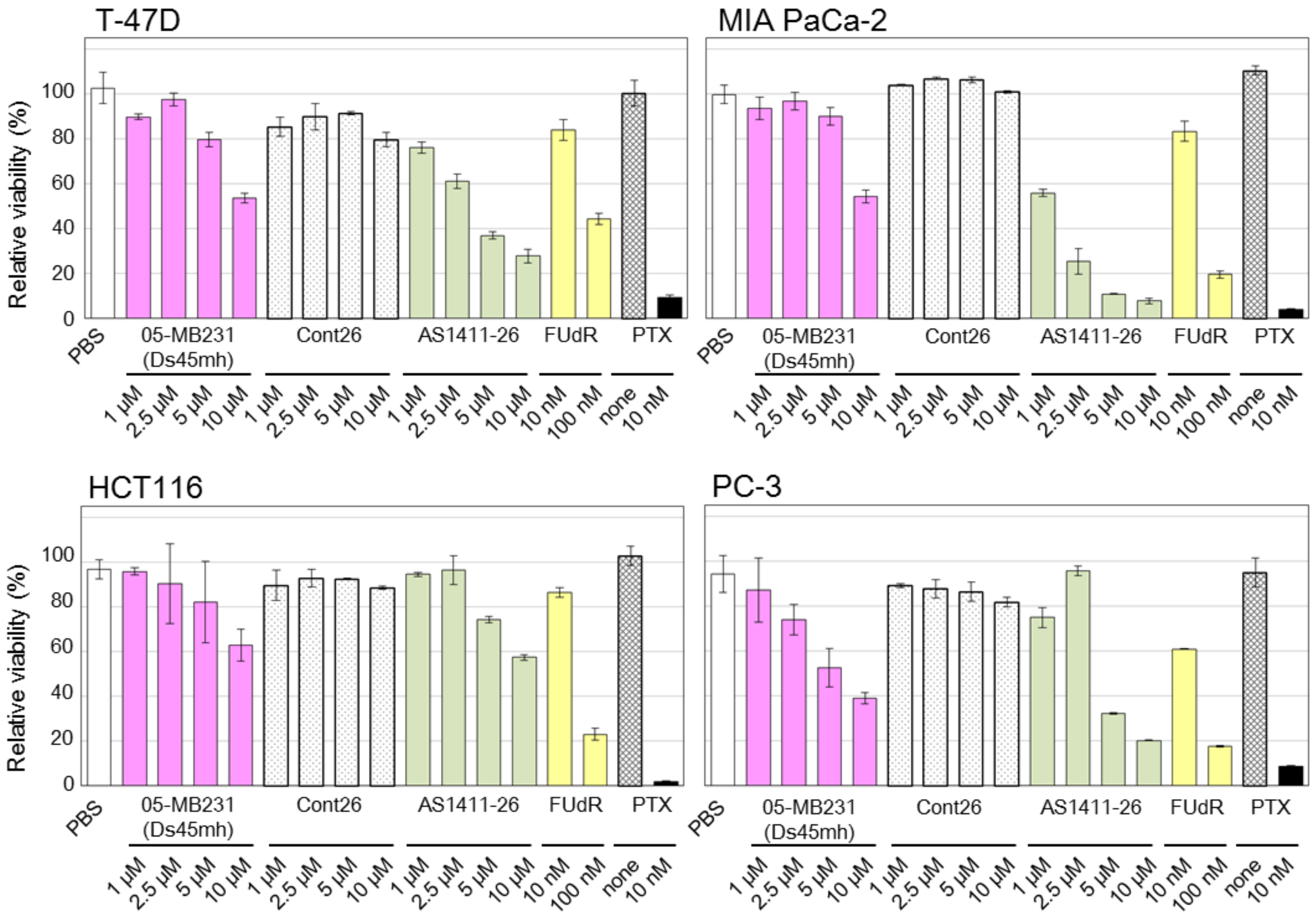

Figure S27: The Ds-DNA aptamer 05-MB231(Ds45mh) inhibited cancer cell proliferation. Several cancer cell lines were cultured in the presence of 05-MB231(Ds45mh) for 96 hours, followed by a WST-8 assay to determine relative cell viability. The assays were performed independently twice, and then the relative viabilities were averaged. The error bars represent standard deviations. FUdR: 5-fluorodeoxyuridine, PTX: Paclitaxel.

Table S1: DNA sub-libraries and PCR primers used in cell-ExSELEX. In each sub-library, barcode sequences (three bases) and random regions containing two Ds bases (red) are extracted and shown (N = a mixture of A, G, C, and T). A primer set, 5′-Rev019 and 3′T15-L-Fow026, was used in PCR during cell-ExSELEX for the Ds-strand separation. Another primer set, 5′-Rev019 and 3′-Fow026, was used for sequencing sample preparation.

Name	Sequence
N42Ds-01	5'-GAGNNNNNNNNNNNNNNNNNNNNNDsNNNNNNDsNNNNNNNNNNNNN-3'
N42Ds-02	5'-GACNNNNNNNNNNNNNNNNNNNNDsNNNNNNNDsNNNNNNNNNNNNN-3'
N42Ds-03	5'-CAGNNNNNNNNNNNNNNNNNNNDsNNNNNNNNDsNNNNNNNNNNNNN-3'
N42Ds-04	5'-CACNNNNNNNNNNNNNNNNNNDsNNNNNNNNNDsNNNNNNNNNNNNN-3'
N42Ds-05	5'-AGGNNNNNNNNNNNNNNNNNDsNNNNNNNNNNDsNNNNNNNNNNNNN-3'
N42Ds-06	5'-GGANNNNNNNNNNNNNNNNDsNNNNNNNNNNNDsNNNNNNNNNNNNN-3'
N42Ds-07	5'-GGGNNNNNNNNNNNNNNNDsNNNNNNNNNNNNDsNNNNNNNNNNNNN-3'
N42Ds-08	5'-GGCNNNNNNNNNNNNNNDsNNNNNNNNNNNNNDsNNNNNNNNNNNNN-3'
N42Ds-09	5'-GGTNNNNNNNNNNNNNDsNNNNNNNNNNNNNNDsNNNNNNNNNNNNN-3'
N42Ds-10	5'-CGANNNNNNNNNNNNNDsNNNNNNDsNNNNNNNNNNNNNNNNNNNNN-3'
N42Ds-11	5'-CGGNNNNNNNNNNNNNDsNNNNNNNDsNNNNNNNNNNNNNNNNNNNN-3'
N42Ds-12	5'-CGCNNNNNNNNNNNNNDsNNNNNNNNDsNNNNNNNNNNNNNNNNNNN-3'
N42Ds-13	5'-CGTNNNNNNNNNNNNNDsNNNNNNNNNDsNNNNNNNNNNNNNNNNNN-3'
N42Ds-14	5'-TGGNNNNNNNNNNNNNDsNNNNNNNNNNDsNNNNNNNNNNNNNNNNN-3'
N42Ds-15	5'-TGCNNNNNNNNNNNNNDsNNNNNNNNNNNDsNNNNNNNNNNNNNNNN-3'
N42Ds-16	5'-ACGNNNNNNNNNNNNNDsNNNNNNNNNNNNDsNNNNNNNNNNNNNNN-3'
N42Ds-17	5'-GCANNNNNNNNNNNNNDsNNNNNNNNNNNNNDsNNNNNNNNNNNNNN-3'
N42Ds-18	5'-GCGNNNNNNNNNNNNNNNNNDsNNNNNNDsNNNNNNNNNNNNNNNNN-3'
N42Ds-19	5'-GCCNNNNNNNNNNNNNNNNDsNNNNNNNDsNNNNNNNNNNNNNNNNN-3'
N42Ds-20	5'-GCTNNNNNNNNNNNNNNNNDsNNNNNNNNDsNNNNNNNNNNNNNNNN-3'
N42Ds-21	5'-CCANNNNNNNNNNNNNNNDsNNNNNNNNNDsNNNNNNNNNNNNNNNN-3'
N42Ds-22	5'-CCGNNNNNNNNNNNNNNNDsNNNNNNNNNNDsNNNNNNNNNNNNNNN-3'
N42Ds-23	5'-CCCNNNNNNNNNNNNNDsNNNNNNNNNNNNDsNNNNNNNNNNNNNNN-3'
N42Ds-24	5'-CCTNNNNNNNNNNNNNNDsNNNNNNNNNNNNDsNNNNNNNNNNNNNN-3'
5'-Rev019	5'-ACGACCGTTCTCTAATTTTGACGTT-3'
3'T15-L-Fow026	5'-TTTTTTTTTTTTTTT-C12spacer-ACCAAATTATTGCGATACAGACCCT-3'
3'-Fow026	5'-ACCAAATTATTGCGATACAGACCCT-3'

Table S2: Conditions of cell-ExSELEX targeting MCF7 cells. Conditions for the initial selection and the doped selections for each isolated aptamer (14A or 08B) are summarized. Pre- or post-counter selections were performed using MCF10A cells.

Library	Round	DNA (nM)	Volume (ml)	Cell	Binding	Time (min)	Wash	PCR cycles
N42Ds	1	1000	2	MCF7 (100 mm dish ×1)	4°C	60	5 ml × 5 times	15
	pre-2	50	2	MCF10A (100 mm dish ×1)	4°C	60		
	2	50	2	MCF7 (100 mm dish ×1)	4°C	30	5 ml / 5 min × 5 times	22
	pre-3	5	2	MCF10A (100 mm dish ×5)	4°C	60		
	3	5	10	MCF7 (100 mm dish ×1)	4°C	30	5 ml / 10 min × 5 times	20
	pre-4	2.5	2	MCF10A (100 mm dish ×5)	r.t.	60		
	4	2.5	10	MCF7 (100 mm dish ×1)	4°C	30	7 ml / 10 min × 5 times	21
	pre-5	1	2	MCF10A (100 mm dish ×5)	r.t.	60		
	5	1	10	MCF7 (100 mm dish ×1)	4°C	30	10 ml / 10 min × 5 times	20
	pre-6	1	2	MCF10A (100 mm dish ×5)	r.t.	60		
	6	1	10	MCF7 (100 mm dish ×1)	4°C	30	10 ml / 10 min × 5 times	18
	post-6		1	MCF10A (100 mm dish ×1)	r.t.	60		
	pre-7	1	2	MCF10A (100 mm dish ×5)	37°C	60		
	7	1	10	MCF7 (100 mm dish ×1)	4°C	30	10 ml / 10 min × 5 times	18
	post-7		1	MCF10A (100 mm dish ×1)	37°C	60		
Doped-14A-MCF7	1	50	10	MCF7 (100 mm dish ×1)	4°C	60	5 ml × 3 times	22
	2	5	10	MCF7 (100 mm dish ×1)	4°C	45	5 ml / 5 min × 3 times	25
	3	2	5	MCF7 (100 mm dish ×1)	4°C	30	5 ml / 5 min × 3 times	28
	4	1	5	MCF7 (100 mm dish ×1)	4°C	30	5 ml / 5 min x 5times	21
Doped-08B-MCF7	1	50	10	MCF7 (100 mm dish ×1)	4°C	60	5 ml × 3 times	21
	2	5	10	MCF7 (100 mm dish ×1)	4°C	45	5 ml / 5 min × 3 times	25
	3	2	5	MCF7 (100 mm dish ×1)	4°C	30	5 ml / 5 min × 3 times	25
	4	1	5	MCF7 (100 mm dish ×1)	4°C	30	5 ml / 5 min × 5 times	16

Table S3: Conditions of cell-ExSELEX targeting MDA-MB-231 cells. Conditions for the initial selection and the doped selections for each isolated aptamer (05, 07 or 23) are summarized. Pre-counter selections were performed using MCF10A cells.

Library	Round	DNA (nM)	Volume (ml)	Cell	Binding	Time (min)	Wash	PCR cycles
N42Ds	1	50	20	MDA-MB-231 (100 mm dish ×1)	4°C	60	5 ml × 5 times	14
	2	10	10	MDA-MB-231 (100 mm dish ×1)	4°C	60	5 ml / 5 min × 5 times	15
	pre-3	5	10	MCF10A (150 mm dish ×1)	4°C	60		
	3	5	10	MDA-MB-231 (100 mm dish ×1)	4°C	45	5 ml / 10 min × 5 times	21
	pre-4	2.5	10	MCF10A (150 mm dish ×1)	4°C	60		
	4	2.5	10	MDA-MB-231 (100 mm dish ×1)	4°C	30	7 ml / 10 min × 5 times	23
	pre-5	2.5	10	MCF10A (150 mm dish ×1)	4°C	60		
	5	2.5	10	MDA-MB-231 (100 mm dish ×1)	4°C	30	10 ml / 10 min × 5 times	21
	pre-6	1	10	MCF10A (150 mm dish ×1)	4°C	60		
	6	1	10	MDA-MB-231 (100 mm dish ×1)	4°C	30	10 ml / 10 min × 5 times	24
	pre-7	1	5	MCF10A (150 mm dish ×1)	4°C	60		
	7	1	5	MDA-MB-231 (100 mm dish ×1)	4°C	30	10 ml / 10 min × 5 times	19
Doped-05-MB231	1	50	10	MDA-MB-231 (100 mm dish ×1)	4°C	60	5 ml × 3 times	18
	2	5	10	MDA-MB-231 (100 mm dish ×1)	4°C	45	5 ml / 5 min × 3 times	21
	3	2	5	MDA-MB-231 (100 mm dish ×1)	4°C	30	5 ml / 5 min × 3 times	24
	4	1	5	MDA-MB-231 (100 mm dish ×1)	4°C	30	5 ml / 5 min × 5 times	21
Doped-07-MB231	1	50	10	MDA-MB-231 (100 mm dish ×1)	4°C	60	5ml × 3 times	15
	2	5	10	MDA-MB-231 (100 mm dish ×1)	4°C	45	5 ml / 5 min × 3 times	24
	3	2	5	MDA-MB-231 (100 mm dish ×1)	4°C	30	5 ml / 5 min × 3 times	24
	4	1	5	MDA-MB-231 (100 mm dish ×1)	4°C	30	5 ml / 5 min × 5 times	21
Doped-23-MB231	1	50	10	MDA-MB-231 (100 mm dish ×1)	4°C	60	5ml × 3 times	15
	2	5	10	MDA-MB-231 (100 mm dish ×1)	4°C	45	5 ml / 5 min × 3 times	21
	3	2	5	MDA-MB-231 (100 mm dish ×1)	4°C	30	5 ml /5 min × 3 times	25
	4	1	5	MDA-MB-231 (100 mm dish ×1)	4°C	30	5 ml / 5 min × 5 times	23

Table S4: Conditions of cell-ExSELEX targeting T-47D cells. Conditions for the initial selection and the doped selection for the isolated aptamer (03) are summarized. Pre-counter selections were performed using MCF10A cells.

Library	Round	DNA (nM)	Volume (ml)	Cell	Binding	Time (min)	Wash	PCR cycles
N42Ds	1	50	20	T-47D (100 mm dish ×1)	4 °C	60	5 ml x 5 times	23
	2	10	10	T-47D (100 mm dish ×1)	4 °C	60	5 ml / 5 min × 5 times	24
	pre-3	5	10	MCF10A (150 mm dish ×1)	4 °C	60		
	3	5	10	T-47D (100 mm dish ×1)	4 °C	30	5 ml / 10 min × 5 times	25
	pre-4	2.5	10	MCF10A (150 mm dish ×1)	4 °C	60		
	4	2.5	10	T47D (100 mm dish ×1)	4 °C	30	7 ml / 10 min × 5 times	26
	pre-5	2.5	10	MCF10A (150 mm dish ×1)	4 °C	60		
	5	2.5	10	T-47D (100 mm dish ×1)	4 °C	30	10 ml / 10 min × 5 times	25
	pre-6	1	10	MCF10A (150 mm dish ×1)	4 °C	60		
	6	1	10	T-47D (100 mm dish ×1)	4 °C	30	10 ml / 10 min × 5 times	24
	pre-7	1	5	MCF10A (150 mm dish ×1)	4 °C	60		
	7	1	5	T-47D (100 mm dish ×1)	4 °C	30	10 ml /10 min × 5 times	22
Doped-03-T47D	1	50	10	T-47D (100 mm dish ×1)	4 °C	60	5 ml × 3 times	19
	2	5	10	T-47D (100 mm dish ×1)	4 °C	45	5 ml / 5 min × 3 times	21
	3	2	5	T-47D (100 mm dish ×1)	4 °C	30	5 ml / 5 min × 3 times	20
	4	1	5	T-47D (100 mm dish ×1)	4 °C	30	5 ml / 5 min × 5 times	19

Table S5: Sequences of the barcode and the random region of each clone selected after seven rounds of the initial selection targeting MCF7, MDA-MB-231, and T-47D cells. The Ds positions (red) in each clone were determined from the corresponding barcode sequences. Sequences of the top counts (more than 2% in the analyzed reads) in each cell-ExSELEX are listed.

**MCF7**			
**Total reads**	**246,903**		
**Extracted reads**	**80,592**		
**Analyzed reads (Counts ≥2)**	**21,600**		
**Selected clones**	**Counts**		**Barcode-N42 sequences**
14A-MCF7	9,735	45.1%	tggCCACTCTGTCGTCDsGTCGAACTGCDsATGAGCGTGCTTTGGAG
	1,661	7.7%	cgtCTGGTTGGGATACDsTAGCGCATGDsACCGTTCTCGAAACCCTT
	1,282	5.9%	ccaCCAAAGAGCGCTATTDsTCGTGCATGDsACCATCCTGGACTCTT
	734	3.4%	ggtATCCCCTGGCACADsTATAGACAGGCGTGDsCTATTGGCGCGCA
	684	3.2%	ggcGCGCCCTTTTCGTTDsTTGGTTGGTGGTGDsGGAGAAAAGATGG
	669	3.1%	ggtAGCGGTGTATAGCDsTATGGACTGCAGAGDsCCTTCGCTGACTA
	551	2.6%	agaCGCTACAATAAATDsTTGATCTAACCTDsTATCCTTGGGCATGC
08B-MCF7	484	2.2%	ggcCCGGCTGGCATGTADsTCATGCCTCCTGGDsCTAAGGTTTCTAA
**Target: MDA-MB-231**			
**Total reads**	**497,341**		
**Extracted reads**	**128,607**		
**Analyzed reads (Counts ≥2)**	**95,082**		
**Selected clones**	**Counts**		**Barcode-N42 sequences**
23-MB231	47,092	49.5%	cccTCTTAAGTTTATAGDsTGTCTTTTGGTDsCCTAGGGCGTTTTAA
05-MB231	22,521	23.7%	aggGGTGTTTGTGCCGTGAGDsTGTAGCGTTTDsGAATTTAGGCTTT
07-MB231	18,234	19.2%	gggCCTAGGCAGCTTGGGDsTATCTTTCAGTCDsATTTCAGTCGGCT
	3,001	3.2%	gctTCTGTTTTTCTTTACADsCTTTGCCGDsGGAGGTCTGATTTCTC
**Target: T-47D**			
**Total reads**	**638,160**		
**Extracted reads**	**205,801**		
**Analyzed reads (Counts ≥2)**	**192,728**		
**Selected clones**	**Counts**		**Barcode-N42 sequences**
03-T47D	156,326	81.1%	cagAGTAAGAAACGAAGATCACDsACTAGGGGDsCCTCGAAGTCTAC
	16,436	8.5%	cagGCCAAAGAGCCTAACGCACDsACTAGGGGDsGTGAGCTCTACCC
	4,745	2.5%	cgtTTTTATTAAAGCCDsATTGCATGTDsGACAGACATCCTGTCAAC
