# Supplementary material for: Genetic Alphabet Expansion Provides Versatile Specificities and Activities of Unnatural-Base DNA Aptamers Targeting Cancer Cells
Source: Mol Ther Nucleic Acids. 2018 Nov 29;14:158–70. doi: 10.1016/j.omtn.2018.11.011 (PMC6307347; doi:10.1016/j.omtn.2018.11.011)
Supplement: Document S2. Article plus Supplemental Information [file mmc2.pdf]

Molecular Therapy
Nucleic Acids
Original Article

Genetic Alphabet Expansion Provides Versatile Specificities and Activities of Unnatural-Base DNA Aptamers Targeting Cancer Cells

Kazunobu Futami,[1] Michiko Kimoto,[2,3] Yun Wei Shermane Lim,[3,4] and Ichiro Hirao[2,3]

[1]TAGCyx Biotechnologies, Inc., Komaba Open Laboratory 403, 4-6-1 Komaba, Meguro-ku, Tokyo 153-0041, Japan; [2]RIKEN Center for Life Science Technologies, 1-7-22 Suehiro-cho, Tsurumi-ku, Yokohama, Kanagawa 230-0045, Japan; [3]Institute of Bioengineering and Nanotechnology, 31 Biopolis Way, The Nanos, #07-01, Singapore 138669, Singapore; [4]NUS High School of Mathematics and Science, 20 Clementi Avenue 1, Singapore 129957, Singapore

The potential of genetic alphabet expansion technologies using artificial extra base pairs (unnatural base pairs) has been rapidly expanding and increasing. We present that the hydrophobic unnatural base, 7-(2-thienyl)imidazo[4,5-*b*]pyridine (Ds), which acts as a fifth letter in a DNA library, provides a series of high-affinity DNA aptamers with versatile binding specificities and activities to cancer cells. These Ds-containing DNA aptamers were generated by a method called cell-ExSELEX to target three breast cancer cell lines: MCF7, MDA-MB-231, and T-47D. Aptamer 14A-MCF7, which targets MCF7 cells, specifically binds to MCF7 cells, but not other cancer cell lines. Aptamer 07-MB231, which targets MDA-MB-231 cells, binds to a series of metastatic bone and lung cancer cell lines. Aptamer 05-MB231 targets MDA-MB-231 cells, but it also binds to all of the cancer and leukemia cell lines that we examined. None of these aptamers bind to normal cell lines, such as MCF10A and HUVEC. In addition, aptamers 14A-MCF7 and 05-MB231 are internalized within the cancer cells, and aptamer 05-MB231 possesses anti-proliferative properties against most cancer cell lines that we examined. These aptamers and the generation method are broadly applicable to cancer cell imaging, biomarker discovery, cancer cell profiling, anti-cancer therapies, and drug delivery systems.

INTRODUCTION

The creation of unnatural base pairs (UB pairs or UBPs) that exhibit high fidelity in replication as a third base pair has led to novel biotechnologies. The UBP technologies provide new material production platforms, such as semisynthetic organisms that generate novel proteins containing unnatural amino acids[1,2] and high-affinity DNA aptamers that specifically bind to target proteins and cells.[3,4] In particular, hydrophobic UBs as a fifth letter greatly increase the chemical and physical diversities of functional DNA molecules.[3,5] To pursue further UBP technology applications toward diagnostics and therapeutics, we have developed a method, cell-ExSELEX, to generate high-affinity UB-containing DNA aptamers that bind to cancer cells.

DNA aptamers are single-stranded oligonucleotides that specifically bind to target molecules and materials of interest. They are isolated from a nucleic acid library containing ~10^{15} different sequence species by an evolutionary engineering method called SELEX (Systematic Evolution of Ligands by EXponential enrichment).[6,7] Once their sequences are determined by the SELEX procedure, chemical synthesis enables their large-scale preparation and modification for many purposes. In addition, cell-SELEX, a variation of SELEX, can also target whole living cells and has yielded many aptamers that bind to specific cells.[8–13] For practical diagnostic and therapeutic uses, some critical issues of DNA aptamers include their insufficient affinity to targets and instability against nucleases. Thus, many modification and modified aptamer generation methods have been reported to tackle these issues.[14–22]

Genetic alphabet expansion by creating UBPs can enlarge the chemical and structural diversities of DNA aptamers in SELEX.[3–5,23] We developed a SELEX method using genetic alphabet expansion (ExSELEX; genetic alphabet Expansion for SELEX) (Figure 1) with our hydrophobic UBP between Ds (7-(2-thienyl)imidazo[4,5-*b*]pyridine) and Px (2-nitro-4-propynylpyrrole) (Figure 1A).[24,25] In ExSELEX, DNA libraries contain the highly hydrophobic Ds bases as a fifth letter. Even if the libraries contain UBPs, they can be faithfully replicated and amplified as duplexes by PCR in the presence of the triphosphate substrates of Ds and Px, as well as the natural base substrates. Using ExSELEX, we generated several high-affinity Ds-containing DNA (Ds-DNA) aptamers that target proteins.[3,5] The highly hydrophobic Ds bases in the generated aptamers significantly reinforce their affinities to the hydrophobic regions of target proteins. Although Benner's group applied their hydrogen-bonded UBP, Z-P, to cell-SELEX, the affinities of their aptamers to the targets were moderate because of their limited hydrophobicity.[4,26,27]

Here, to explore the versatility and further availability of Ds-DNA aptamers, we applied ExSELEX to Ds-DNA aptamer generation by targeting three representative breast cancer cell lines: MCF7, MDA-MB-231, and T-47D. From each cell-ExSELEX used with the respective

Received 17 July 2018; accepted 15 November 2018;
https://doi.org/10.1016/j.omtn.2018.11.011.

Correspondence: Ichiro Hirao, Institute of Bioengineering and Nanotechnology, 31 Biopolis Way, The Nanos, #07-01, Singapore 138669, Singapore.
E-mail: ichiro@ibn.a-star.edu.sg

This is an open access article under the CC BY license (http://creativecommons.org/licenses/by/4.0/).

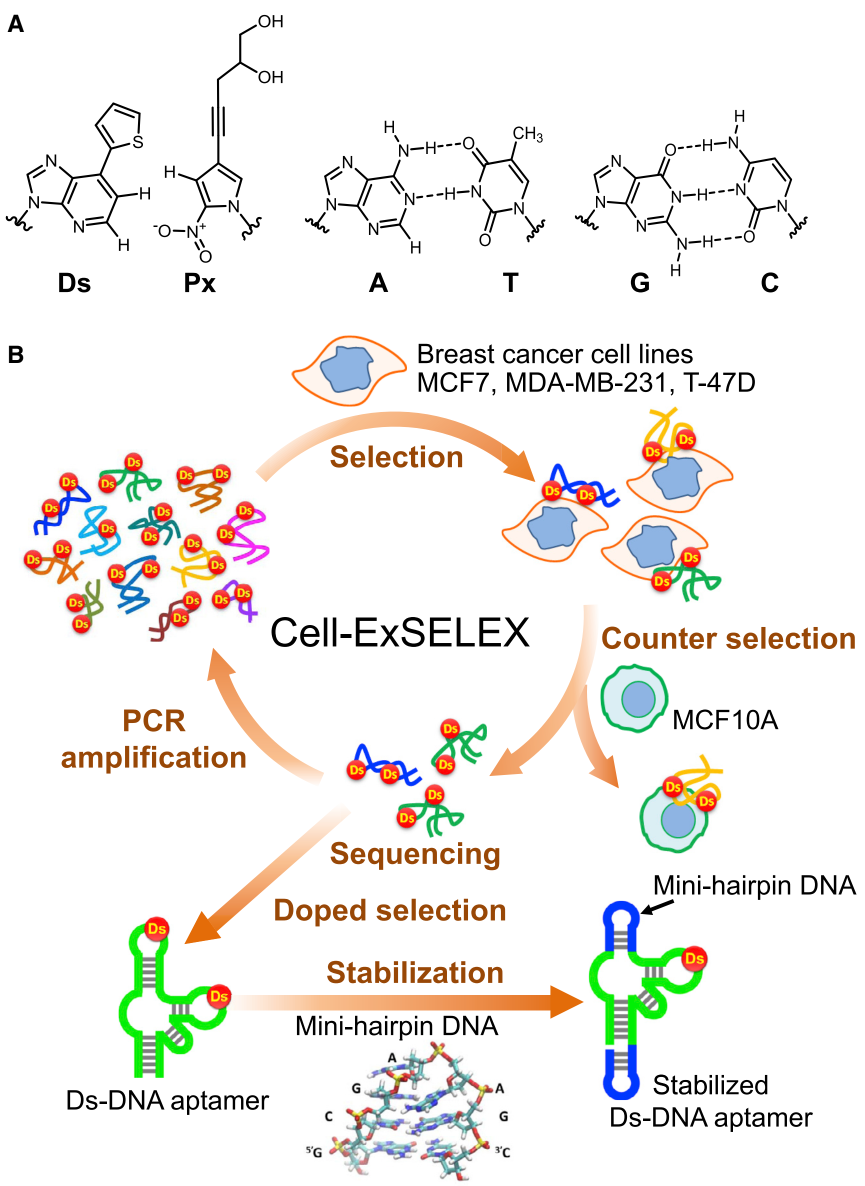

Figure 1. Stabilized Ds-DNA Aptamer Generation Targeting Breast Cancer Cell Lines with ExSELEX and Mini-Hairpin DNA

(A) A hydrophobic unnatural Ds-Px base pair and the natural A-T and G-C base pairs. (B) Schematic representation of ExSELEX targeting cells (cell-ExSELEX), using a Ds-DNA library to generate Ds-DNA aptamers. The obtained aptamer candidates, identified by deep sequencing from the enriched libraries, are each subjected to a doped selection procedure to optimize the aptamer sequence and to predict its secondary structure. Based on that information, the Ds-DNA aptamer is stabilized by the introduction of a mini-hairpin DNA structure with extraordinary thermal stability and nuclease resistance.

cell line, we obtained several Ds-DNA aptamers with unique specificities, from target cell specific to broadly cancer cell specific, and/or with some biological activities, such as internalization within the target cells and anti-proliferative activity. These results indicate the high potential of the Ds-DNA aptamers obtained by cell-ExSELEX for a wide range of applications toward cancer diagnostics and therapeutics and also suggest that genetic alphabet expansion increases the diversification of DNA aptamer generation and functionalization.

RESULTS

Cell-ExSELEX Targeting Breast Cancer Cells

In this cell-ExSELEX (Figure 1B), we used a Ds-predetermined DNA library,[3,28] which was a mixture of 24 different sub-libraries containing A, G, C, T, and Ds bases (Table S1). Each sub-library contained two Ds bases at different defined positions in a 42-natural-base random region and a unique three-natural-base barcode sequence. Our previous results revealed that one or two Ds bases in the library are sufficient for the generation of aptamers with more than 100-fold higher affinity than conventional aptamers obtained from a library consisting of only natural bases.[3,5] No aggregation or difficult solubility of the Ds-containing DNA library in a buffer was observed.

We chose three breast cancer cell lines—MCF7, MDA-MB-231, and T-47D—as the targets and started cell-ExSELEX for each target using the Ds-predetermined DNA library with 0.6 × 10^{15} or 1.2 × 10^{15} different sequence combinations. After repetitive rounds of cell-ExSELEX, each DNA sequence in the enriched libraries was determined with an Ion PGM sequencing system (Life Technologies). In the sequencing system, the Ds bases in each DNA were replaced with the natural bases by replacement PCR, and the Ds positions in each DNA sequence were identified by the barcode embedded in each sub-library.

The cell-ExSELEX conditions are summarized in Tables S2–S4. We used a counter-selection to remove the aptamer species that bound to normal cells and used the human mammary epithelial cell line MCF10A as a negative control, before or after the positive selection step from the second or third round. After seven rounds of selection, we monitored the binding abilities of the libraries isolated from each round by flow cytometry, using the fluorescently labeled library and target cells (Figure S1). We observed that seven rounds of selection sufficiently enriched the binding species in each library.

The enriched sequences of each library were determined by standard deep sequencing with the Ion PGM system, followed by the barcode identification of the Ds positions.[3] The sequences with high copy numbers (more than 2%) in each cell-ExSELEX targeting MCF7, MDA-MB-231, and T-47D cells are listed in Table S5. Based on the binding ability of each candidate sequence to the target cells (data not shown), we chose six sequences for further studies: 14A (14A-MCF7) and 08B (08B-MCF7), obtained from cell-ExSELEX, targeting MCF7 cells; 05 (05-MB231), 07 (07-MB231), and 23 (23-MB231), targeting MDA-MB-231 cells; and 03 (03-T47D), targeting T-47D cells.

To optimize each aptamer sequence and to predict its secondary structure,[3] we performed a second cell-ExSELEX, a doped selection, using chemically synthesized doped libraries based on each aptamer candidate sequence (Tables S2–S4). Each doped library contained 55% of the initial natural bases of each selected sequence position, and 15% each of the other three natural bases, but the Ds positions were fixed. After four rounds of selection, we amplified the enriched libraries by replacement PCR and sequenced them. The observed covariations in the obtained sequence data suggested base pair formation in each aptamer (08B-MCF7, 14A-MCF7, 07-MB231, 05-MB231, 23-MB231, and 03-T47D) and facilitated their secondary structure prediction[3,29] (Figures S2–S13) and optimization (08B-MCF7(DsDs51), 14A-MCF7(DsDs53), 07-MB231(DsDs43), 05-MB231(DsDs49), 23-MB231(DsDs49), and 03-T47D(DsDs33); Figure 2). From the sequence data, we observed that the second diversification by the doped cell-ExSELEX resulted in the further evolution and optimization of each aptamer sequence.

High Binding Affinities of Each Selected Aptamer and Its Variants to Target Cell Lines

We chemically synthesized the selected aptamers—08B-MCF7(DsDs51), 14A-MCF7(DsDs53), 07-MB231(DsDs43), 05-MB231(DsDs49), 23-MB231(DsDs49), and 03-T47D(DsDs33)—and their variants to assess each aptamer's binding affinity. First, we replaced one or two Ds bases in each aptamer with adenine to identify which Ds bases are essential (Figures 2 and S8–S13) for binding. Second, we introduced an extraordinarily stable mini-hairpin DNA sequence, 5′-CGCGAAGCG-3′,[30–32] at the 3′ terminus of each aptamer[5,33,34] to resist nuclease digestion. For 08B-MCF7, we synthesized an additional variant, 08B-MCF7(Ds54mh2), by replacing one of the internal stem-loops with the mini-hairpin sequence. For 14A-MCF7 and 05-MB231, we also synthesized other variants, in which the A-T pairs in the terminal stem were replaced with G-C pairs. These aptamers and variants were labeled with Alexa 488 at their 5′ termini or at the internal A in the mini-hairpin sequence, which was replaced with the amino-modifier C6-dT for labeling.

We assessed the binding affinities of each aptamer and its variants by flow cytometry, using its respective target breast cancer cell line (Figure S14). We used the known aptamer, AS1411 (26-mer, AS1411-26), and its G → C variant (26-mer, Cont26) as positive and negative controls, respectively. AS1411 is a conventional DNA aptamer consisting of only G and T bases. It forms a G-quadruplex and binds to nucleolin, a unique biomarker found predominantly on tumor cell surfaces, and it has been tested as an anti-cancer drug.[35]

All of the aptamers originally contained two Ds bases, which existed within the initial Ds-predetermined sub-libraries. From the examination of Ds → A variants, one or two Ds bases strongly affected the target binding of each aptamer. For example, for 08B-MCF7 (Figures S8 and S14), the aptamers of DsDs51, ADs51, DsDs60mh, and Ds54mh2 efficiently bound to MCF7 cells, but DsA51 and AA51 did not bind. Thus, the Ds base at position 32 is essential for the binding, and the stem-loop structure containing another Ds base at position 18 can be replaced with the mini-hairpin DNA (08B-MCF7(Ds54mh2)).[5,33] As for 14A-MCF7 (Figures S9 and S14), the aptamer and its variants strongly bound to MCF7 cells, except for the Ds → A mutants, especially DsA53 and AA53. Thus, both Ds bases are important for binding, and, in particular, the Ds base at position 28 is irreplaceable. The binding of DsDs53op (AG-bulge → TT-bulge; Figures S9 and S14) indicated that the two bulged bases at positions 40 and 41 are not essential. Therefore, the terminal stem can be trimmed to three to six G-C pairs and one G-T pair with the mini-hairpin DNA (DsDs44mh–DsDs38mh).

We confirmed the high affinities of the aptamers 08B-MCF7, 14A-MCF7, 05-MB231, and 07-MB231 to their target cell lines (K_D = 1–3 nM) by flow cytometry (Figures 3A and S15). The K_D values of the optimized and stabilized aptamers with mini-hairpin DNA sequences—08B-MCF7(Ds54mh2), 14A-MCF7(DsDs42mh), 05-MB231(Ds45mh), 07-MB231(DsDs52mh), 23-MB231(DsDs58mh), and 03-T47D(DsDs42mh)—that target each breast cancer cell line are shown in Figure 3A. For the following experiments, we used these optimized and stabilized aptamers.

Fluorescent microscopic imaging patterns also supported the binding of the Alexa 488-labeled Ds-DNA aptamers, as well as AS1411-26 as a control, to each target cell line (Figures S16 and S17). Bright Alexa 488 fluorescence was observed on each target cell when incubated with each aptamer, indicating that all of the Ds-DNA aptamers bound to each target cell. However, no fluorescence was observed on the normal MCF10A cells when incubated with the Ds-DNA aptamers. In contrast, AS1411-26 was less selective and bound to both MCF7 and MCF10A cells (Figure S16).

Cellular Internalization of Aptamers 14A-MCF7 and 05-MB231

An analysis by confocal laser scanning microscopy confirmed the internalization of the Alexa 488-labeled aptamers 14A-MCF7(DsDs42mh) (Figures 3B and 3C) and 05-MB231(Ds45mh) (Figure S18) within the cancer cell. The Alexa 488 images were compared to those obtained using DAPI and LysoTracker, which target the nucleus and lysosomes, respectively (Figures 3B and S18). AS1411 was also internalized within the cell, as reported,[35] and the bright parts of the Alexa 488 fluorescence image with AS1411-26 were similar to those of the LysoTracker image. The images obtained using aptamer 14A-MCF7(DsDs42mh) also showed the same tendency, which became more pronounced by increasing the incubation

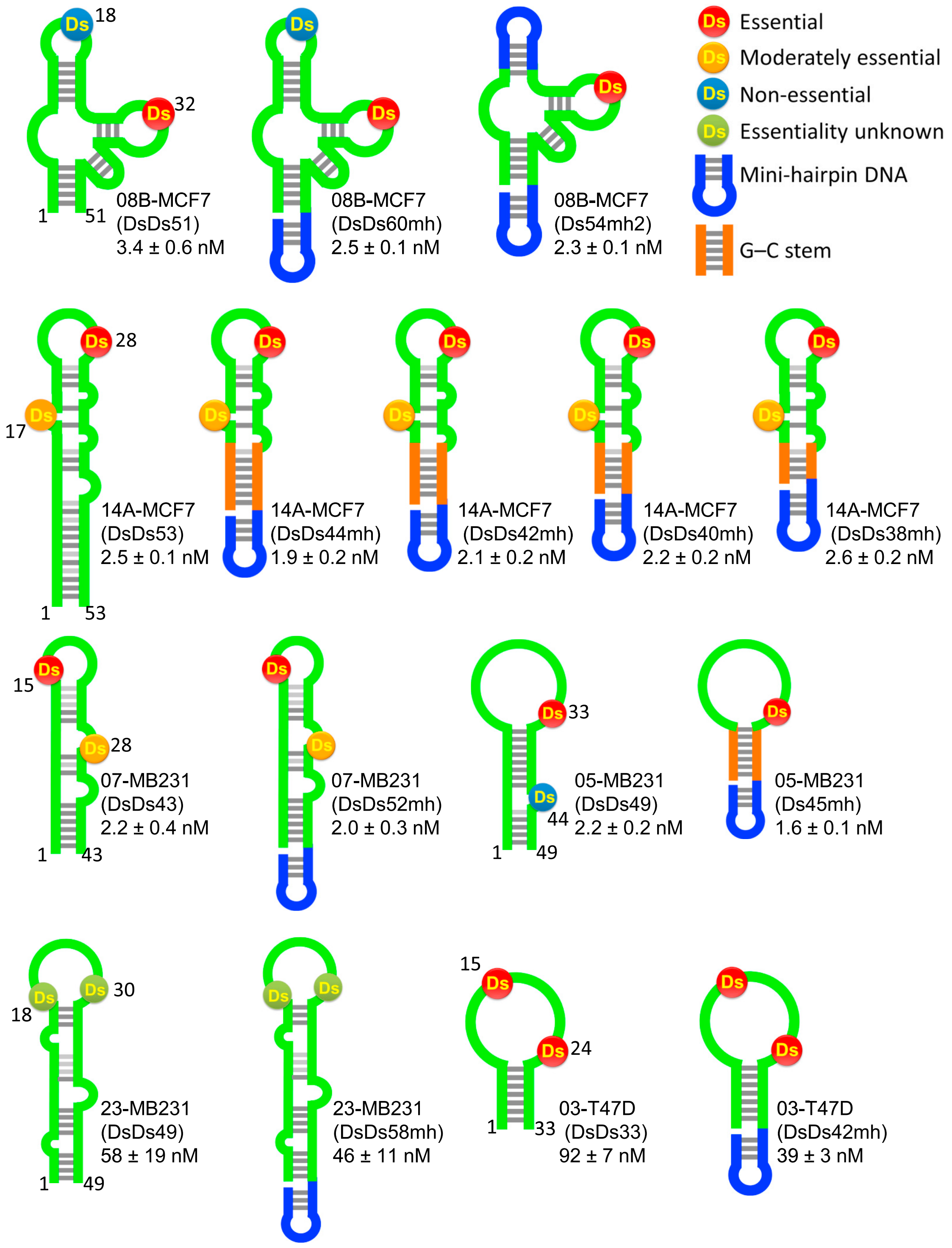

Figure 2. Stabilization of the Ds-DNA Aptamers with Mini-Hairpin DNAs Does Not Perturb Their Binding Abilities to the Target Cell Lines

Predicted secondary structures of the six obtained Ds-DNA aptamers and their stabilized variants are shown, along with their K_D values to each corresponding target cell line (MCF7, MDA-MB-231, and T-47D). Some parts containing non-essential Ds bases (Ds18 in 08-MCF7 and Ds44 in 05-MB231) and double-stranded regions are compatible with a mini-hairpin DNA and G-C stem structures, without any loss of each aptamer's original binding ability.

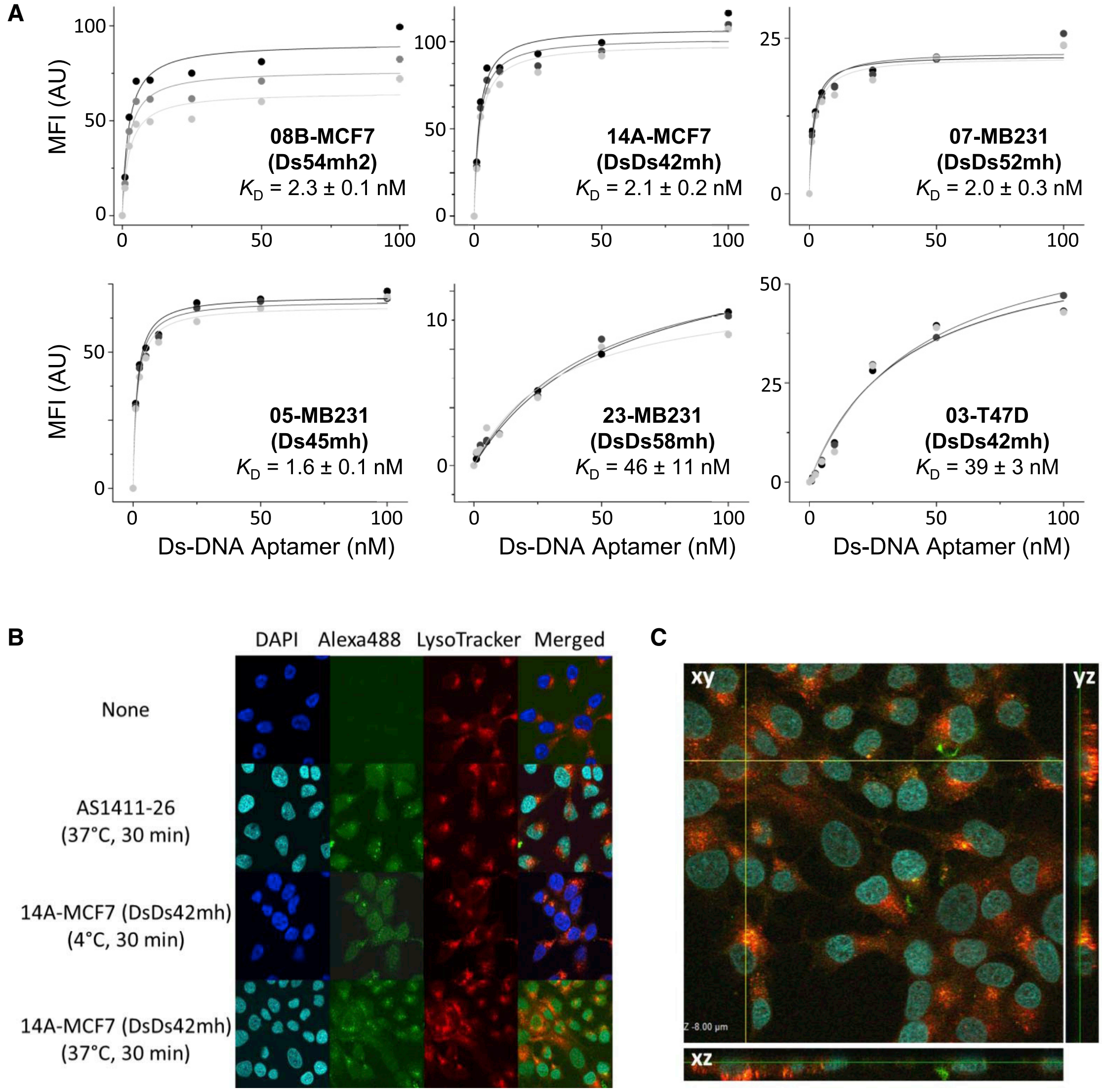

Figure 3. Binding of the Ds-DNA Aptamers to the Target Cells

(A) The dissociation constant (K_D) values of the aptamers to the target cells determined from flow cytometry analyses. Geometric mean fluorescence intensities (MFIs) are plotted against the aptamer concentration used for binding to each target cell line. The K_D value was calculated from each dataset (three times), and the averaged values are shown with their SDs. (B) Internalization of the aptamers into MCF7 cells, as detected by confocal microscopy. MCF7 cells were incubated in the presence or absence of 250 nM Alexa 488-labeled AS1411-26 or 14A-MCF7(DsDs42mh) for 30 min at either 4°C or 37°C, after the incubation with LysoTracker to detect acidic organelles inside the cell. In the merged images, the DAPI (nuclei), Alexa 488 (aptamer), and LysoTracker signal images were overlaid. (C) Orthogonal view with the x-y, x-z, and y-z sections of the merged images obtained by confocal microscopy, showing the clear internalization of 14A-MCF7 within MCF7 cells.

temperature from 4°C to 37°C. In the 3D image of the merged picture for 14A-MCF7(DsDs42mh), the Alexa 488 images resembled the LysoTracker images (Figure 3C), indicating that the aptamer 14A-MCF7(DsDs42mh) was internalized within MCF7 via endocytosis.

Versatile Binding Specificities of Ds-DNA Aptamers in Cancer Cell Imaging

We tested the binding specificity of each Alexa 488-labeled aptamer to the three breast cancer cell line targets and other cancer and leukemia cell lines (MIA PaCa-2, Panc-1, PC-3, HeLa, A549, and CCRF-CEM), as well as normal cell lines (MCF10A and HUVEC), with fluorescent microscopic imaging (Figure 4). Aptamer 14A-MCF7(DsDs42mh) specifically bound to MCF7 cells. In contrast, 07-MB231(DsDs52mh) bound to not only MDA-MB-231 but also PC-3 and A549 cells, but IT did not bind to the other breast cancer cell lines, MCF7 and T-47D. Interestingly, 05-MB231(Ds45mh) and 03-T47D(DsDs42mh) bound to all of the cancer and leukemia cell lines that we examined. We confirmed that aptamer 05-MB231(Ds45mh) also bound tightly to

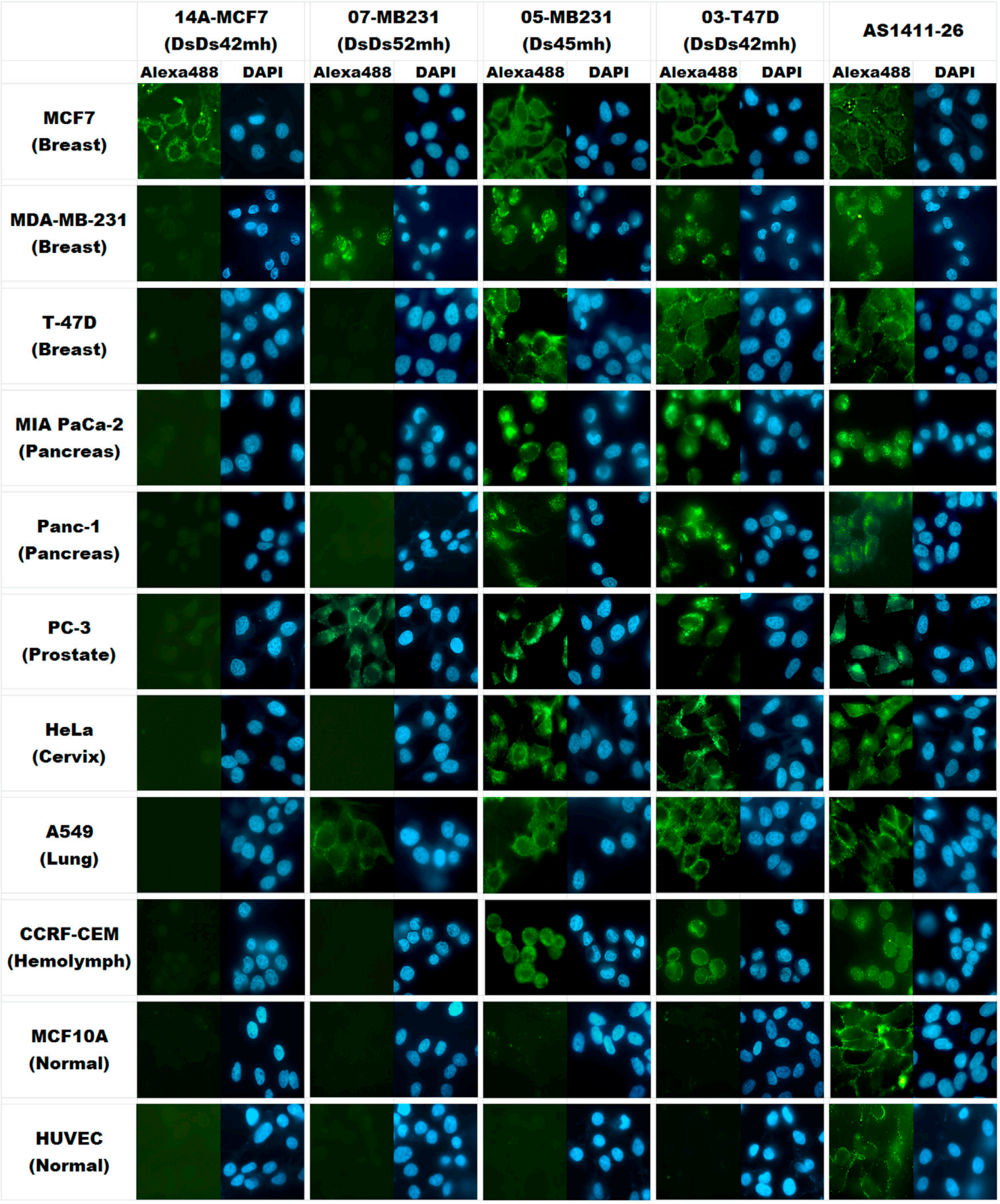

Figure 4. Cancer Cell Imaging by the Ds-DNA Aptamers

A variety of cancer cell lines (MCF7, MDA-MB-231, T-47D, MIA PaCa-2, PC-3, HeLa, A549, and CCRF-CEM), as well as normal cell lines (MCF10A and HUVEC), were incubated with 250 nM each of Alexa 488-labeled Ds-DNA aptamer and AS1411-26 (as a control) at 4°C for 30 min and then analyzed by fluorescent microscopy.

T-47D cells, as well as MDA-MB-231 cells, by flow cytometry (Figure S19). All of the Ds-DNA aptamers examined here scarcely bound to the normal cell lines, MCF10A and HUVEC. In contrast, AS1411-26 bound to all of the cancer cell lines, but it also bound slightly to the normal cell lines. These results suggest that the targets of 14A-MCF7(DsDs42mh), 07-MB231(DsDs52mh), 05-MB231(Ds45mh), and AS1411-26 differ from one another.

We confirmed the binding of 14A-MCF7(DsDs38mh), 07-MB231(DsDs52mh), and 05-MB231(Ds45mh) to each target cell by

flow cytometry at 4°C and 37°C (Figure S20). Although the cell-ExSELEX performed at 4°C, these aptamers retained their binding ability at 37°C. Interestingly, 05-MB231(Ds45mh) and AS1411-26 bind more efficiently at 37°C, compared to their binding at 4°C.

We also examined the stabilities of 14A-MCF7(DsDs38mh), 07-MB231(DsDs52mh), and 05-MB231(Ds45mh) in human serum at 37°C, in comparison with AS1411-26 (Figure S21). AS1411 forms a G-quadruplex structure, and it is more stable than a non-structured DNA (25-mer). Most of the 25-mer DNA was digested by nucleases in human serum within a 24-hr incubation at 37°C, while ~30% of AS1411-26 still remained intact. In contrast to AS1411-26, the mini-hairpin addition to our aptamers was more effective in resisting nuclease digestion, as more than 40% of the aptamers, 05-MB231(Ds45mh), 07-MB231(DsDs52mh), and 14A-MCF7(DsDs38mh), survived after a 24-hr incubation in human serum.

We performed competition experiments between high-affinity aptamer pairs to determine whether each aptamer binds to different targets from the others (Figures S22 and S23). Aptamers 14A-MCF7(DsDs42mh) and 08B-MCF7(Ds54mh2) competed with each other for binding to MCF7 cells, but no competition was observed among 14A-MCF7(DsDs42mh), 05-MB231(Ds45mh), and 07-MB231(DsDs52mh). These results suggest that 14A-MCF7(DsDs42mh), 05-MB231(Ds45mh), and 07-MB231(DsDs52mh) bind to different antigens, as expected. We also confirmed that 05-MB231(Ds45mh) binding did not compete with AS1411-26 binding to MDA-MB-231 (Figure S24), and 05-MB231(Ds45mh) was internalized not only within the target MDA-MB-231 but also within MCF7 and T-47D cells (Figure S18).

Unique Binding Specificities of Ds-DNA Aptamers Allow Cancer Cell Profiling

For further exploration of the specificity of each Ds-DNA aptamer to each cancer cell line, the binding properties of each aptamer-cell combination were analyzed by flow cytometry assays using six Ds-DNA aptamers (14A-MCF7(DsDs42mh), 08B-MCF7(Ds54mh2), 07-MB231(DsDs52mh), 23-MB231(DsDs58mh), 05-MB231(Ds45mh), and 03-T47D(DsDs42mh) (hereinafter, we omit the indications of the aptamer name in parentheses), AS1411-26, and Cont26, with 15 cancer cell lines and two normal cell lines. The binding pattern panels of each DNA sample to each cell line are summarized in Figure S25.

These cancer cell panels can be classified into four categories, based on each aptamer's binding tendency (Figure 5): category 1, which includes MDA-MB-231, PC-3, and A549 cells; category 2, which includes MDA-MB-453, CCRF-CEM, T-47D, and KG-1 cells; category 3, which includes MIA PaCa-2, OVCAR-3, HCT116, HeLa, Panc-1, and PLC/PRF/5 cells; and category 4, which includes other cells (MCF7 and MKN45). The aptamer 07-MB231 bound to the cells in category 1 specifically, although 05-MB231, 03-T47D, and AS1411-26 also bound to those cells. The cells in category 2 bound to 05-MB231, 03-T47D, and AS1411-26, and the fluorescent intensities were 05-MB231 > > 03-T47D > AS1411-26. The cells in category 3 also bound to 05-MB231, 03-T47D, and AS1411-26, but the fluorescent intensities were 05-MB231 > 03-T47D ≈ AS1411-26.

We found that categories 1–3 each have a common aspect. The category-1 cell lines are metastatic cancers of the bones (MDA-MB-231 and PC-3) and lungs (A549). The category-2 cell lines, except for T-47D, are low cell adhesive. The category-3 cell lines are related to gastrointestinal or gynecological cancers of endocrine organs. Thus, a new type of cancer profiling might be possible, with classifications using several Ds-DNA aptamers that have different binding specificities to cancer cell lines.

Furthermore, the differences in the fluorescent intensities of each category might reflect the unique expression level of each aptamer target on the cell, when the aptamer's affinity is sufficiently high. For example, the fluorescence intensity of the aptamer 05-MB231 binding to T-47D cells was much higher than that to MDA-MB-231 cells (Figure S19). Thus, high-affinity Ds-DNA aptamers could bind to and profile cancer cells based on the quantitative information of the antigens.

Anti-cancer Function of the Ds-DNA Aptamer 05-MB231

The cytostatic activities of five Ds-DNA aptamers, 08B-MCF7, 05-MB231, 07-MB231, 23-MB231, and 03-T47D, were examined by a WST-8 assay using several cancer cell lines. The activities were compared to those of AS1411-26 and Cont26, as a positive and a negative control, respectively. In addition, other anti-cancer drugs, 5-fluorodeoxyuridine (FUdR) and Paclitaxel (PTX), were also used as positive controls (Figures 6, S26, and S27). Among the five Ds-DNA aptamers, only 05-MB231 showed clear cytostatic activity against several cancer cell lines—MDA-MB-231, MCF7, A549, KG-1, T-47D, MIA PaCa-2, HCT116, and PC-3—but not the normal cell lines, MCF10A and HUVEC. We also confirmed that 05-MB231's cytostatic activity largely depends on the Ds base within 05-MB231, since the variant of 05-MB231 where the Ds base was replaced with the natural base completely lost its activity (data not shown), and the binding affinity of the variant was largely reduced (Figure S20). In contrast, AS1411-26, as well as FUdR and PTX, showed slight cytostatic activities against MCF10A and HUVEC cells.

The anti-cancer activity of 05-MB231 does not result from the Ds nucleoside or nucleotide itself. The Ds nucleoside exhibits slight toxicity against both cancer and normal cell lines (IC_{50} = 120 ± 30 μM against CCRF-CEM, 56 ± 39 μM against HT-1080, and 190 ± 140 μM against WI-38).[36] However, these slight toxicities might not affect the anti-cancer activity of 05-MB231, containing only one Ds base, within the aptamer concentration range (1–10 μM) for the assay. Further detailed examinations of the intrinsic toxicity of the aptamer are in progress. At present, we have found that the Ds nucleoside exhibited no mutagenic effects on *Salmonella typhimurium* (TA98, TA100, TA1535, and TA1737) and *Escherichia coli* (WP2uvrA) in an Ames test (data not shown).

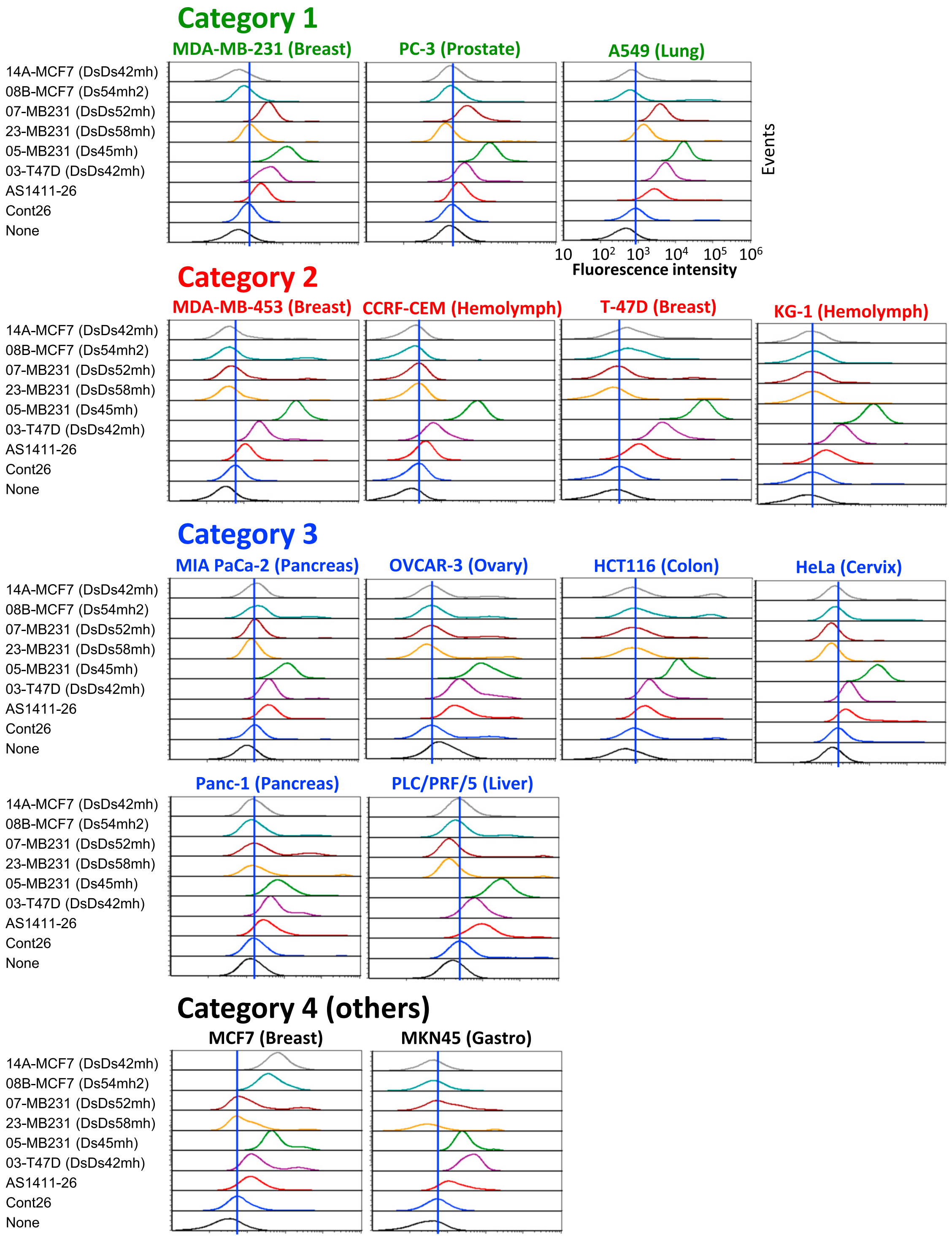

Figure 5. Cancer Cell Profiling Based on Binding Patterns of a Series of Ds-DNA Aptamers and AS1411-26

Fifteen cancer cell lines were incubated with 250 nM of Alexa488-labeled Ds-DNA aptamer, as well as of AS1411-26 and Cont26 as controls, at 4°C for 30 min, followed by a flow cytometry analysis. Based on the patterns of the fluorescent signals, the cancer cell lines were classified into four categories (the cell lines indicated in green, red, blue, and black). The blue straight line in each panel corresponds to the median fluorescence intensity for Cont26.

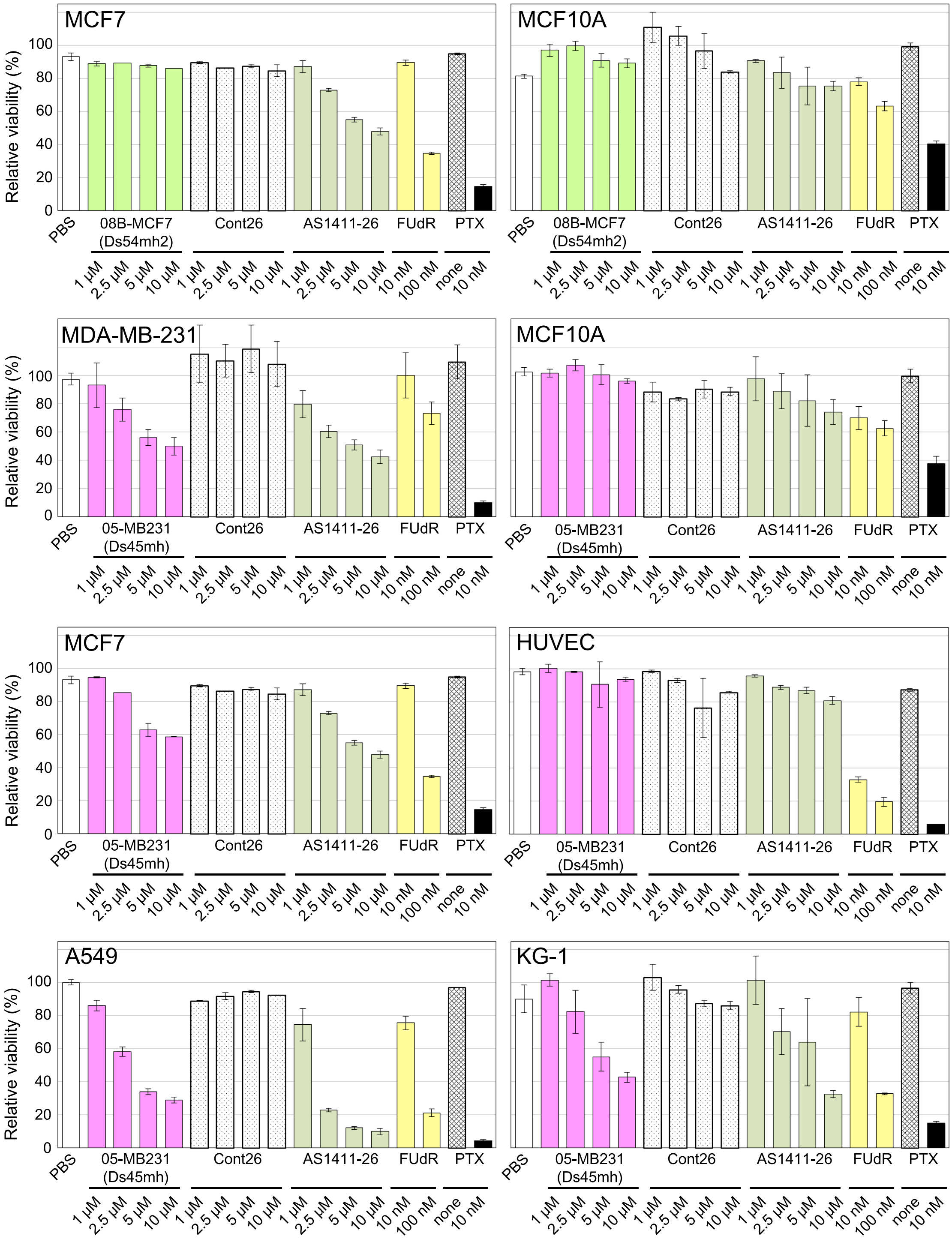

Figure 6. Anti-proliferative Effects of 05-MB231 on Several Cancer Cell Lines

The cells were incubated for 96 hr with 08B-MCF7 or 05-MB231, as well as Cont26, AS1411-26, and the anti-cancer drugs FUdR (5-fluorodeoxyuridine) and PTX (Paclitaxel), and the relative viability was determined with a WST-8 assay. The assays were performed independently at least twice, and then the relative viabilities were averaged. The error bars represent SD.

DISCUSSION

Through cell-ExSELEX targeting breast cancer cell lines, we have demonstrated the high potential of Ds-DNA aptamers and how their generation method, cell-ExSELEX, which introduces hydrophobic unnatural components to DNA aptamers, significantly augments the versatility and functionality of biopolymers. We generated several unique Ds-DNA aptamers by cell-ExSELEX, targeting three representative breast cancer cell lines. The affinities of most Ds-DNA aptamers are very high, as compared to those of the natural-base aptamers obtained by other cell-SELEX methods. These Ds-DNA aptamers can be used for cancer detection, cancer cell imaging, drug delivery systems, and anti-cancer drugs. The anti-cancer activity of 05-MB231 could be augmented by conjugating the aptamer to various anti-cancer drugs.

We observed high diversity in the specificities and biological activities of the Ds-DNA aptamers targeting cancer cells. Aptamer 14A-MCF7 strictly binds only to its target cell. Another aptamer, 07-MB231, binds to a series of metastatic bone and lung cancer cells. In contrast, aptamer 05-MB231 binds to all of the cancer cells that we tested and inhibits their proliferation. In addition, we also confirmed that 14A-MCF7 and 05-MB231 are internalized within the bound cells. Generating an assortment of Ds-DNA aptamers that target a variety of cancer cell lines could increase the chance of discovering new cancer-specific antigens, or neoantigens.

Although we still lack information about the actual target antigens of each Ds-DNA aptamer, the target identification by each aptamer provides valuable information for cancer characterization and new cancer biomarker discovery. In particular, the target of 05-MB231 is quite interesting, as 05-MB231 binds to a wide variety of cancer cell lines, as does the well-known conventional aptamer, AS1411. However, we confirmed that the target of 05-MB231 is not nucleolin, which is the AS1411 target (data not shown). An analysis of the mechanisms by which 05-MB231 exhibits the cytostatic activity might lead to the development of a better anti-cancer drug and synergistic combinations with other types of anti-cancer drugs.

Based on the binding and profiling data and the competition experiments of each aptamer, the target antigen for 14A-MCF7 and 08B-MCF7 would be the same, while the antigens of 14A-MCF7, 05-MB231, and 07-MB231 would be different. It is notable that the expression level of each antigen could be predicted from the binding amount (fluorescent intensity) of each labeled aptamer when high-affinity aptamers are used, as in the case of 05-MB231 (Figure S19).

As shown here, ExSELEX could provide a new cancer-profiling method, using a series of Ds-DNA aptamers for personalized medicine to select appropriate anti-cancer drugs.[37–40] Currently, many types of cell-SELEX methods, including another genetic alphabet expansion method, have been developed. Thus, cancer profiling using aptamers could advance further, by combining the Ds-DNA aptamers with other UB-aptamers and conventional aptamers with different specificities and affinities. Benner and Tan's team reported another UB-DNA aptamer that targets MDA-MB-231 cells with moderate affinity (K_D = 30 nM), generated by cell-SELEX using their UBP, Z-P.[4] Yang's team generated natural-base-DNA aptamers (K_D = 2.6–108 nM) that target MDA-MB-231 cells by conventional cell-SELEX, and these aptamers specifically bound to MDA-MB-231 and T-47D cells.[41] Mayer's team reported their natural-base-DNA aptamers that target MCF7 cells, which also have broad specificity to other cancer cells, such as A549 and THP1.[42] Another aptamer (K_D = 5.9–138.2 nM) that targets the metastatic colorectal carcinoma LoVo bound to only the target cells.[43] However, for the precise and valid quantitative analysis of the biomarkers on the surface of cancer cells, a series of high-affinity DNA aptamers (K_D = 1–5 nM measured by flow cytometry) will be required.

The cell-ExSELEX method could provide valuable information for cancer research and pharmaceutical applications toward individualized cancer medicine. In addition, cell-ExSELEX can be used to target other types of cells, including stem cells and induced pluripotent stem cells (iPSCs).

MATERIALS AND METHODS

Nucleotides and Oligonucleotides

The unnatural nucleoside triphosphates, dDsTP and dPxTP, and the Ds phosphoramidite were synthesized as described previously.[44] DNA fragments with Ds bases were either chemically synthesized with oligonucleotide synthesizers, nS-8 (GeneDesign), and an H8 DNA synthesizer (K&A Laborgeraete), by using phosphoramidite reagents for the natural and Ds bases (Glen Research), or purchased from GeneDesign. The chemically synthesized DNA fragments were purified by denaturing PAGE.

DNA Library

The DNA library used for cell-ExSELEX was prepared by mixing 24 different single-stranded DNA sub-libraries (93-mer; Table S1). Each sub-library consisted of a 5′ primer sequence, defined three-base natural sequences as bar codes corresponding to the two Ds positions, a 42-nt randomized sequence containing two Ds bases at predetermined positions, and a 3′ primer sequence.

Cell Lines

The human breast cancer cell line MCF7 (RCB1904) was provided by the RIKEN BioResource Center (BRC), through the National Bio-Resource Project of the MEXT, Tsukuba, Japan. The MCF7 cell line was maintained in Minimum Essential Media (Life Technologies) supplemented with 10% fetal bovine serum (FBS; Nichirei Biosciences), non-essential amino acids, and 1 mM sodium pyruvate (Life Technologies). The MCF10A cell line (CRL-10317), used in the counter-selection in cell-ExSELEX, was purchased from the American Tissue Culture Collection (ATCC). The base medium (MEBM, Lonza) for MCF10A was prepared with the additives provided in an MEGM kit (Lonza). Other human breast cancer cell lines, T-47D (ATCC, #HTB-133) and MDA-MB-231 (ATCC, #HTB-26), were maintained according to the supplier's instructions. For the specificity profiling of the obtained aptamers, other types of

cell lines—MDA-MB-453 (BRC, #RCB1192), HCT-116 (BRC, #RCB2979), A549 (BRC, #RCB0098), MKN45 (BRC, #RCB1001), NIH:OVCAR-3 (BRC, #RCB2135), PC-3 (ATCC, #CRL-1435), MIA PaCa-2 (from the Cell Resource Center for Biomedical Research, Institute of Development, Aging and Cancer (IDAC), Tohoku University, #TKG0227), Panc-1 (IDAC, #TKG0606), PLC/PRF/5 (from the Japanese Collection of Research Bioresources Cell Bank, #JCRB0406), HeLa (BRC, #RCB0007), KG-1 (BRC, #RCB1166), CCRF-CEM (IDAC, #I-4924), and HUVEC (Lonza, #CC-2519)—were also cultured according to the suppliers' instructions and used for evaluations of the aptamers' binding. These cell lines were cultured in a 37°C incubator under a 5% CO_2 atmosphere.

Cell-ExSELEX

The initial single-stranded DNA library (1 or 2 nmol), diluted in Dulbecco's PBS (D-PBS (−)), was denatured by heating at 95°C for 5 min and then kept at room temperature for 20 min. The folded DNA library solution was diluted in binding buffer (final composition: 4.5 g/L glucose, 5 mM $MgCl_2$, 0.1 mg/mL tRNA, and 1 mg/mL BSA, in D-PBS (−)) and incubated with approximately 5×10^6 target cells in a culture dish at 4°C for 60 min with gentle agitation. Unbound DNA species were removed by washing with ice-cold washing buffer (4.5 g/L glucose and 5 mM $MgCl_2$ in D-PBS (−)) and then 0.5 mL water was added to the culture dish. To collect the cell-bound single-stranded DNA (ssDNA) species, the cells were scraped off from the culture dish with a cell scraper. The recovered cell-DNA solution was then heated at 95°C for 10 min, followed by centrifugation at 14,000 rpm for 20 min to pellet the cell debris. The supernatant, which contained the eluted DNA, was then subjected to PCR amplification, as described previously.[3] The PCR conditions were 15 s at 94°C and 3.5 min at 65°C per cycle, after polymerase activation for 2 min at 94°C. The amplified DNA library was purified by denaturing gel electrophoresis to obtain the single-stranded Ds-DNA library for the next selection round. For the second round, the DNA library was incubated with a counter-cell line, MCF10A, at 4°C for 60 min in a culture dish, and then the supernatant was collected and incubated with the target cells. The cell-ExSELEX conditions are summarized in Tables S2–S4.

Flow Cytometry Analysis

The enrichment process of the DNA library during the cell-ExSELEX, as well as each aptamer's binding capability, was analyzed by flow cytometry. The cultured cells were dissociated by an incubation with 0.2% EDTA, after washing with D-PBS (−). After washing with ice-cold washing buffer twice, the cells were incubated with Alexa 488-labeled DNA (250 nM for the DNA library, and 1–100 nM or 250 nM for each aptamer) in 100 µL binding buffer at 4°C for 30 min. After the incubation, the cells were washed three times with ice-cold washing buffer, re-suspended in 200 µL binding buffer, and then filtered with a 40-µm cell strainer (BD Falcon) to remove clumped cells. The fluorescence intensity of the DNA bound to the cells was monitored with a cell sorter, S3e (Bio-Rad), by counting 10,000 events. The obtained data were analyzed with the FCS Express software (De Novo Software). Dissociation constant (K_D) values were calculated by curve fitting, using the OriginPro 9 software (OriginLab) according to the following formula: $Y = B_{max}X/(K_D + X)$, where X is the DNA concentration, and Y is the geometric mean fluorescence intensity (MFI). The MFI without DNA was subtracted from that with each aptamer, and these values were plotted and used for curve fitting.

Deep Sequencing

The sequences enriched through cell-ExSELEX were determined by deep sequencing with the Ion PGM sequencer system. After the seventh round of selection, the DNA fragments bound to the target cells were collected by cell sorting with the S3e cell sorter, with the gating of the shifted fluorescence intensity population against the negative controls. The sorted fragments were then used as templates for replacement PCR to substitute the unnatural Ds bases with natural bases, which allows the application of conventional DNA sequencing methods. The details of the PCR conditions were previously described.[3,5] The PCR products were purified with the QIAquick Gel Purification Kit (QIAGEN) and used as a template for emulsion PCR with the Ion OneTouch System (Life Technologies), according to the manufacturer's instructions. The sequencing data were obtained by using IonPGM 314 chips.

Screening of Potential Aptamer Candidates

Based on an analysis of the obtained deep-sequencing data, several different aptamer candidates were chosen, and each sequence was chemically synthesized with an amino modification. After purification by denaturing gel electrophoresis, the DNA was conjugated with Alexa 488 NHS Ester (Molecular Probes) and purified by denaturing gel electrophoresis. Each Alexa 488-labeled aptamer was subjected to a binding analysis with the S3e cell sorter, as described earlier.

Doped Cell-ExSELEX

For further optimization and characterization of each aptamer candidate, partially randomized, doped libraries corresponding to 08B-MCF7, 14A-MCF7, 05-MB231, 07-MB231, 23-MB231, and 03-T47D were prepared, as described previously.[3] By using each doped library, the second cell-ExSELEX (doped cell-ExSELEX) was performed using procedures similar to those in the first selection and the conditions indicated in Tables S2–S4. The sequences of the DNA library after the fourth round of selection were analyzed as described earlier, and for each aptamer, the optimal sequence and its probable secondary structure were estimated from the sequence patterns at each nucleotide position.

Microscopic Fluorescent Imaging

To visualize and compare the binding specificity of each selected aptamer, fluorescence images of various cell lines bound with the aptamer were observed by fluorescence microscopy (Eclipse Ti, Nikon) or confocal laser microscopy (FV3000 or FV10i-DOC, Olympus; A1R+ Confocal Microscope, Nikon). Each cell line was cultured on an 8-well chamber slide (Corning) and incubated with 250 nM Alexa 488-labeled aptamer, either on ice or at 37°C for 30 min. After the

incubation, the supernatant was removed and the cells were washed with ice-cold washing buffer three times. The cells were fixed with 4% formaldehyde (Nacalai Tesque, Kyoto, Japan) for 10 min at room temperature, stained with DAPI (Dojindo Laboratories, Kumamoto, Japan), and mounted with VECTASHIELD mounting medium (Vector Laboratories, Burlingame, CA, USA). In the confocal imaging, prior to the treatment with the aptamer, the cells were incubated at 37°C for 30 min in the presence of 75 nM LysoTracker Red DND-99 (Molecular Probes).

Cytostatic Activity Analysis

The cytostatic activity of representative aptamers against each cell line was examined through WST-8 assays, as follows. Aliquots (0.1 mL) of each cell solution (1–2.5 $\times$ 10^4 cells per milliliter for cancer cell lines, except for KG-1, and 4 $\times$ 10^4 cells per milliliter for HUVEC and MCF10A cells) were plated into each well of a 96-well microplate, 1 day before the addition of DNA aptamers. In the case of KG-1 cells, 90 μL cell solution was plated into each well, 4 hr before the DNA addition. Each DNA aptamer was folded in D-PBS (−), similar to the DNA library used for cell-ExSELEX, at a 0.1mM concentration. The folded DNA aptamer, as well as FUdR (Nacalai Tesque), was used as a positive drug control and was diluted with D-PBS (−) and the medium used for each cell line, and added to the cells at final concentrations of 1, 2.5, 5, or 10 μM for DNA and 10 or 100 nM for FUdR in 10% D-PBS (−)/media (0.1 mL per well). As another positive control, PTX (Wako Pure Chemical Industries) in DMSO was also used for the analysis and added at the final concentration of 10 nM in 0.1% DMSO/media (0.1 mL per well). After incubation for 96 hr, 10 μL Cell Count Reagent SF (Nacalai Tesque) was added to each well, and the absorbance of the formazan product obtained by WST-8 reduction was measured at 450 nm with an ARVO multilabel counter (PerkinElmer).

SUPPLEMENTAL INFORMATION

Supplemental Information includes twenty seven figures and five tables and can be found with this article online at https://doi.org/10.1016/j.omtn.2018.11.011.

AUTHOR CONTRIBUTIONS

All of the authors conducted the experiments and analyzed the data sets. M.K. and I.H. conceived the project, designed methods and experiments, and wrote the manuscript. I.H. supervised the entire project.

CONFLICT OF INTERESTS

M.K. and I.H. own stock in TAGCyx Biotechnologies. (I.H. is a founder.)

ACKNOWLEDGMENTS

We would like to thank the Olympus Corporation (Japan), the IMB microscopy unit (Agency for Science, Technology and Research, Singapore), and the SBIC-Nikon Imaging Centre (Agency for Science, Technology and Research, Singapore) for their technical support in the confocal image scanning and analysis. This work was supported by the Institute of Bioengineering and Nanotechnology (Biomedical Research Council, Agency for Science, Technology and Research, Singapore) (to M.K. and I.H.); by a grant-in-aid for scientific research (KAKENHI 26248043) from the Ministry of Education, Culture, Sports, Science and Technology (to I.H.); by grants for projects focused on developing key technologies for discovering and manufacturing drugs for next-generation treatment and diagnosis from the Ministry of Economy, Trade and Industry (to I.H.); and by the Japan Science and Technology Agency Precursory Research for Embryonic Science and Technology (JST PRESTO) (JPMJPR13K9 to M.K.).

REFERENCES

1. Zhang, Y., Ptacin, J.L., Fischer, E.C., Aerni, H.R., Caffaro, C.E., San Jose, K., Feldman, A.W., Turner, C.R., and Romesberg, F.E. (2017). A semi-synthetic organism that stores and retrieves increased genetic information. Nature *551*, 644–647.
2. Malyshev, D.A., Dhami, K., Lavergne, T., Chen, T., Dai, N., Foster, J.M., Corrêa, I.R., Jr., and Romesberg, F.E. (2014). A semi-synthetic organism with an expanded genetic alphabet. Nature *509*, 385–388.
3. Kimoto, M., Yamashige, R., Matsunaga, K., Yokoyama, S., and Hirao, I. (2013). Generation of high-affinity DNA aptamers using an expanded genetic alphabet. Nat. Biotechnol. *31*, 453–457.
4. Sefah, K., Yang, Z., Bradley, K.M., Hoshika, S., Jiménez, E., Zhang, L., Zhu, G., Shanker, S., Yu, F., Turek, D., et al. (2014). *In vitro* selection with artificial expanded genetic information systems. Proc. Natl. Acad. Sci. USA *111*, 1449–1454.
5. Matsunaga, K.I., Kimoto, M., and Hirao, I. (2017). High-affinity DNA aptamer generation targeting von Willebrand factor A1-domain by genetic alphabet expansion for systematic evolution of ligands by exponential enrichment using two types of libraries composed of five different bases. J. Am. Chem. Soc. *139*, 324–334.
6. Ellington, A.D., and Szostak, J.W. (1990). *In vitro* selection of RNA molecules that bind specific ligands. Nature *346*, 818–822.
7. Tuerk, C., and Gold, L. (1990). Systematic evolution of ligands by exponential enrichment: RNA ligands to bacteriophage T4 DNA polymerase. Science *249*, 505–510.
8. Ulrich, H., and Wrenger, C. (2009). Disease-specific biomarker discovery by aptamers. Cytometry A *75*, 727–733.
9. Sun, H., Tan, W., and Zu, Y. (2016). Aptamers: versatile molecular recognition probes for cancer detection. Analyst (Lond.) *141*, 403–415.
10. Zhu, H., Li, J., Zhang, X.B., Ye, M., and Tan, W. (2015). Nucleic acid aptamer-mediated drug delivery for targeted cancer therapy. ChemMedChem *10*, 39–45.
11. Tan, W., Donovan, M.J., and Jiang, J. (2013). Aptamers from cell-based selection for bioanalytical applications. Chem. Rev. *113*, 2842–2862.
12. Khedri, M., Rafatpanah, H., Abnous, K., Ramezani, P., and Ramezani, M. (2015). Cancer immunotherapy via nucleic acid aptamers. Int. Immunopharmacol. *29*, 926–936.
13. Gedi, V., and Kim, Y.P. (2014). Detection and characterization of cancer cells and pathogenic bacteria using aptamer-based nano-conjugates. Sensors (Basel) *14*, 18302–18327.
14. Vater, A., and Klussmann, S. (2015). Turning mirror-image oligonucleotides into drugs: the evolution of Spiegelmer(®) therapeutics. Drug Discov. Today *20*, 147–155.
15. Tolle, F., Brändle, G.M., Matzner, D., and Mayer, G. (2015). A versatile approach towards nucleobase-modified aptamers. Angew. Chem. Int. Ed. Engl. *54*, 10971–10974.
16. Rohloff, J.C., Gelinas, A.D., Jarvis, T.C., Ochsner, U.A., Schneider, D.J., Gold, L., and Janjic, N. (2014). Nucleic acid ligands with protein-like side chains: modified aptamers and their use as diagnostic and therapeutic agents. Mol. Ther. Nucleic Acids *3*, e201.
17. Taylor, A.I., Arangundy-Franklin, S., and Holliger, P. (2014). Towards applications of synthetic genetic polymers in diagnosis and therapy. Curr. Opin. Chem. Biol. *22*, 79–84.

18. Meek, K.N., Rangel, A.E., and Heemstra, J.M. (2016). Enhancing aptamer function and stability via in vitro selection using modified nucleic acids. Methods *106*, 29–36.

19. Lee, K.H., Hamashima, K., Kimoto, M., and Hirao, I. (2018). Genetic alphabet expansion biotechnology by creating unnatural base pairs. Curr. Opin. Biotechnol. *51*, 8–15.

20. Malyshev, D.A., and Romesberg, F.E. (2015). The expanded genetic alphabet. Angew. Chem. Int. Ed. Engl. *54*, 11930–11944.

21. Laos, R., Thomson, J.M., and Benner, S.A. (2014). DNA polymerases engineered by directed evolution to incorporate non-standard nucleotides. Front. Microbiol. *5*, 565.

22. Hirao, I., and Kimoto, M. (2012). Unnatural base pair systems toward the expansion of the genetic alphabet in the central dogma. Proc. Jpn. Acad., Ser. B Phys. Biol. Sci. *88*, 345–367.

23. Georgiadis, M.M., Singh, I., Kellett, W.F., Hoshika, S., Benner, S.A., and Richards, N.G. (2015). Structural basis for a six nucleotide genetic alphabet. J. Am. Chem. Soc. *137*, 6947–6955.

24. Kimoto, M., Kawai, R., Mitsui, T., Yokoyama, S., and Hirao, I. (2009). An unnatural base pair system for efficient PCR amplification and functionalization of DNA molecules. Nucleic Acids Res. *37*, e14.

25. Yamashige, R., Kimoto, M., Takezawa, Y., Sato, A., Mitsui, T., Yokoyama, S., and Hirao, I. (2012). Highly specific unnatural base pair systems as a third base pair for PCR amplification. Nucleic Acids Res. *40*, 2793–2806.

26. Zhang, L., Yang, Z., Sefah, K., Bradley, K.M., Hoshika, S., Kim, M.J., Kim, H.J., Zhu, G., Jiménez, E., Cansiz, S., et al. (2015). Evolution of functional six-nucleotide DNA. J. Am. Chem. Soc. *137*, 6734–6737.

27. Zhang, L., Yang, Z., Le Trinh, T., Teng, I.T., Wang, S., Bradley, K.M., Hoshika, S., Wu, Q., Cansiz, S., Rowold, D.J., et al. (2016). Aptamers against cells overexpressing glypican 3 from expanded genetic systems combined with cell engineering and laboratory evolution. Angew. Chem. Int. Ed. Engl. *55*, 12372–12375.

28. Kimoto, M., Matsunaga, K., and Hirao, I. (2016). DNA aptamer generation by genetic alphabet expansion SELEX (ExSELEX) using an unnatural base pair system. Methods Mol. Biol. *1380*, 47–60.

29. Hirao, I., Madin, K., Endo, Y., Yokoyama, S., and Ellington, A.D. (2000). RNA aptamers that bind to and inhibit the ribosome-inactivating protein, pepocin. J. Biol. Chem. *275*, 4943–4948.

30. Hirao, I., Kawai, G., Yoshizawa, S., Nishimura, Y., Ishido, Y., Watanabe, K., and Miura, K. (1994). Most compact hairpin-turn structure exerted by a short DNA fragment, d(GCGAAGC) in solution: an extraordinarily stable structure resistant to nucleases and heat. Nucleic Acids Res. *22*, 576–582.

31. Yoshizawa, S., Ueda, T., Ishido, Y., Miura, K., Watanabe, K., and Hirao, I. (1994). Nuclease resistance of an extraordinarily thermostable mini-hairpin DNA fragment, d(GCGAAGC) and its application to *in vitro* protein synthesis. Nucleic Acids Res. *22*, 2217–2221.

32. Yoshizawa, S., Kawai, G., Watanabe, K., Miura, K., and Hirao, I. (1997). GNA trinucleotide loop sequences producing extraordinarily stable DNA minihairpins. Biochemistry *36*, 4761–4767.

33. Matsunaga, K., Kimoto, M., Hanson, C., Sanford, M., Young, H.A., and Hirao, I. (2015). Architecture of high-affinity unnatural-base DNA aptamers toward pharmaceutical applications. Sci. Rep. *5*, 18478.

34. Kimoto, M., Nakamura, M., and Hirao, I. (2016). Post-ExSELEX stabilization of an unnatural-base DNA aptamer targeting VEGF165 toward pharmaceutical applications. Nucleic Acids Res. *44*, 7487–7494.

35. Reyes-Reyes, E.M., Teng, Y., and Bates, P.J. (2010). A new paradigm for aptamer therapeutic AS1411 action: uptake by macropinocytosis and its stimulation by a nucleolin-dependent mechanism. Cancer Res. *70*, 8617–8629.

36. Kimoto, M., Moriyama, K., Yokoyama, S., and Hirao, I. (2007). Cytostatic evaluations of nucleoside analogs related to unnatural base pairs for a genetic expansion system. Bioorg. Med. Chem. Lett. *17*, 5582–5585.

37. Nozari, A., and Berezovski, M.V. (2017). Aptamers for CD antigens: from cell profiling to activity modulation. Mol. Ther. Nucleic Acids *6*, 29–44.

38. Welton, J.L., Brennan, P., Gurney, M., Webber, J.P., Spary, L.K., Carton, D.G., Falcón-Pérez, J.M., Walton, S.P., Mason, M.D., Tabi, Z., and Clayton, A. (2016). Proteomics analysis of vesicles isolated from plasma and urine of prostate cancer patients using a multiplex, aptamer-based protein array. J. Extracell. Vesicles *5*, 31209.

39. Billing, A.M., Ben Hamidane, H., Bhagwat, A.M., Cotton, R.J., Dib, S.S., Kumar, P., Hayat, S., Goswami, N., Suhre, K., Rafii, A., and Graumann, J. (2017). Complementarity of SOMAscan to LC-MS/MS and RNA-seq for quantitative profiling of human embryonic and mesenchymal stem cells. J. Proteomics *150*, 86–97.

40. Jiang, Y., Shi, M., Liu, Y., Wan, S., Cui, C., Zhang, L., and Tan, W. (2017). Aptamer/AuNP biosensor for colorimetric profiling of exosomal proteins. Angew. Chem. Int. Ed. Engl. *56*, 11916–11920.

41. Li, X., Zhang, W., Liu, L., Zhu, Z., Ouyang, G., An, Y., Zhao, C., and Yang, C.J. (2014). *In vitro* selection of DNA aptamers for metastatic breast cancer cell recognition and tissue imaging. Anal. Chem. *86*, 6596–6603.

42. Civit, L., Taghdisi, S.M., Jonczyk, A., Hassel, S.K., Grober, C., Blank, M., Beyer, M., Schultze, J., Latz, E., and Mayer, G. (2018). Systematic evaluation of cell-SELEX enriched aptamers binding to breast cancer cells. Biochimie *145*, 53–62.

43. Yuan, B., Jiang, X., Chen, Y., Guo, Q., Wang, K., Meng, X., Huang, Z., and Wen, X. (2017). Metastatic cancer cell and tissue-specific fluorescence imaging using a new DNA aptamer developed by Cell-SELEX. Talanta *170*, 56–62.

44. Hirao, I., Kimoto, M., Mitsui, T., Fujiwara, T., Kawai, R., Sato, A., Harada, Y., and Yokoyama, S. (2006). An unnatural hydrophobic base pair system: site-specific incorporation of nucleotide analogs into DNA and RNA. Nat. Methods *3*, 729–735.

OMTN, Volume *14*

Supplemental Information

Genetic Alphabet Expansion Provides Versatile Specificities and Activities of Unnatural-Base DNA Aptamers Targeting Cancer Cells

Kazunobu Futami, Michiko Kimoto, Yun Wei Shermane Lim, and Ichiro Hirao

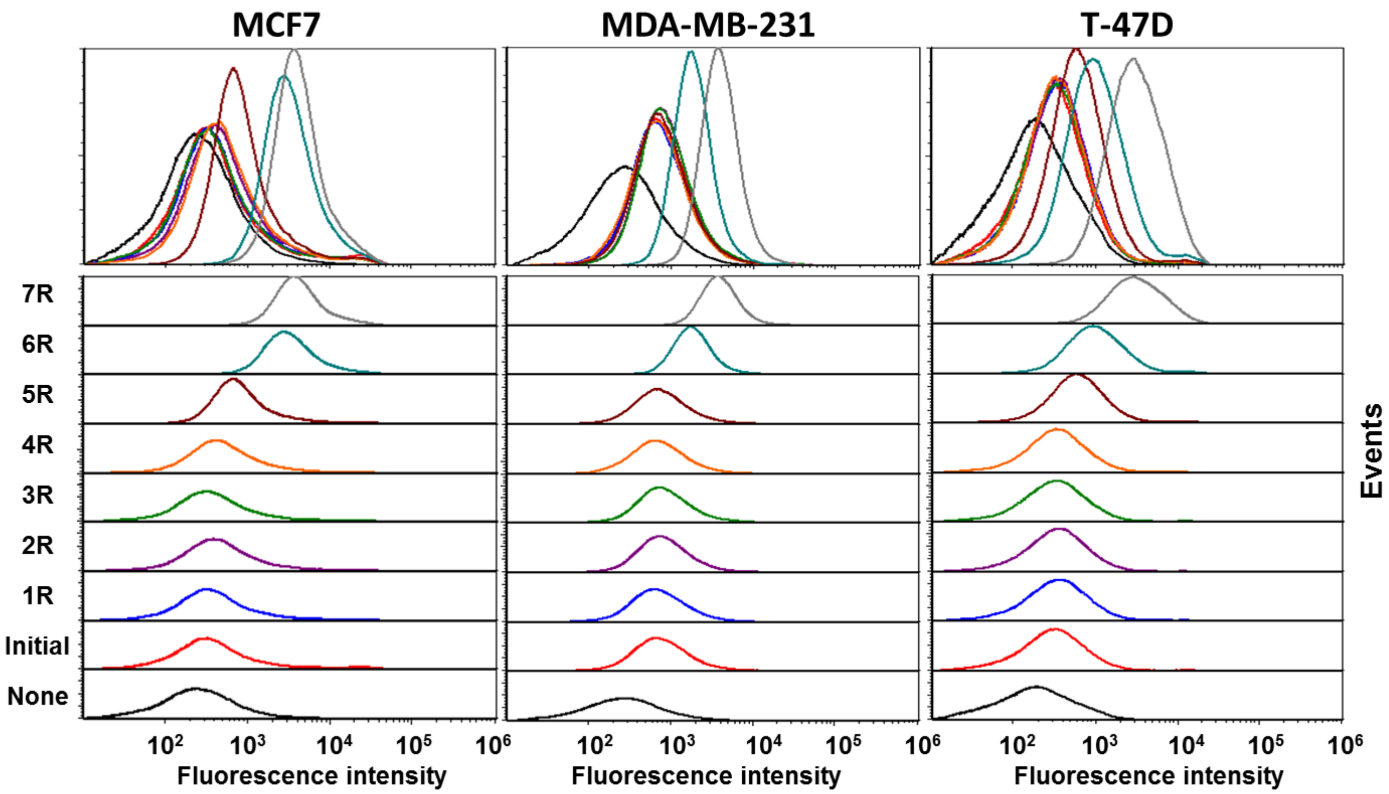

Figure S1: Monitoring the binding ability of the libraries after each cell-ExSELEX round with flow cytometry. Alexa488-labeled libraries (250 nM) were incubated with target cells at 4ºC for 30 min, washed twice, and subjected to flow cytometry analyses.

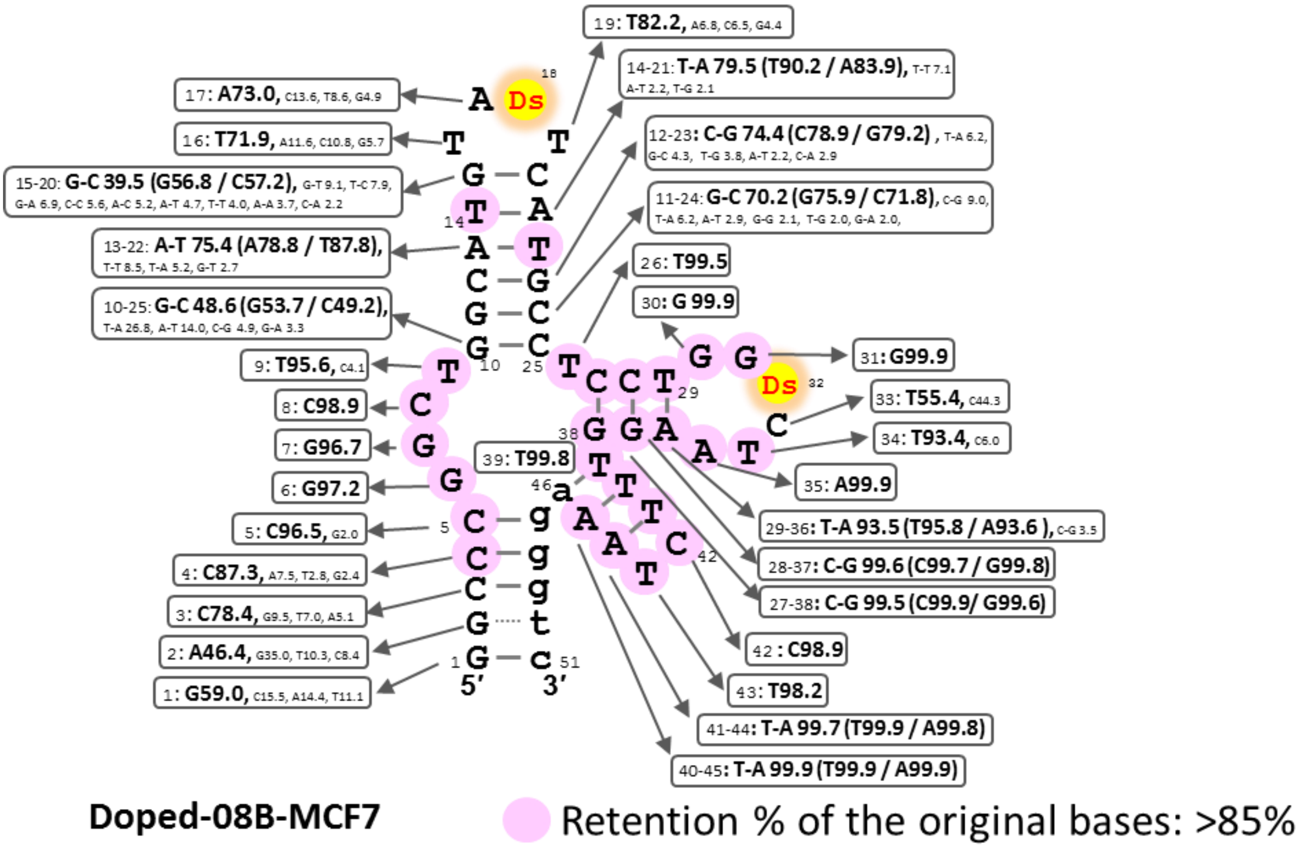

Figure S2: Predicted secondary structure of 08B-MCF7, based on highly retained nucleobases and co-varied nucleobases in sequences obtained in the doped second selection targeting MCF7. Pink circles indicate the bases with more than 85% retention after the doped selection. Small letters belong to the 3′-primer region. The ratios (more than 2%) of base pairs or bases in the putative stem or loop regions are summarized in the enclosed boxes. The base pairs or bases with the highest ratio are shown in bold.

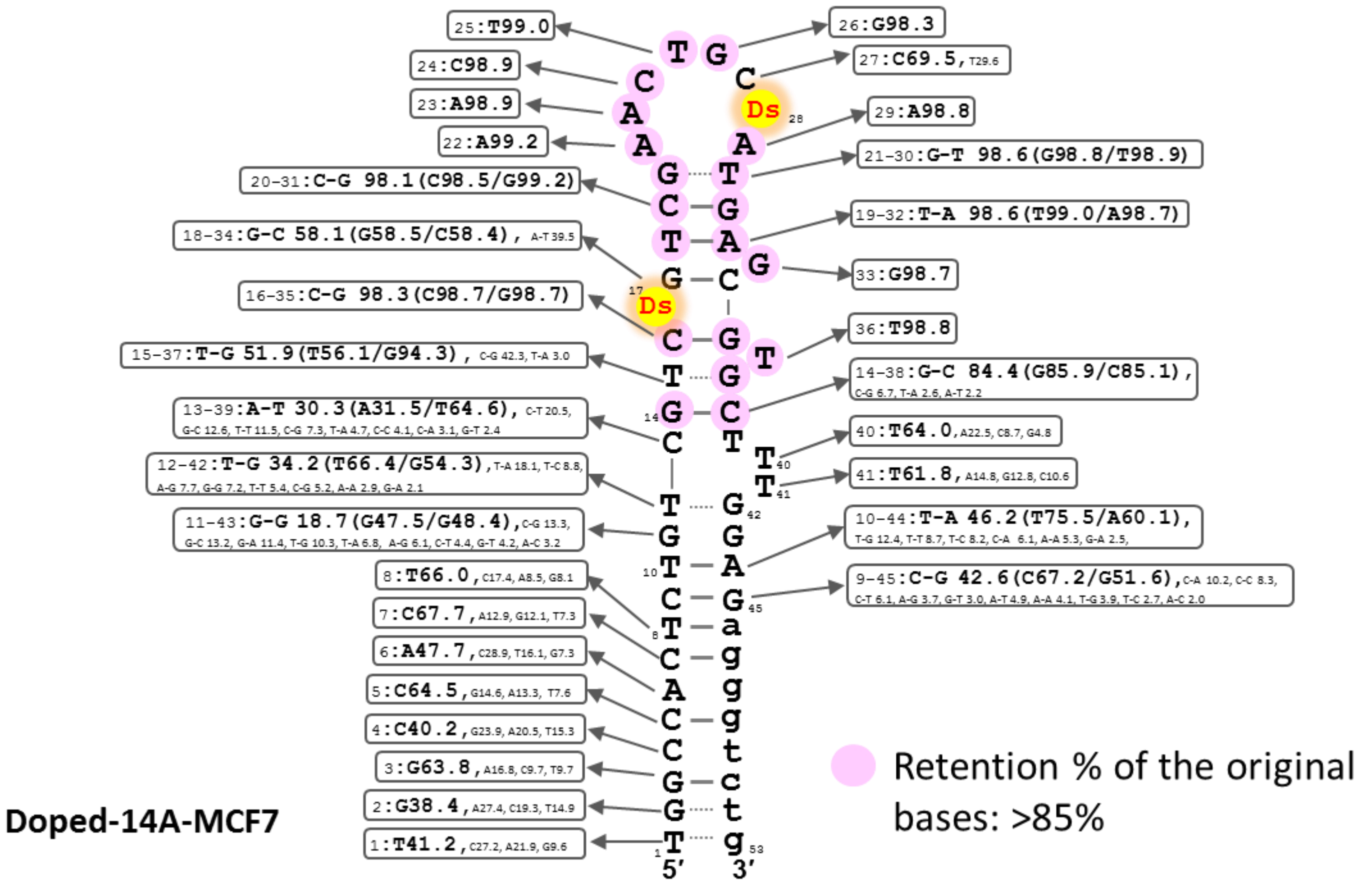

Figure S3: Predicted secondary structure of 14A-MCF7, based on highly retained nucleobases and co-varied nucleobases in sequences obtained in the doped second selection targeting MCF7. Pink circles indicate the bases with more than 85% retention after the doped selection. Small letters belong to the 3′-primer region. The ratios (more than 2%) of base pairs or bases in the putative stem or loop regions are summarized in the enclosed boxes. The base pairs or bases with the highest ratio are shown in bold.

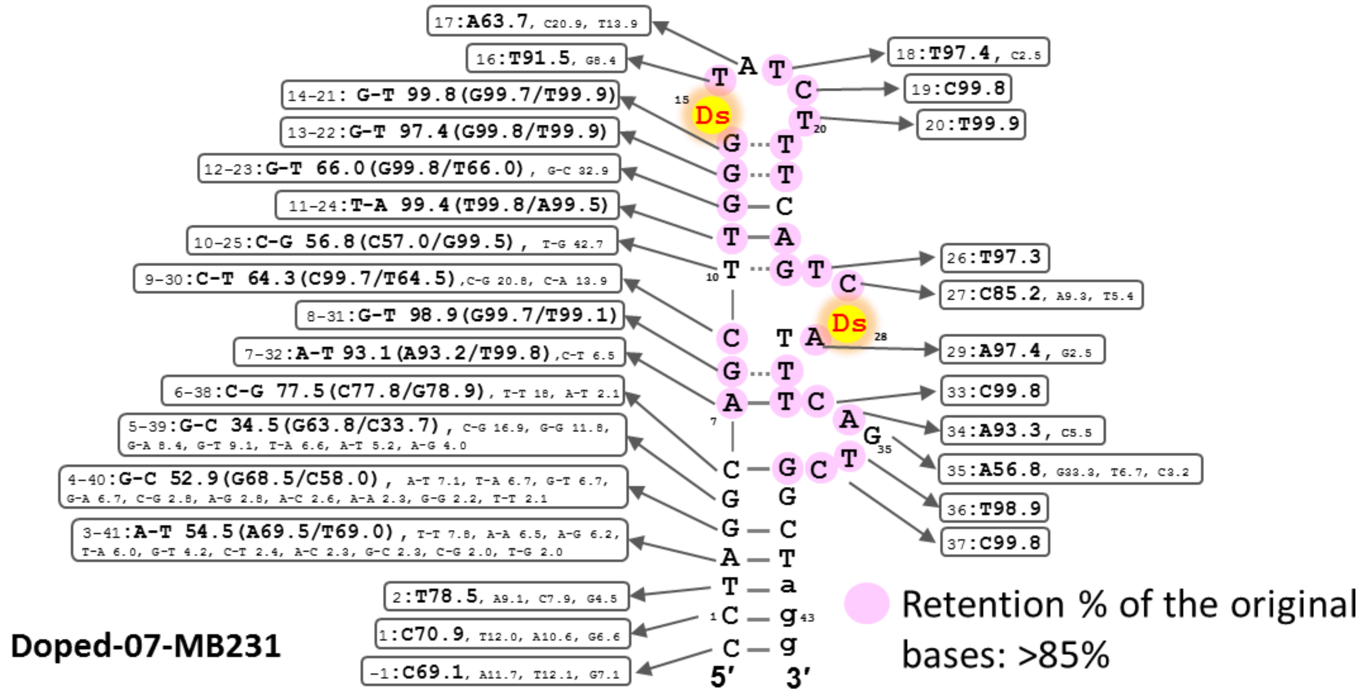

Figure S4: Predicted secondary structure of 07-MB231, based on highly retained nucleobases and co-varied nucleobases in sequences obtained in the doped second selection targeting MDA-MB-231. Pink circles indicate the bases with more than 85% retention after the doped selection. Small letters belong to the 3′-primer region. The ratios (more than 2%) of base pairs or bases in the putative stem or loop regions are summarized in the enclosed boxes. The base pairs or bases with the highest ratio are shown in bold.

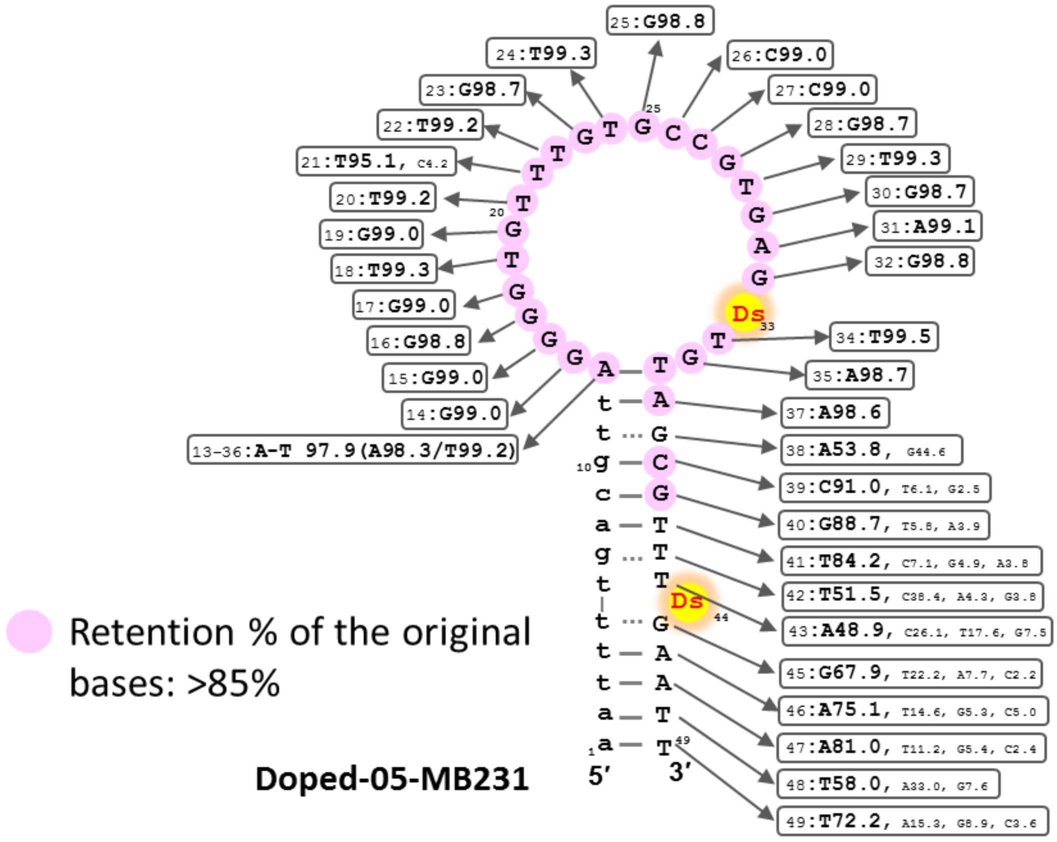

Figure S5: Predicted secondary structure of 05-MB231, based on highly retained nucleobases and co-varied nucleobases in sequences obtained in the doped second selection targeting MDA-MB-231. Pink circles indicate the bases with more than 85% retention after the doped selection. Small letters belong to the 5′-primer region. The ratios (more than 2%) of base pairs or bases in the putative stem or loop regions are summarized in the enclosed boxes. The base pairs or bases with the highest ratio are shown in bold.

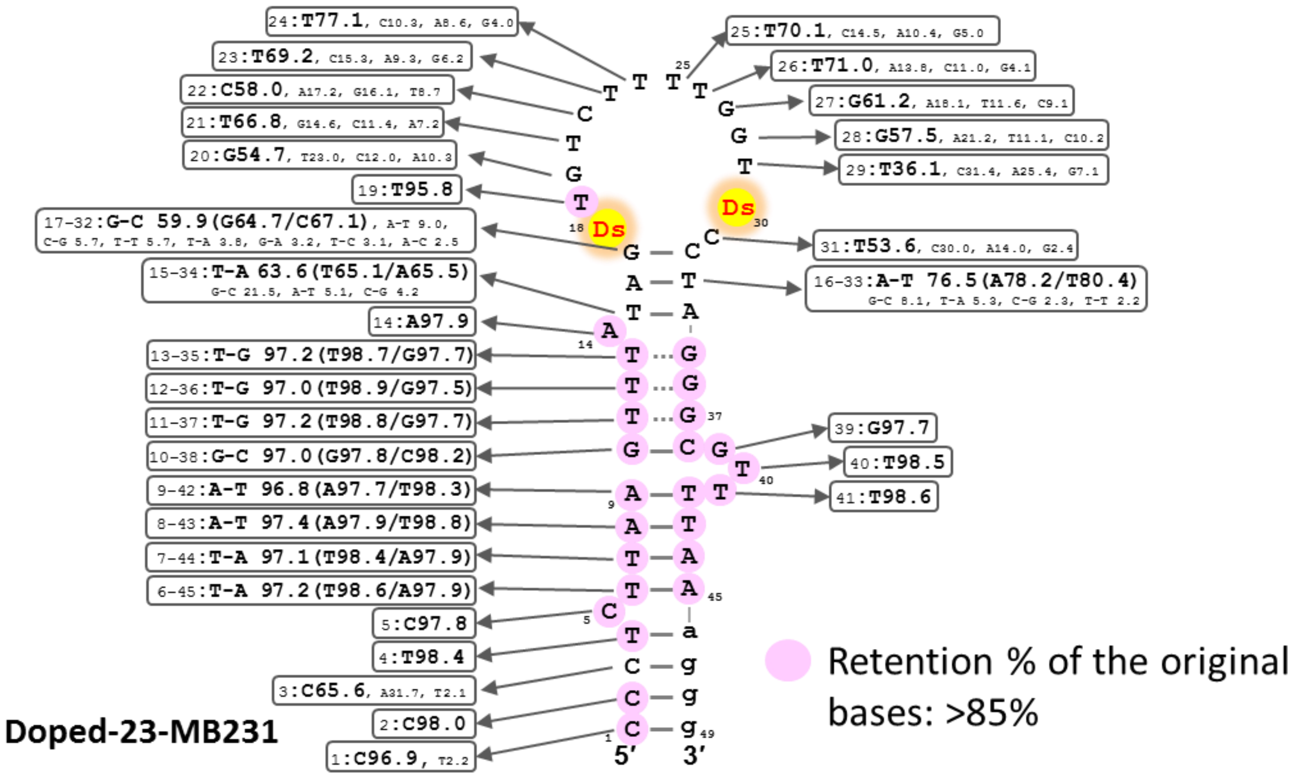

Figure S6: Predicted secondary structure of 23-MB231, based on highly retained nucleobases and co-varied nucleobases in sequences obtained in the doped second selection targeting MDA-MB-231. Pink circles indicate the bases with more than 85% retention after the selection. Small letters belong to the 3′-primer region. The ratios (more than 2%) of base pairs or bases in the putative stem or loop regions are summarized in the enclosed boxes. The base pairs or bases with the highest ratio are shown in bold.

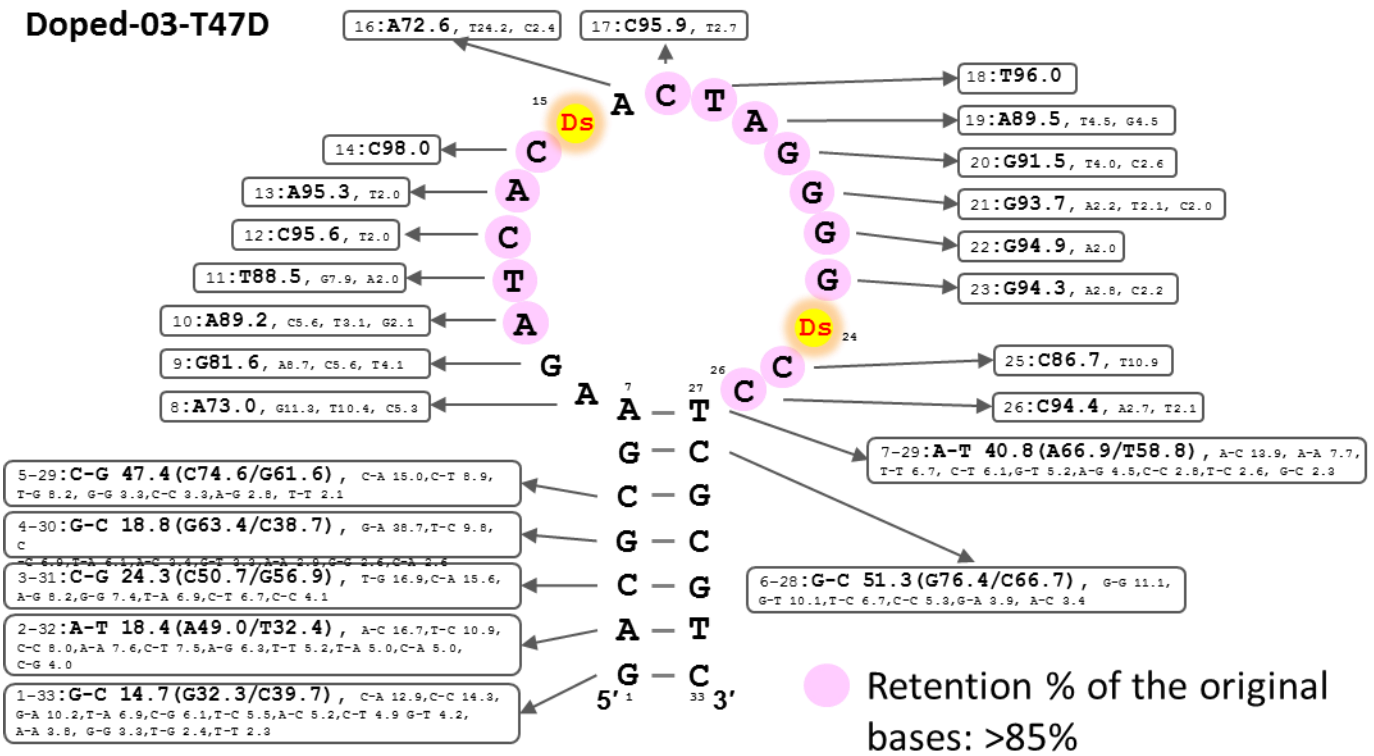

Figure S7: Predicted secondary structure of 03-T47D, based on highly retained nucleobases and co-varied nucleobases in sequences obtained in the doped second selection targeting T-47D. Pink circles indicate the bases with more than 85% retention after the doped selection. The ratios (more than 2%) of base pairs or bases in the putative stem or loop regions are summarized in the enclosed boxes. The base pairs or bases with the highest ratio are shown in bold.

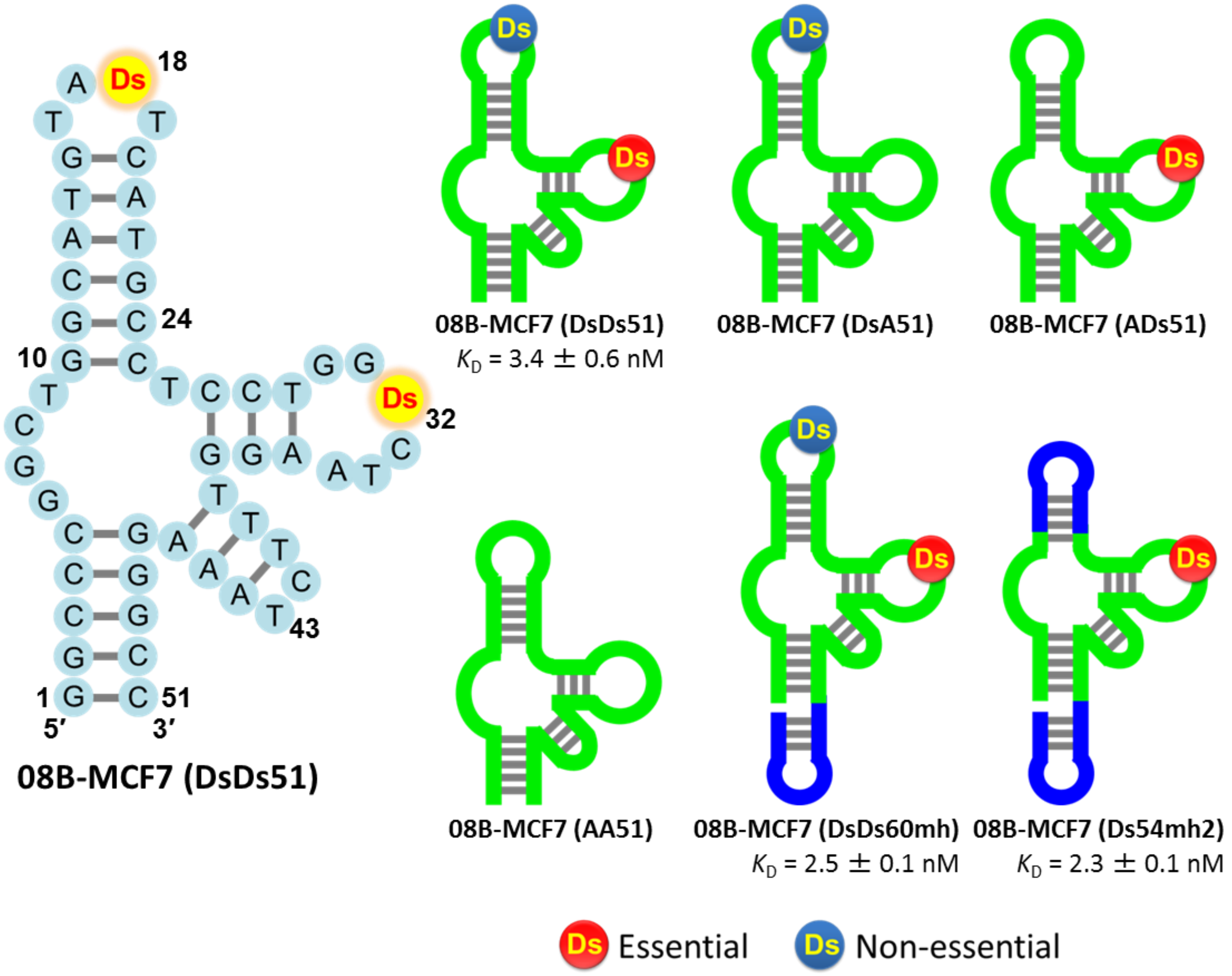

Figure S8: Schematic illustration of the secondary structures of 08B-MCF7 and its variants. The sequence and estimated secondary structures of 08B-MCF7(DsDs51) are shown on the left, and each variant is schematically represented on the right, along with its dissociation constant (K_D) to the target cell MCF7. The regions modified with a mini-hairpin are indicated by blue lines. In the DsA51, ADs51, and AA51 variants, the corresponding Ds base is replaced with A. The importance of the Ds bases for the binding was judged using the flow cytometry analysis results (see Figure S14).

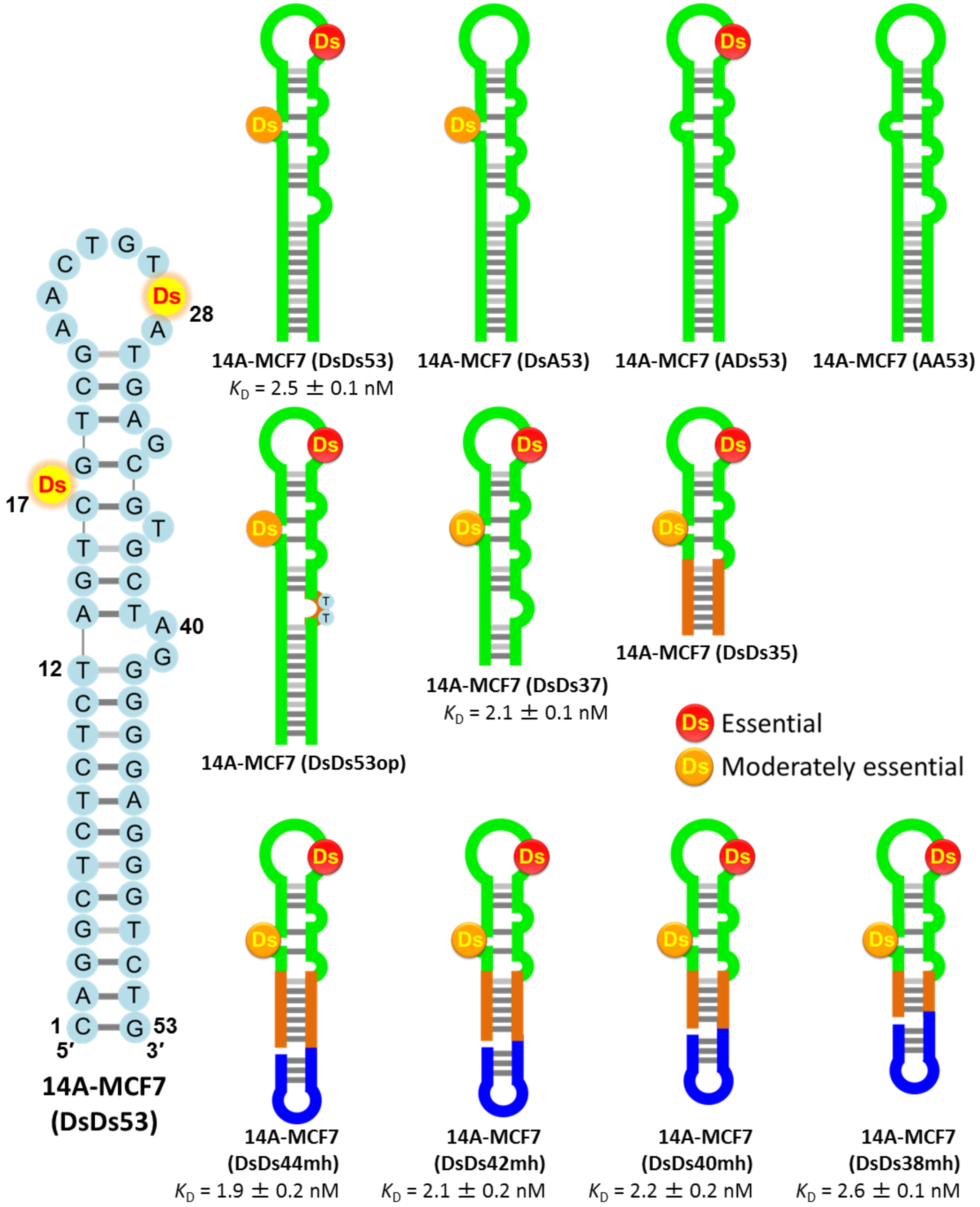

Figure S9: Schematic illustration of the secondary structures of 14A-MCF7 and its variants. The sequence and estimated secondary structures of 14A-MCF7(DsDs53) are shown on the left, and each variant is schematically represented on the right, along with its dissociation constant (K_D) to the target cell MCF7. The regions modified with a mini-hairpin DNA and G–C stem (A–T replaced with G–C) are indicated by blue and brown lines, respectively. The G–T pairing is indicated with light grey lines. In the DsA53, ADs53, and AA53 variants, the corresponding Ds base is replaced with A. The importance of the Ds bases for the binding was judged using the flow cytometry analysis results (see Figure S14).

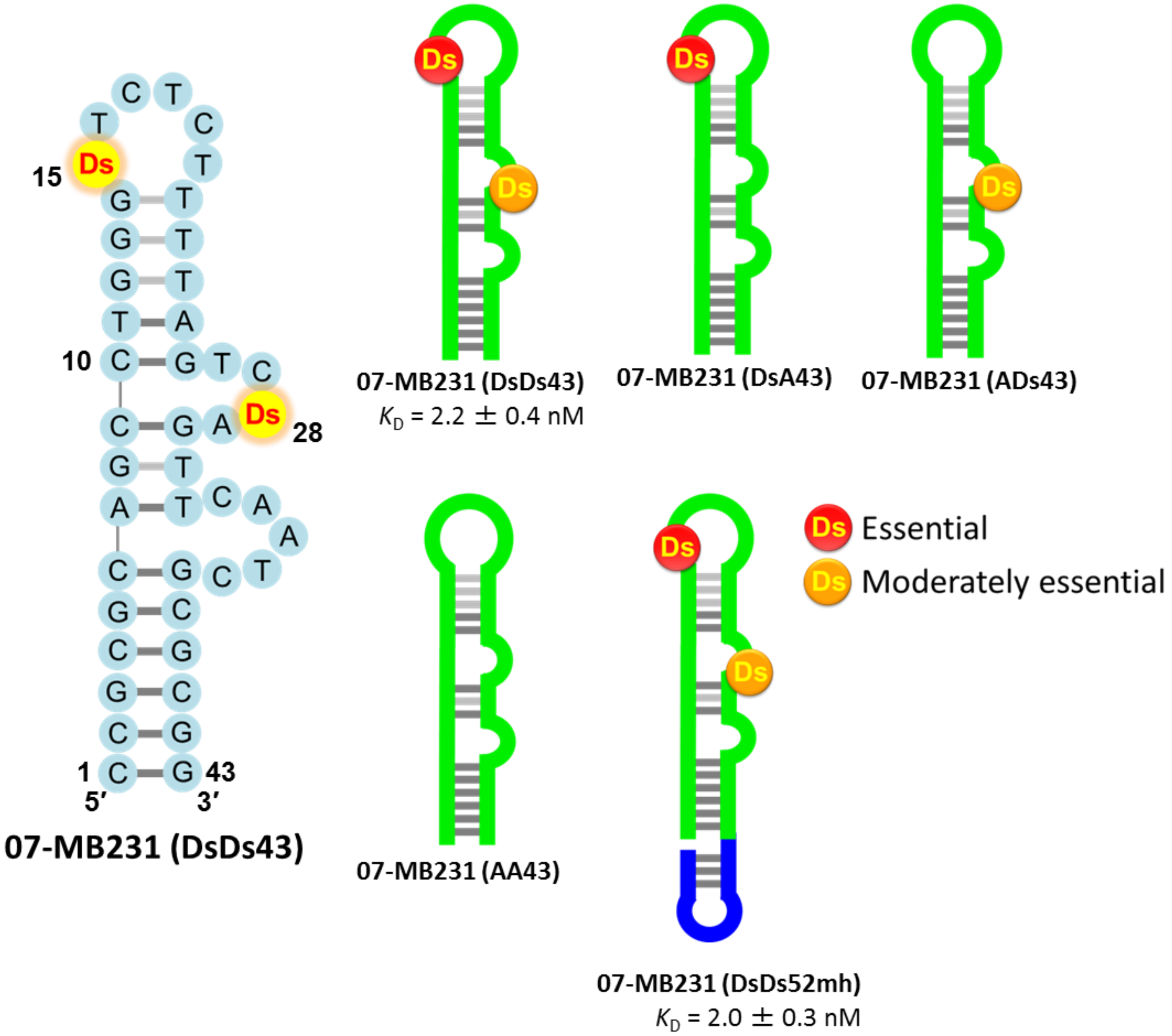

Figure S10: Schematic illustration of the secondary structures of 07-MB231 and its variants. The sequence and estimated secondary structures of 07-MB231(DsDs43) are shown on the left, and each variant is schematically represented on the right, along with its dissociation constant (K_D) to the target cell MDA-MB-231. The region modified with a mini-hairpin DNA is indicated in blue. The G–T pairing is indicated with light grey lines. In the DsA43, ADs43, and AA43 variants, the corresponding Ds base is replaced with A. The importance of the Ds bases for the binding was judged using the flow cytometry analysis results (see Figure S14).

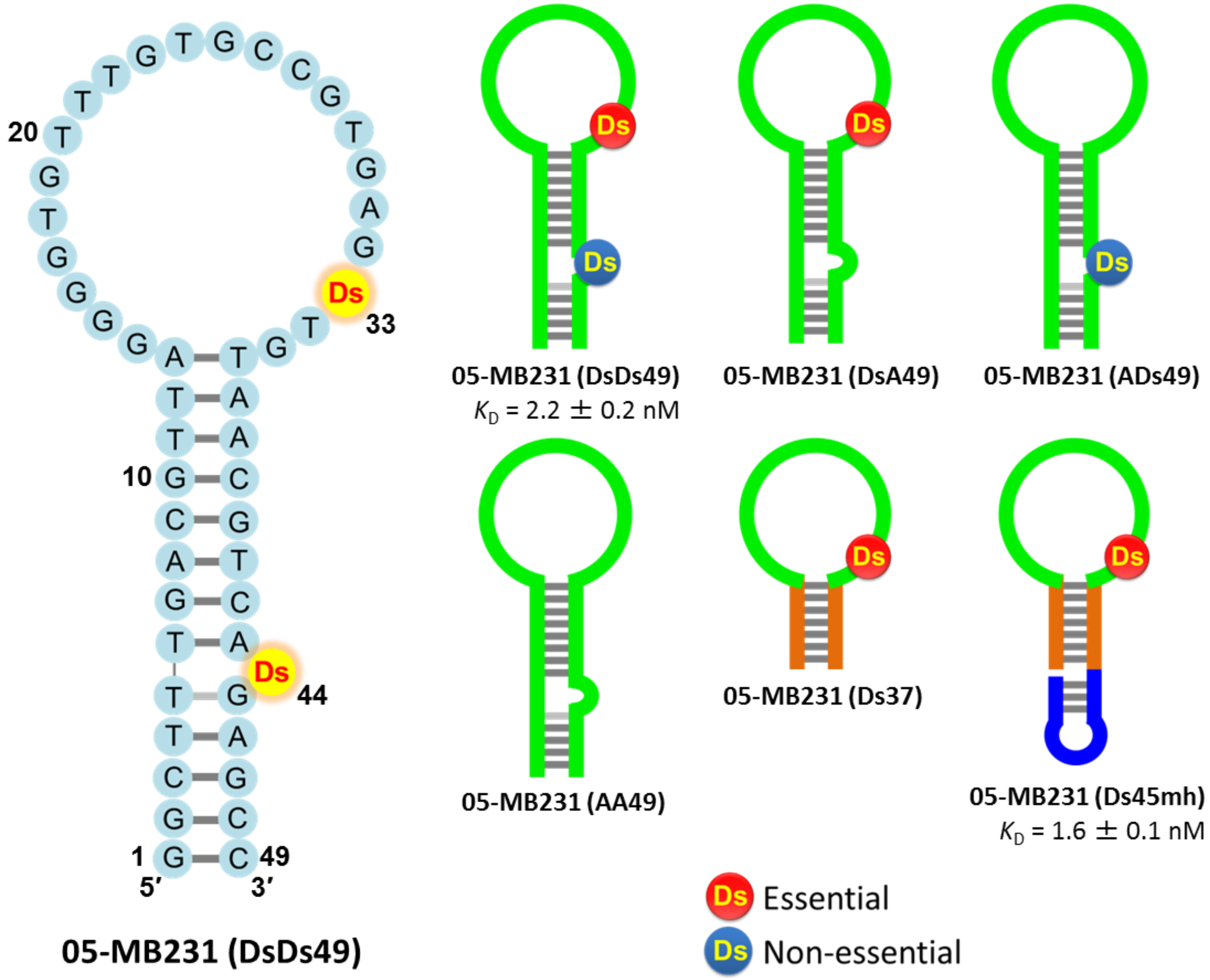

Figure S11: Schematic illustration of the secondary structures of 05-MB231 and its variants. The sequence and estimated secondary structures of 05-MB231(DsDs49) are shown on the left, and each variant is schematically represented on the right, along with its dissociation constant (K_D) to the target cell MDA-MB-231. The regions modified with a mini-hairpin DNA and a G–C stem (A–T replaced with G–C) are indicated by blue and brown lines, respectively. The G–T pairing is indicated with light grey lines. In the DsA49, ADs49, and AA49 variants, the corresponding Ds base is replaced with A. The importance of the Ds bases for the binding was judged using the flow cytometry analysis results (see Figure S14).

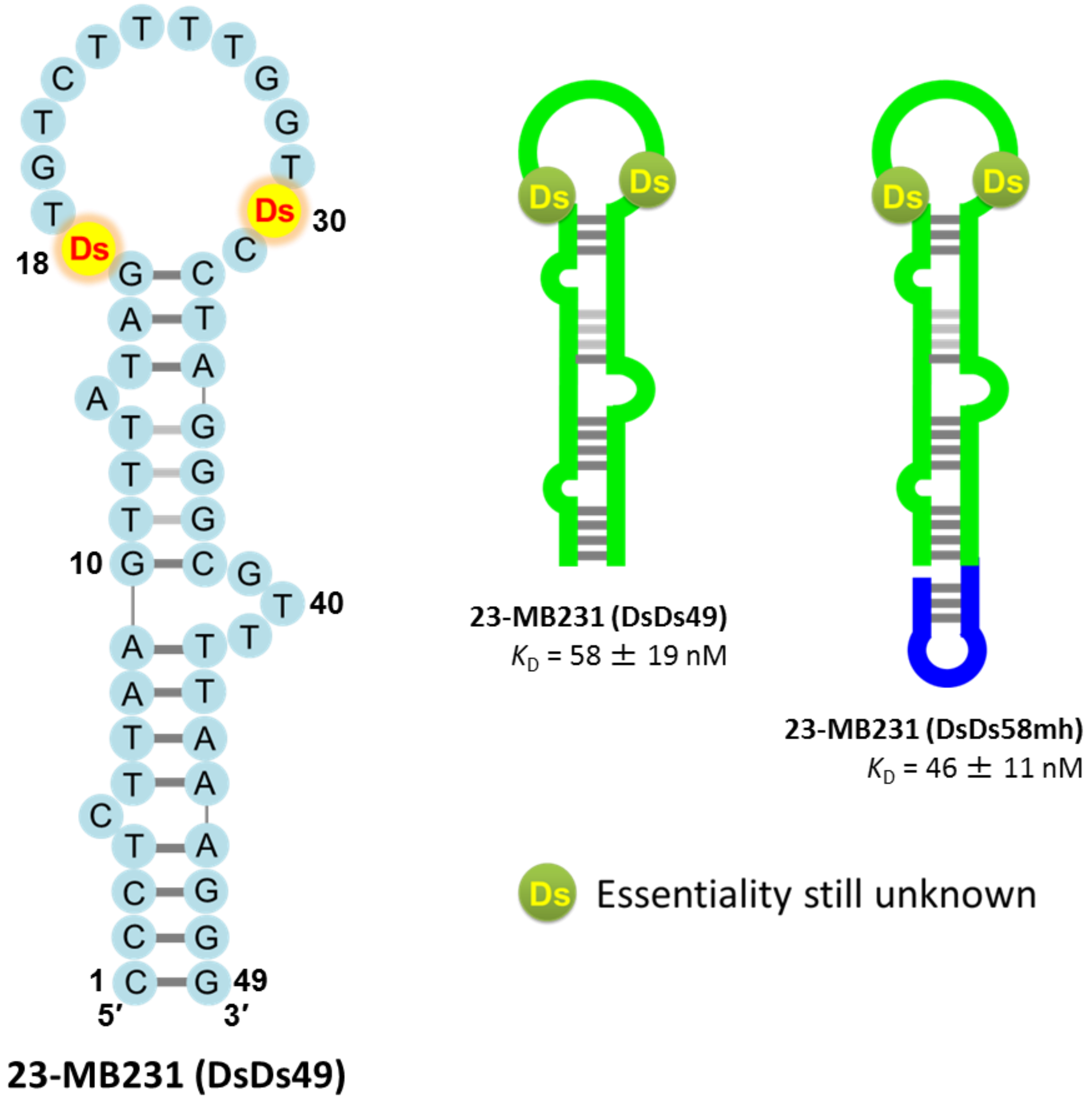

Figure S12: Schematic illustration of the secondary structures of 23-MB231 and its variants. The sequence and estimated secondary structures of 23-MB231(DsDs49) are shown on the left, and each variant is schematically represented on the right, along with its dissociation constant (K_D) to the target cell MDA-MB-231. The region modified with the mini-hairpin DNA is indicated in blue. The G–T pairing is indicated with light grey lines.

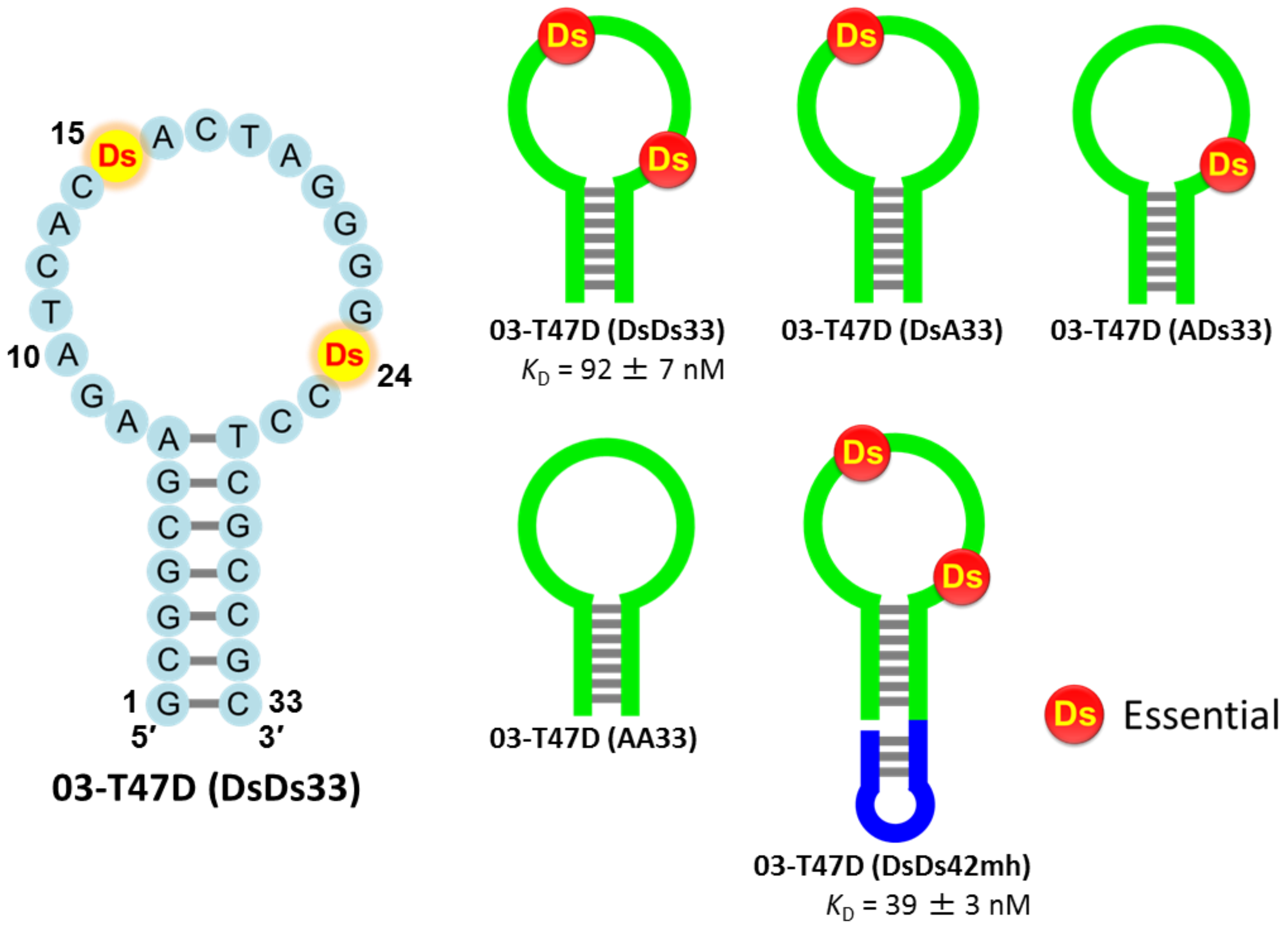

Figure S13: Schematic illustration of the secondary structures of 03-T47D and its variants. The sequence and estimated secondary structures of 03-T47D(DsDs33) are shown on the left, and each variant is schematically represented on the right, along with its dissociation constant (K_D) to the target cell T-47D. The region modified with the mini-hairpin DNA is indicated in blue. In the DsA33, ADs33, and AA33 variants, the corresponding Ds base is replaced with A. The importance of the Ds bases for the binding was judged using the flow cytometry analysis results (see Figure S14).

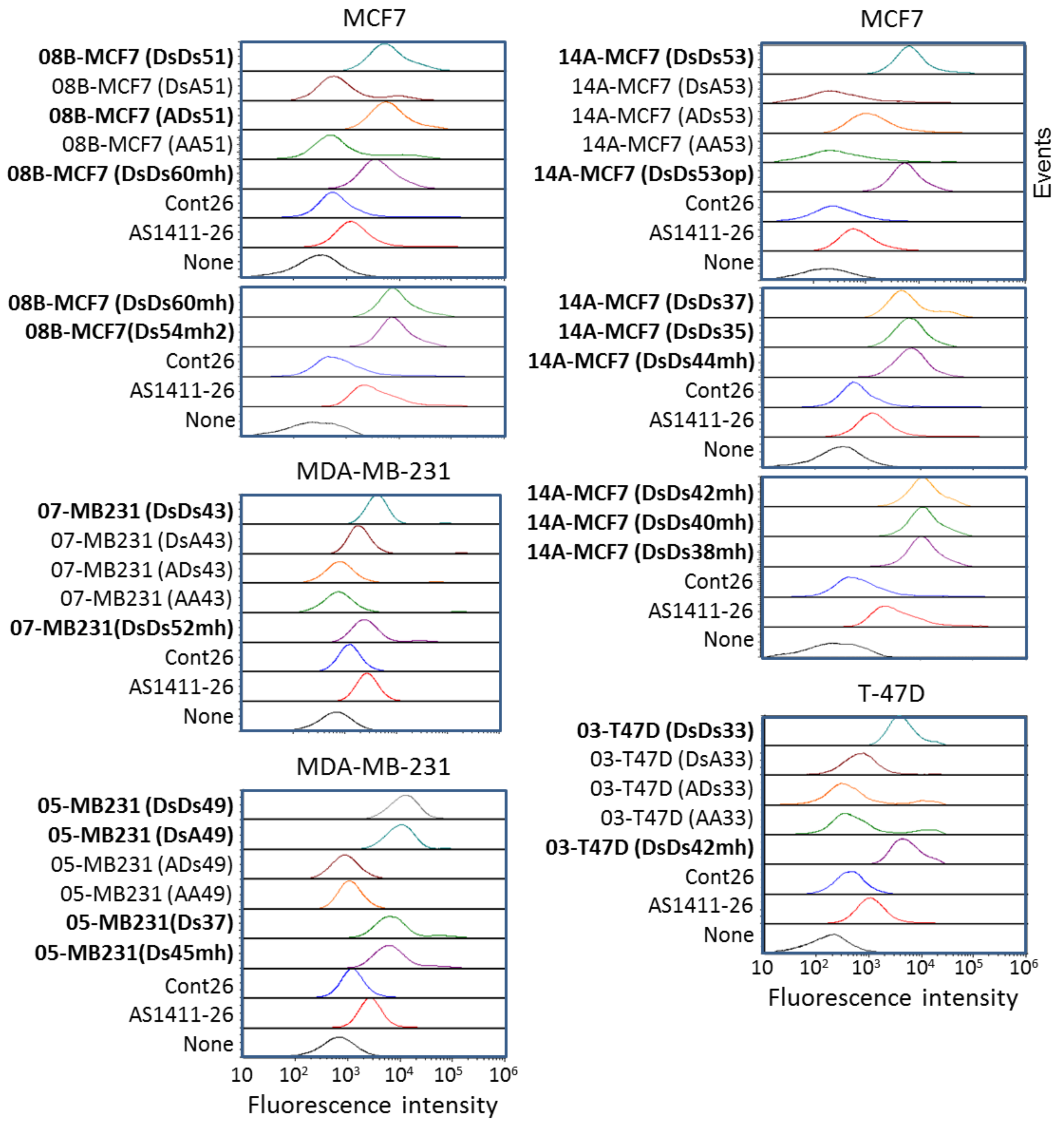

Figure S14: Binding of Ds-DNA aptamers and their variants to target cell lines. The target cells were incubated with the Alexa-488 labeled Ds-DNA aptamer and its variants (250 nM, the sequences are shown in Figures S8–S13) for 30 min at 4ºC, and then analyzed by flow cytometry.

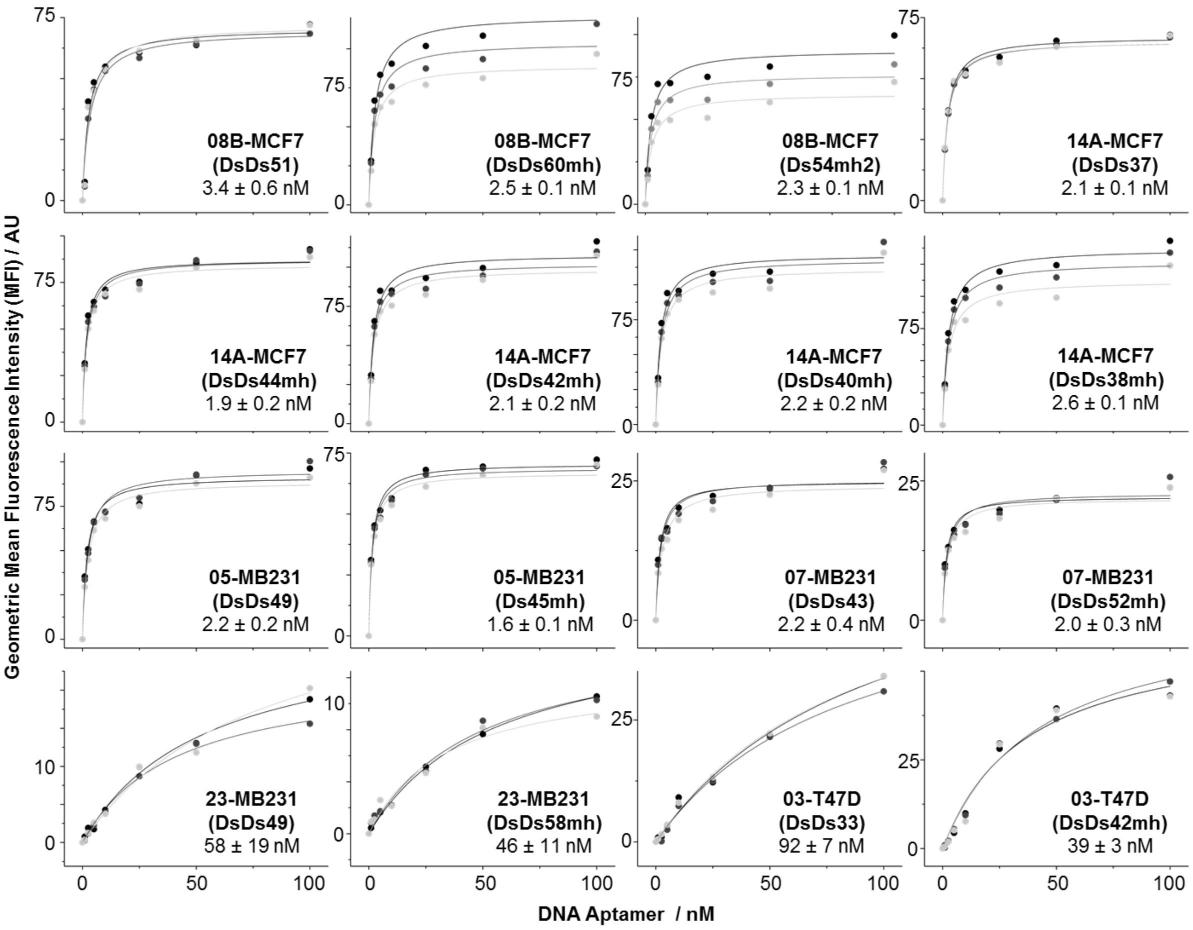

Figure S15: Binding of Ds-DNA aptamers and their variants to target cell lines. The target cells were incubated with the Alexa-488 labeled Ds-DNA aptamer and its variants (1 to 100 nM, the sequences are shown in Figures S8–S13) for 30 min at 4ºC, followed by flow cytometry analyses. The geometric mean fluorescence intensities were plotted against the aptamer concentrations, and the K_D values (average of three experiments) obtained from the curve fitting are shown with the standard deviations.

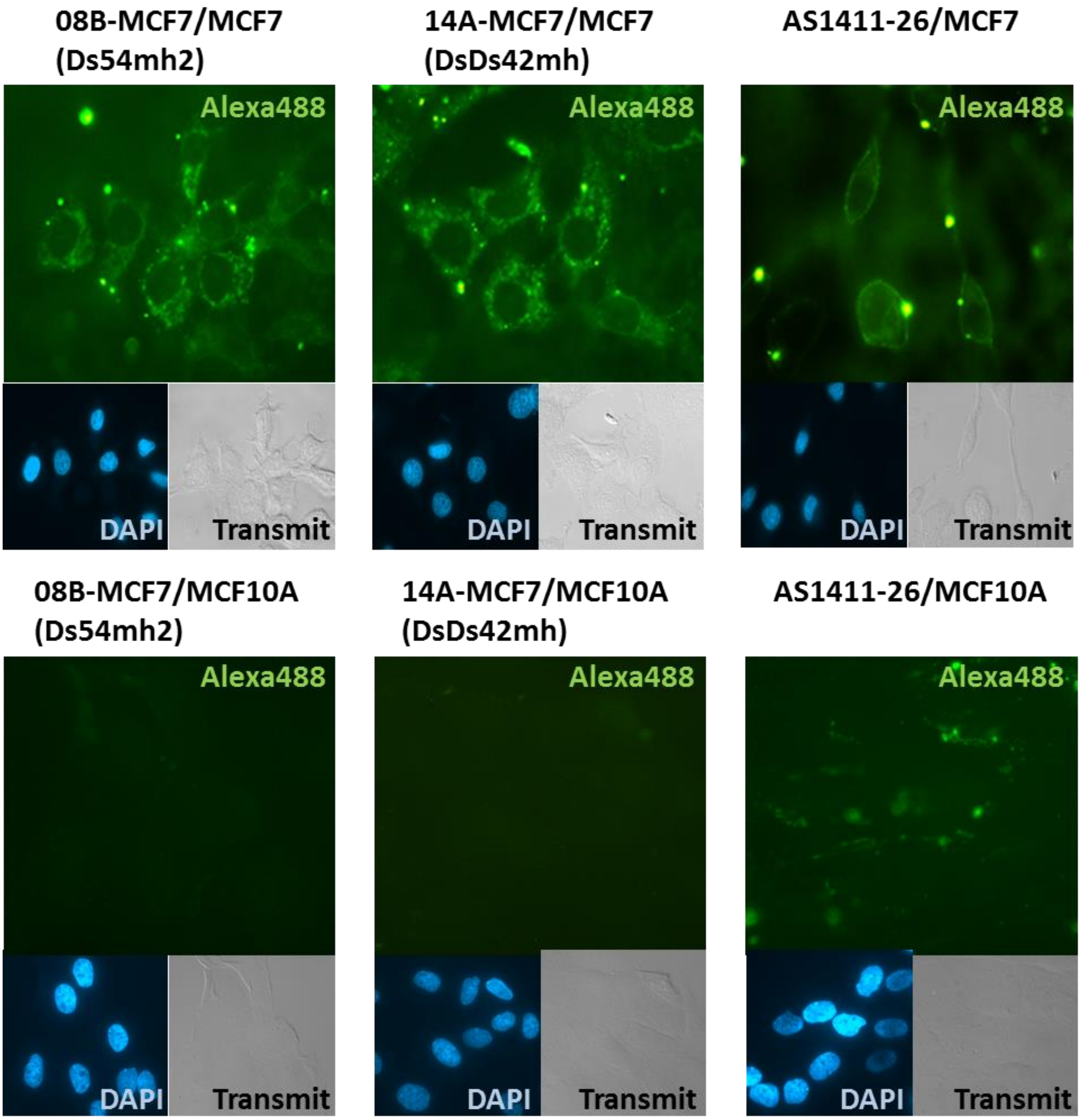

Figure S16: Ds-DNA aptamers 08B-MCF7(Ds54mh2) and 14A-MCF7(DsDs42mh) bound to MCF7 cells, but not to MCF10A cells. MCF7 and MCF10A cells were incubated with 250 nM of Alexa-488 labeled 08B-MCF7(Ds54mh2), 14A-MCF7(DsDs42mh), and AS1411-26 for 30 min at 4ºC, and analyzed by fluorescent microscopy (Alexa488, top; DAPI, lower left; bright-field, lower right).

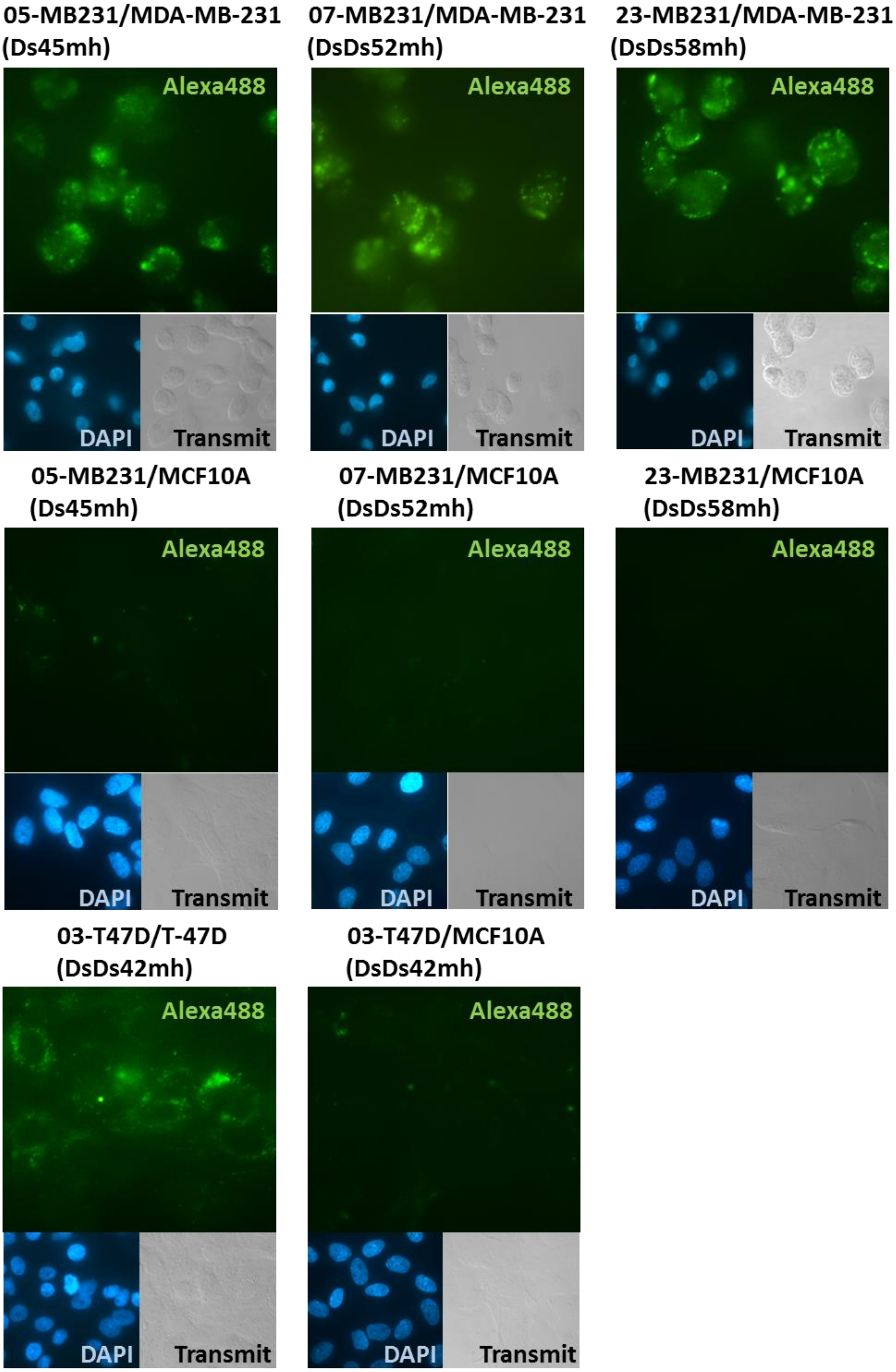

Figure S17: Ds-DNA aptamers 05-MB231(Ds45mh), 07-MB231(DsDs52mh), 23-MB231(DsDs58mh), and 03-T47D(DsDs42mh) bound to the target cells, but not to MCF10A cells. The target cells, MDA-MB-231 and T-47D, as well as MCF10A, were incubated with 250 nM of Alexa-488 labeled 05-MB231 (Ds45mh), 07-MB231 (DsDs52mh), 23-MB231 (DsDs58mh), and 03-T47D (DsDs42mh) for 30 min at 4ºC, and analyzed by fluorescent microscopy (Alexa488, top; DAPI, lower left; bright-field, lower right).

A

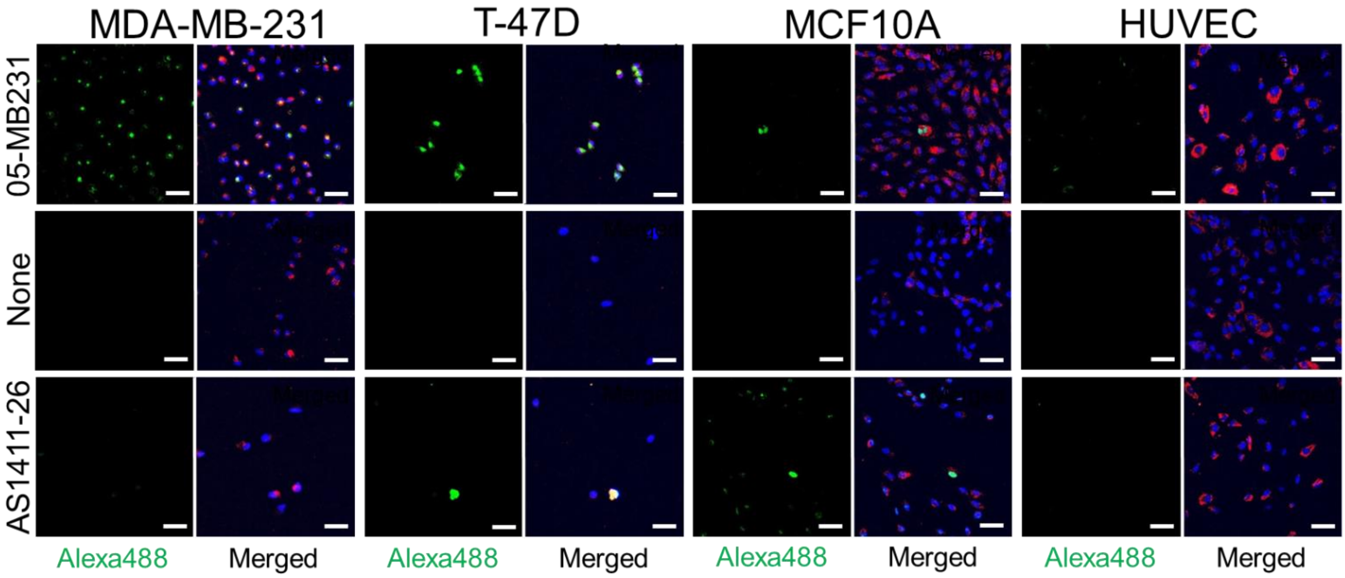

B

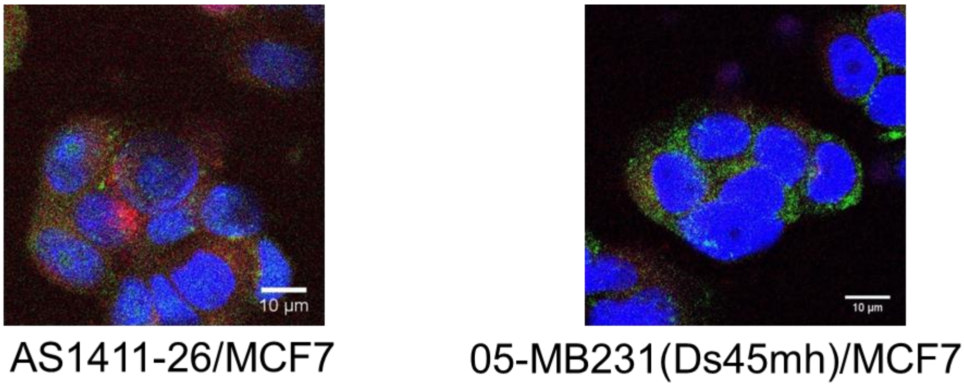

Figure S18: Confocal microscopy imaging. Ds-DNA aptamer 05-MB231(Ds45mh) bound to MDA-MB-231 and T-47D cells more tightly than AS1411 (A), and 05-MB231(Ds45mh) was internalized within the cancer cells (A, B). The cells were incubated in the presence or absence of 250 nM Alexa488-labeled 05-MB231 (Ds45mh) or AS1411-26 for 30 min at 37°C, after the incubation with LysoTracker (for 45 min) to detect acidic organelles inside the cell. The cells were then stained with DAPI and observed under 400 × magnification (A) or under 600 × magnification (B). In the merged images, the DAPI (nuclei, blue), Alexa488 (aptamer, green), and LysoTracker (red) signal images are overlaid. Scale bars: 50 µm (A) and 10 µm (B).

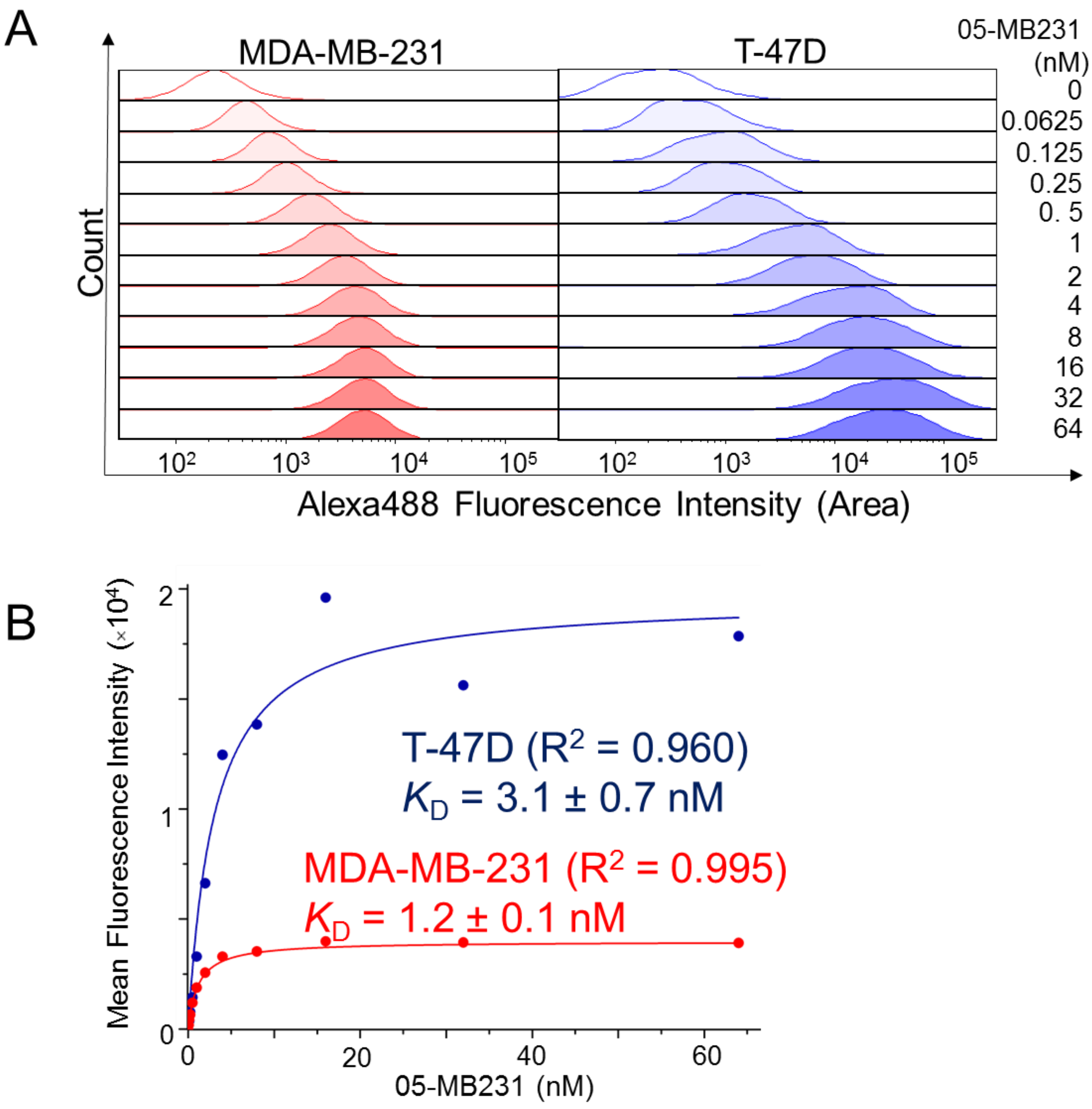

Figure S19: 05-MB231(Ds45mh) binding to MDA-MB-231 and T-47D cells. (A) Binding of 05-MB231(Ds45mh) to MDA-MB-231 (red) and T-47D (blue) cells was analyzed by flow cytometry. 05-MB231(Ds45mh) exhibited low binding affinity at low concentrations (0.0625 nM). (B) Binding curves of 05-MB231(Ds45mh) to MDA-MB-231 (red) and T-47D (blue) cells, showing that 05-MB231(Ds45mh) exhibits similar high binding affinities (K_D = 1–3 nM) to both MDA-MB-231 and T-47D cells.

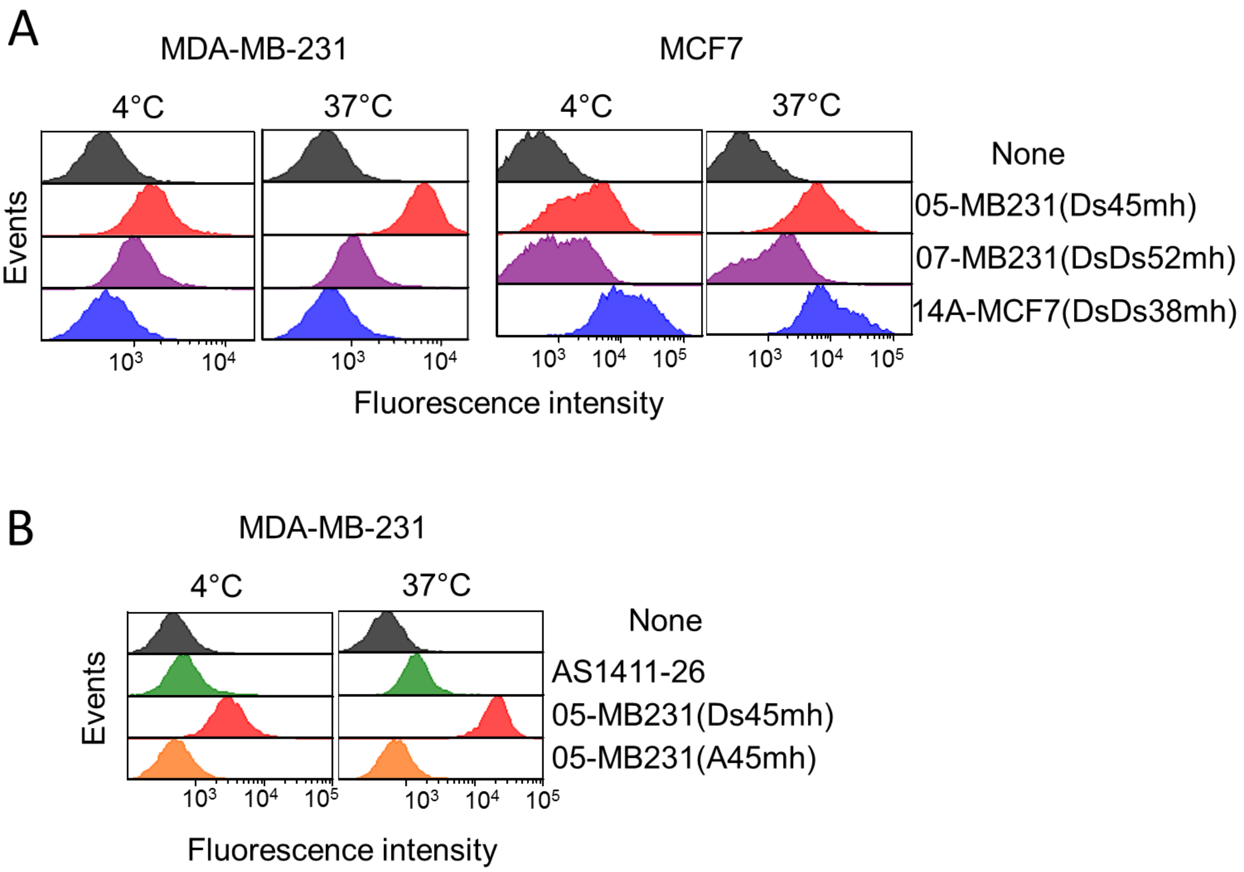

Figure S20: Ds-DNA aptamer binding analysis by flow cytometry. (A) Binding of FAM-labeled Ds-DNA aptamers, 05-MB231(Ds45mh), 07-MB231(DsDs52mh), and 14A-MCF7(DsDs38mh) to MDA-MB-231 and MCF7 cells was analyzed by flow cytometry, after a 30-min incubation at either 4°C or 37°C. 05-MB231(Ds45mh) exhibited higher binding to MDA-MB-231 at 37°C, than at 4°C. (B) Binding of Alexa488-labeled AS1411-26 and 05-MB231(Ds45mh) aptamers to MDA-MB-231 at either 4°C or 37°C. The binding efficiencies increased at 37°C, especially for 05-MB231, as compared with those at 4°C. The Ds-to-A variant (A45mh) did not bind to the cells.

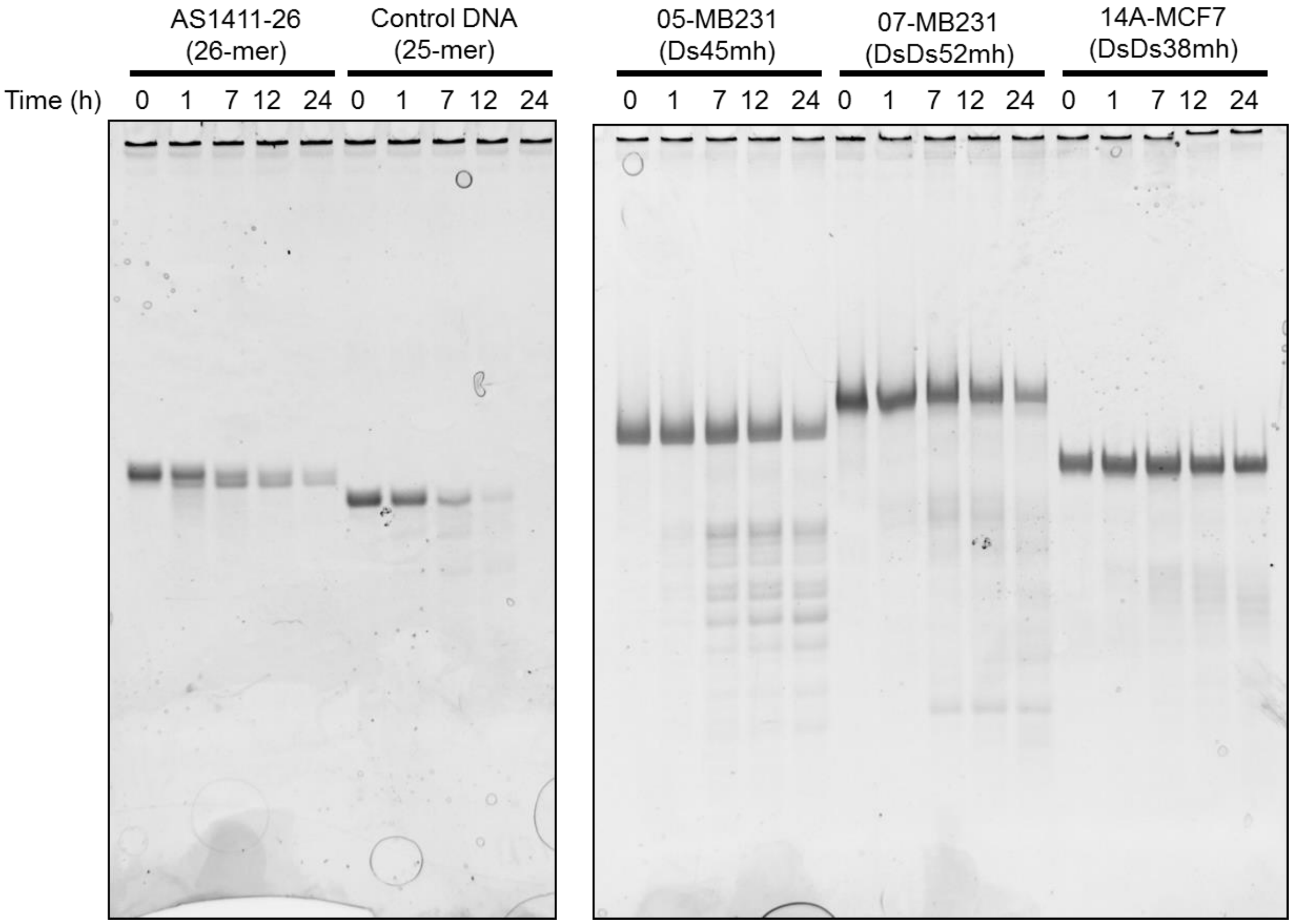

Figure S21: Stability analysis of Ds-DNA aptamers in human serum. Each DNA (250 pmol, 5 µl in 1× D-PBS) was mixed with 120 µl of human serum and incubated at 37°C. After the indicated incubation time, aliquots (10 µl) were obtained from the solutions, immediately mixed with 120 µl of denaturing solution (1× TBE with 10 M urea), and then frozen until PAGE analysis. The prepared samples were denatured at 75°C for 3 min and then a portion (5 µl) was subjected to 15% denaturing PAGE. The DNA on the gels was stained with SYBR Gold and detected with a bio-imaging analyzer (Fuji Film LAS-4000). The sequence of the Control DNA (25-mer) is 5′-CGACGGCGAACCTCCAAGCGGCAGC-3′.

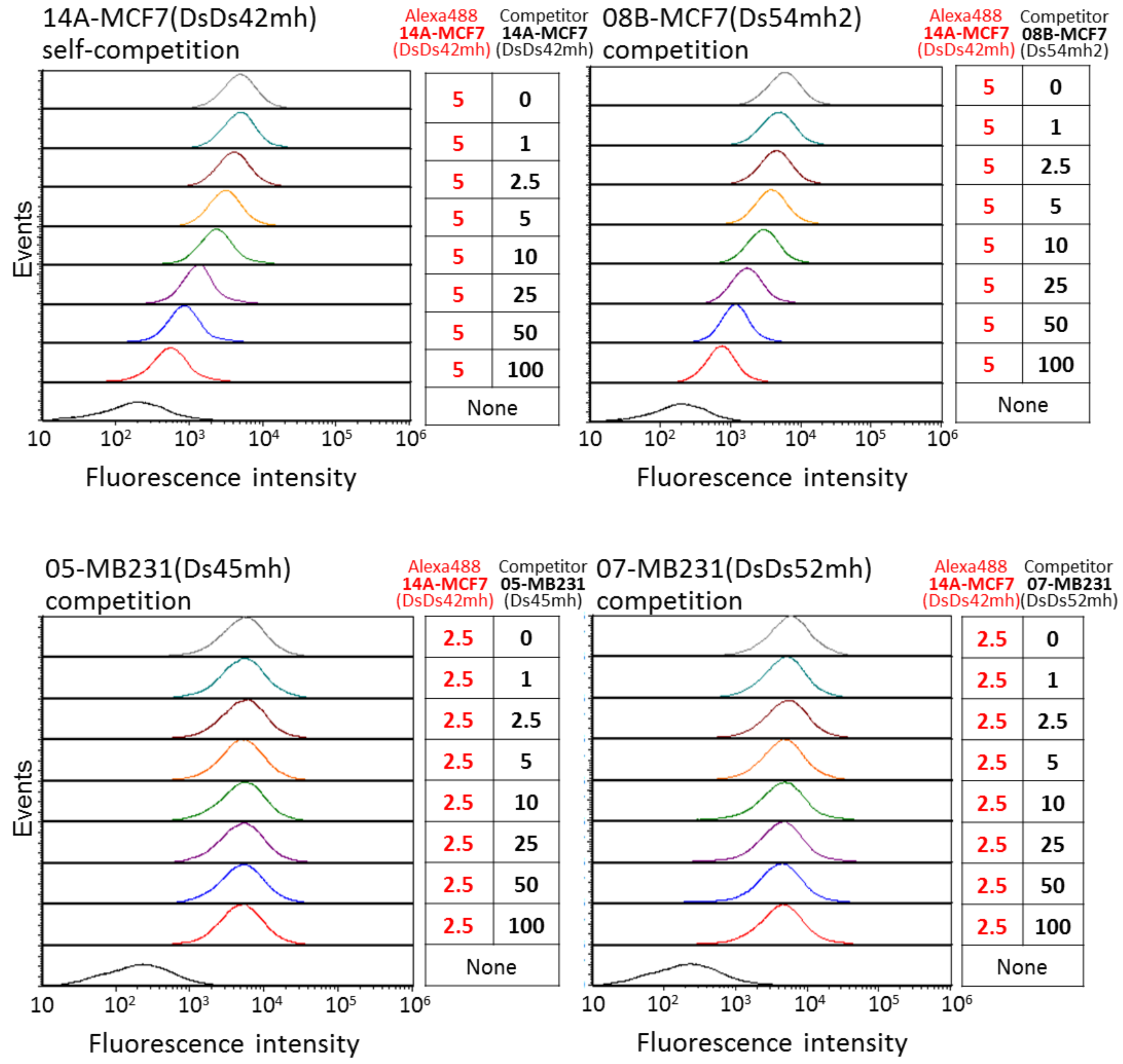

Figure S22: Competitive binding assays. Alexa488-labeled 14A-MCF7(DsDs42mh) (5 or 2.5 nM) was incubated with MCF7 cells, in the presence of non-labeled Ds-DNA aptamer (1 to 100 nM) at 4°C for 30 min, and analyzed by flow cytometry to quantify the Alexa488-labeled 14A-MCF7(DsDs42mh) bound to MCF7 cells.

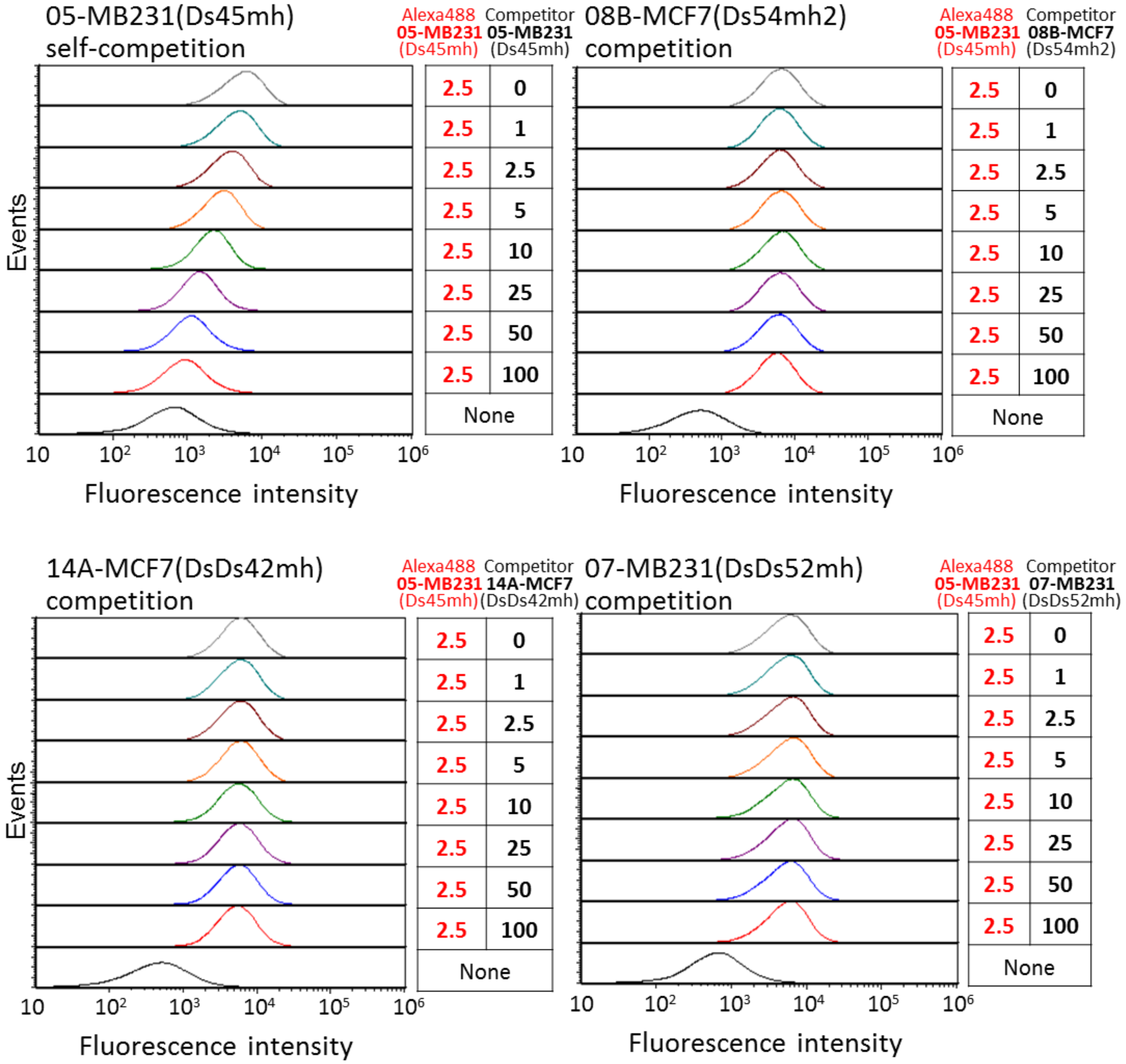

Figure S23: Competitive binding assays. Alexa488-labeled 05-MB231(Ds45mh) (2.5 nM) was incubated with MDA-MB-231 cells, in the presence of non-labeled Ds-DNA aptamer (1 to 100 nM) at 4°C for 30 min, and analyzed by flow cytometry to quantify the Alexa488-labeled 05-MB231(Ds45mh) bound to MDA-MB-231 cells.

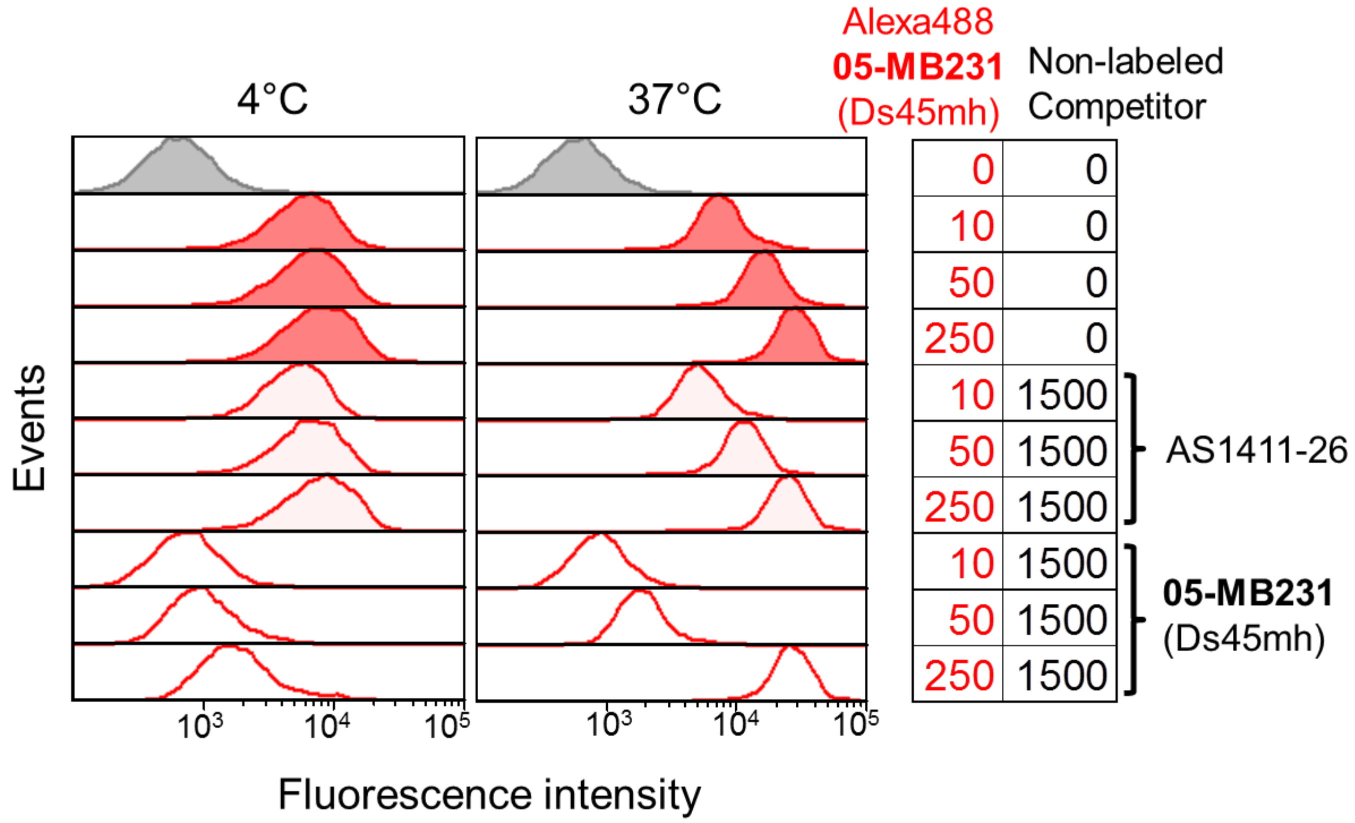

Figure S24: Competitive binding assays. Alexa488-labeled 05-MB231(Ds45mh) (10, 50, or 250 nM) was incubated with MDA-MB-231 cells, in the presence of 1,500 nM non-labeled AS1411-26 or 05-MB231(Ds45mh) at either 4°C or 37°C for 30 min, and analyzed by flow cytometry to quantify the Alexa488-labeled 05-MB231(Ds45mh) bound to MDA-MB-231 cells.

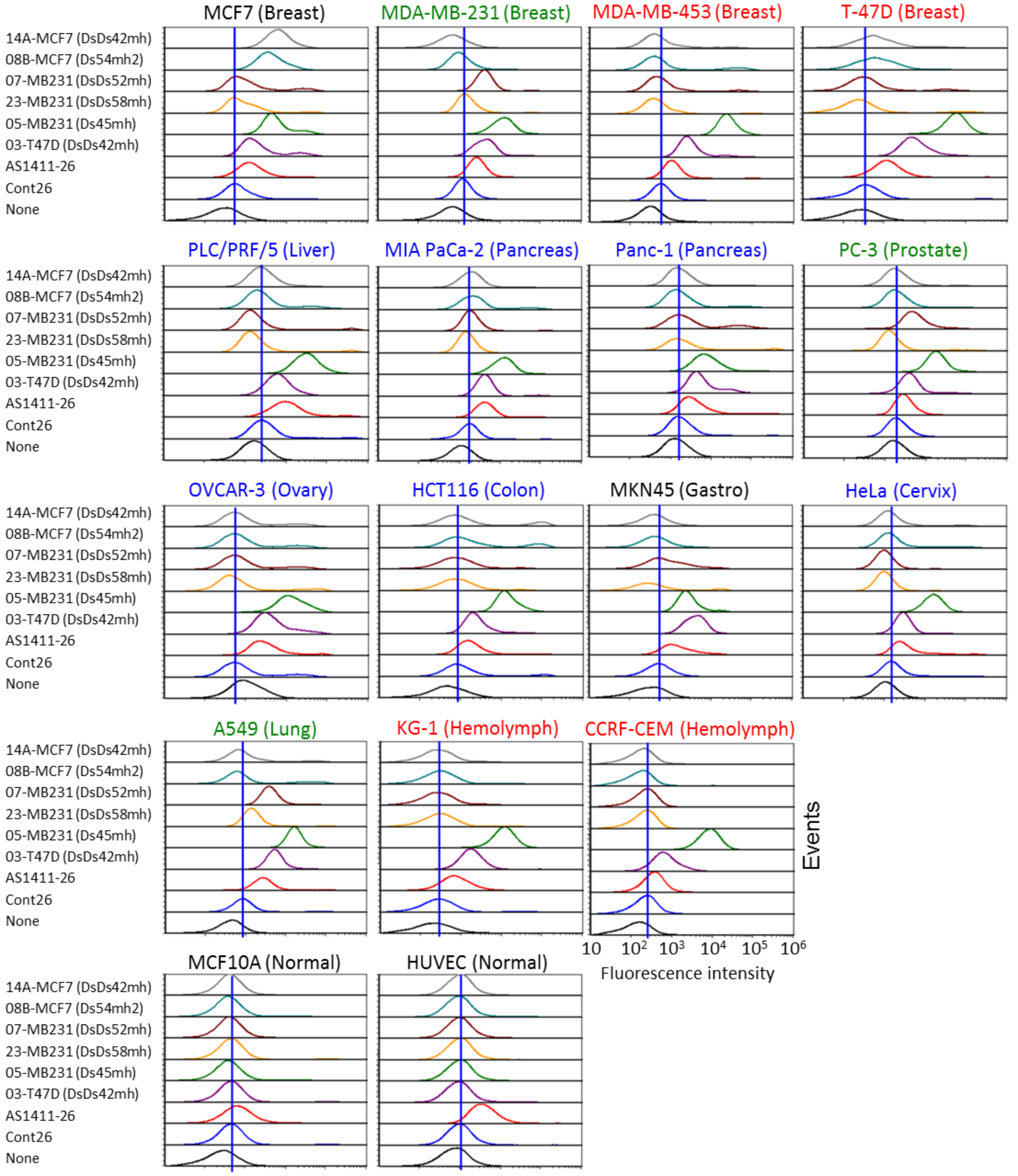

Figure S25: Binding of each Ds-DNA aptamer and AS1411-26 to various cell lines. Fifteen cancer cell lines and two normal cell lines were incubated with 250 nM of each Alexa488-labeled Ds-DNA aptamer, as well as AS1411-26 and Cont26 as controls, at 4°C for 30 min, and analyzed by flow cytometry. Based on the patterns of the fluorescent signals, the cancer cell lines were classified into four categories (the cell lines indicated with green, red, blue, and black). The blue straight line in each panel corresponds to the median fluorescence intensity for Cont26.

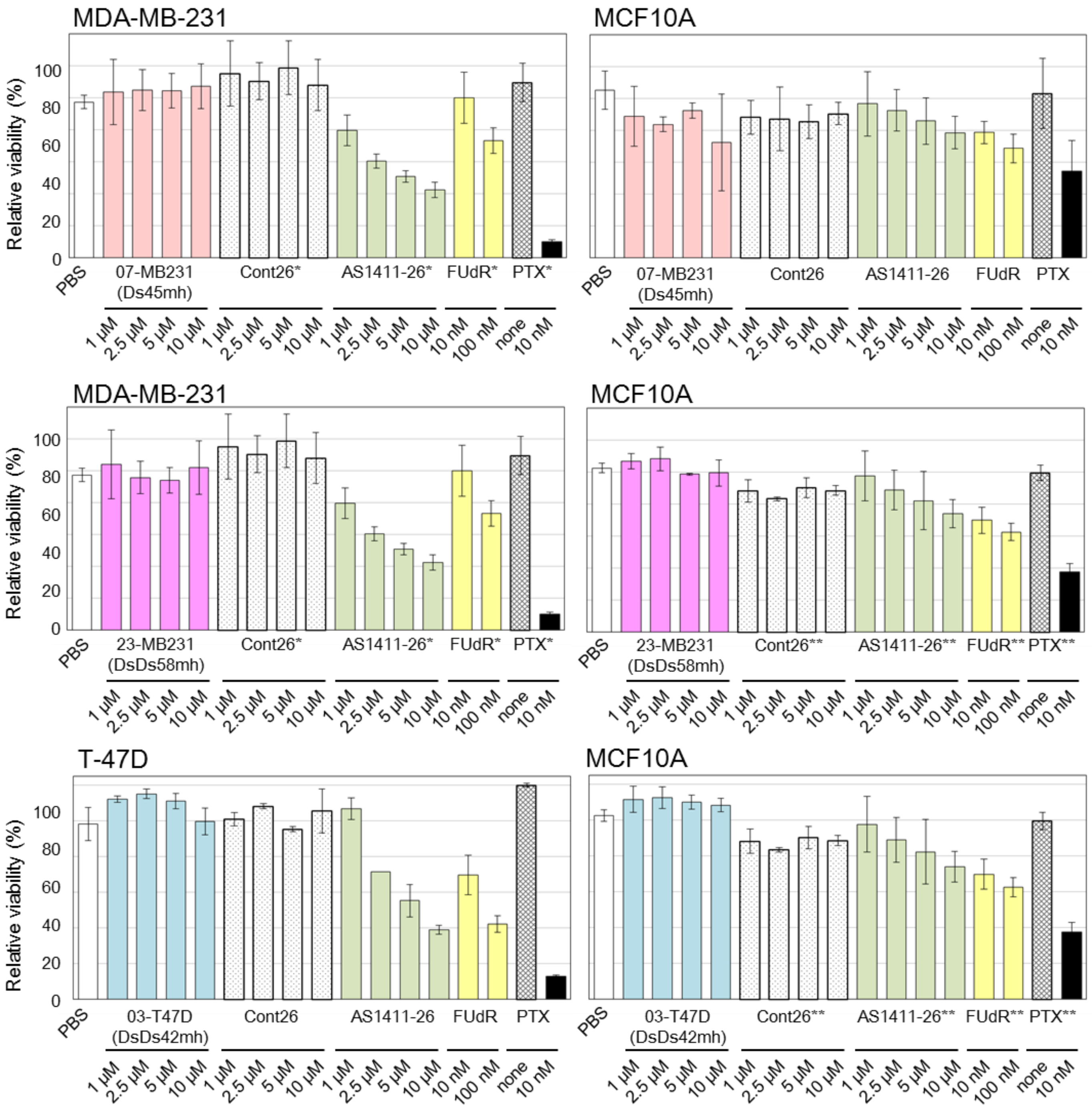

Figure S26: The Ds-DNA aptamers 07-MB231(DsDs52mh), 23-MB231(DsDs58mh), and 03-T47D(DsDs42mh) did not inhibit cell proliferation. The target cells, MDA-MB-231 and T-47D, as well as MCF10A as a control, were cultured in the presence of 07-MB231(DsDs52mh), 23-MB231(DsDs58mh), or 03-T47D(DsDs42mh) for 96 hours, followed by a WST-8 assay to determine relative cell viability. The assays were performed independently twice, and then the relative viabilities were averaged. The error bars represent standard deviations. *, **: The same data were used in the graphs for easy comparison, since the assays using 07-MB231(DsDs52mh) and 23-MB231(DsDs58mh) against MDA-MB-231 cells and those using 23-MB231(DsDs58mh) and 03-T47D(DsDs42mh) were performed within the same 96-well plates. FUdR: 5-fluorodeoxyuridine, PTX: Paclitaxel.

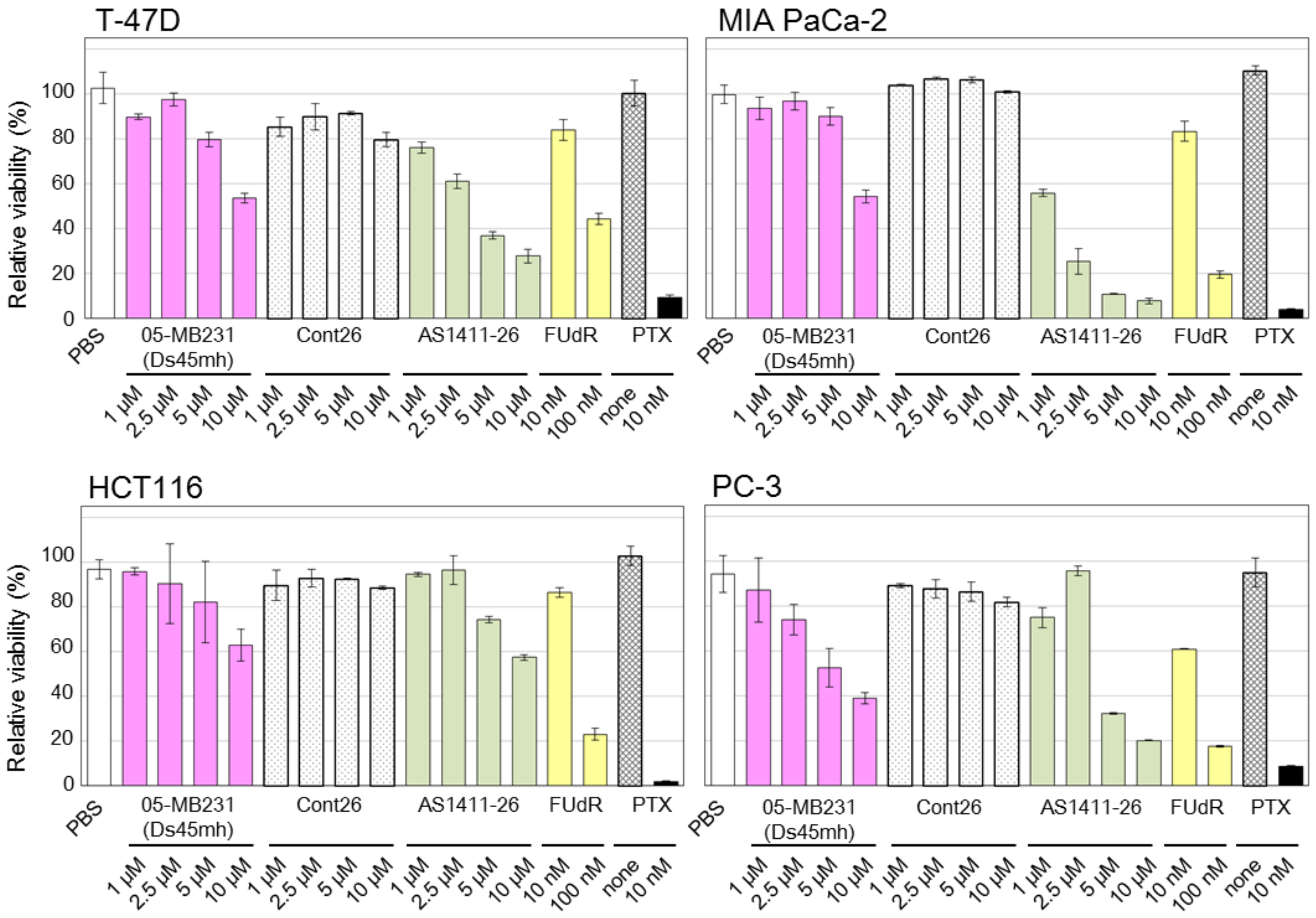

Figure S27: The Ds-DNA aptamer 05-MB231(Ds45mh) inhibited cancer cell proliferation. Several cancer cell lines were cultured in the presence of 05-MB231(Ds45mh) for 96 hours, followed by a WST-8 assay to determine relative cell viability. The assays were performed independently twice, and then the relative viabilities were averaged. The error bars represent standard deviations. FUdR: 5-fluorodeoxyuridine, PTX: Paclitaxel.

Table S1: DNA sub-libraries and PCR primers used in cell-ExSELEX. In each sub-library, barcode sequences (three bases) and random regions containing two Ds bases (red) are extracted and shown (N = a mixture of A, G, C, and T). A primer set, 5′-Rev019 and 3′T15-L-Fow026, was used in PCR during cell-ExSELEX for the Ds-strand separation. Another primer set, 5′-Rev019 and 3′-Fow026, was used for sequencing sample preparation.

Name	Sequence
N42Ds-01	5'-GAGNNNNNNNNNNNNNNNNNNNNNDsNNNNNNDsNNNNNNNNNNNNN-3'
N42Ds-02	5'-GACNNNNNNNNNNNNNNNNNNNNDsNNNNNNNDsNNNNNNNNNNNNN-3'
N42Ds-03	5'-CAGNNNNNNNNNNNNNNNNNNNDsNNNNNNNNDsNNNNNNNNNNNNN-3'
N42Ds-04	5'-CACNNNNNNNNNNNNNNNNNNDsNNNNNNNNNDsNNNNNNNNNNNNN-3'
N42Ds-05	5'-AGGNNNNNNNNNNNNNNNNNDsNNNNNNNNNNDsNNNNNNNNNNNNN-3'
N42Ds-06	5'-GGANNNNNNNNNNNNNNNNDsNNNNNNNNNNNDsNNNNNNNNNNNNN-3'
N42Ds-07	5'-GGGNNNNNNNNNNNNNNNDsNNNNNNNNNNNNDsNNNNNNNNNNNNN-3'
N42Ds-08	5'-GGCNNNNNNNNNNNNNNDsNNNNNNNNNNNNNDsNNNNNNNNNNNNN-3'
N42Ds-09	5'-GGTNNNNNNNNNNNNNDsNNNNNNNNNNNNNNDsNNNNNNNNNNNNN-3'
N42Ds-10	5'-CGANNNNNNNNNNNNNDsNNNNNNDsNNNNNNNNNNNNNNNNNNNNN-3'
N42Ds-11	5'-CGGNNNNNNNNNNNNNDsNNNNNNNDsNNNNNNNNNNNNNNNNNNNN-3'
N42Ds-12	5'-CGCNNNNNNNNNNNNNDsNNNNNNNNDsNNNNNNNNNNNNNNNNNNN-3'
N42Ds-13	5'-CGTNNNNNNNNNNNNNDsNNNNNNNNNDsNNNNNNNNNNNNNNNNNN-3'
N42Ds-14	5'-TGGNNNNNNNNNNNNNDsNNNNNNNNNNDsNNNNNNNNNNNNNNNNN-3'
N42Ds-15	5'-TGCNNNNNNNNNNNNNDsNNNNNNNNNNNDsNNNNNNNNNNNNNNNN-3'
N42Ds-16	5'-ACGNNNNNNNNNNNNNDsNNNNNNNNNNNNDsNNNNNNNNNNNNNNN-3'
N42Ds-17	5'-GCANNNNNNNNNNNNNDsNNNNNNNNNNNNNDsNNNNNNNNNNNNNN-3'
N42Ds-18	5'-GCGNNNNNNNNNNNNNNNNNDsNNNNNNDsNNNNNNNNNNNNNNNNN-3'
N42Ds-19	5'-GCCNNNNNNNNNNNNNNNNDsNNNNNNNDsNNNNNNNNNNNNNNNNN-3'
N42Ds-20	5'-GCTNNNNNNNNNNNNNNNNDsNNNNNNNNDsNNNNNNNNNNNNNNNN-3'
N42Ds-21	5'-CCANNNNNNNNNNNNNNNDsNNNNNNNNNDsNNNNNNNNNNNNNNNN-3'
N42Ds-22	5'-CCGNNNNNNNNNNNNNNNDsNNNNNNNNNNDsNNNNNNNNNNNNNNN-3'
N42Ds-23	5'-CCCNNNNNNNNNNNNNDsNNNNNNNNNNNNDsNNNNNNNNNNNNNNN-3'
N42Ds-24	5'-CCTNNNNNNNNNNNNNNDsNNNNNNNNNNNNDsNNNNNNNNNNNNNN-3'
5'-Rev019	5'-ACGACCGTTCTCTAATTTTGACGTT-3'
3'T15-L-Fow026	5'-TTTTTTTTTTTTTTT-C12spacer-ACCAAATTATTGCGATACAGACCCT-3'
3'-Fow026	5'-ACCAAATTATTGCGATACAGACCCT-3'

Table S2: Conditions of cell-ExSELEX targeting MCF7 cells. Conditions for the initial selection and the doped selections for each isolated aptamer (14A or 08B) are summarized. Pre- or post-counter selections were performed using MCF10A cells.

Library	Round	DNA (nM)	Volume (ml)	Cell	Binding	Time (min)	Wash	PCR cycles
N42Ds	1	1000	2	MCF7 (100 mm dish ×1)	4°C	60	5 ml × 5 times	15
	pre-2	50	2	MCF10A (100 mm dish ×1)	4°C	60		
	2	50	2	MCF7 (100 mm dish ×1)	4°C	30	5 ml / 5 min × 5 times	22
	pre-3	5	2	MCF10A (100 mm dish ×5)	4°C	60		
	3	5	10	MCF7 (100 mm dish ×1)	4°C	30	5 ml / 10 min × 5 times	20
	pre-4	2.5	2	MCF10A (100 mm dish ×5)	r.t.	60		
	4	2.5	10	MCF7 (100 mm dish ×1)	4°C	30	7 ml / 10 min × 5 times	21
	pre-5	1	2	MCF10A (100 mm dish ×5)	r.t.	60		
	5	1	10	MCF7 (100 mm dish ×1)	4°C	30	10 ml / 10 min × 5 times	20
	pre-6	1	2	MCF10A (100 mm dish ×5)	r.t.	60		
	6	1	10	MCF7 (100 mm dish ×1)	4°C	30	10 ml / 10 min × 5 times	18
	post-6		1	MCF10A (100 mm dish ×1)	r.t.	60		
	pre-7	1	2	MCF10A (100 mm dish ×5)	37°C	60		
	7	1	10	MCF7 (100 mm dish ×1)	4°C	30	10 ml / 10 min × 5 times	18
	post-7		1	MCF10A (100 mm dish ×1)	37°C	60		
Doped-14A-MCF7	1	50	10	MCF7 (100 mm dish ×1)	4°C	60	5 ml × 3 times	22
	2	5	10	MCF7 (100 mm dish ×1)	4°C	45	5 ml / 5 min × 3 times	25
	3	2	5	MCF7 (100 mm dish ×1)	4°C	30	5 ml / 5 min × 3 times	28
	4	1	5	MCF7 (100 mm dish ×1)	4°C	30	5 ml / 5 min x 5times	21
Doped-08B-MCF7	1	50	10	MCF7 (100 mm dish ×1)	4°C	60	5 ml × 3 times	21
	2	5	10	MCF7 (100 mm dish ×1)	4°C	45	5 ml / 5 min × 3 times	25
	3	2	5	MCF7 (100 mm dish ×1)	4°C	30	5 ml / 5 min × 3 times	25
	4	1	5	MCF7 (100 mm dish ×1)	4°C	30	5 ml / 5 min × 5 times	16

Table S3: Conditions of cell-ExSELEX targeting MDA-MB-231 cells. Conditions for the initial selection and the doped selections for each isolated aptamer (05, 07 or 23) are summarized. Pre-counter selections were performed using MCF10A cells.

Library	Round	DNA (nM)	Volume (ml)	Cell	Binding	Time (min)	Wash	PCR cycles
N42Ds	1	50	20	MDA-MB-231 (100 mm dish ×1)	4°C	60	5 ml × 5 times	14
	2	10	10	MDA-MB-231 (100 mm dish ×1)	4°C	60	5 ml / 5 min × 5 times	15
	pre-3	5	10	MCF10A (150 mm dish ×1)	4°C	60		
	3	5	10	MDA-MB-231 (100 mm dish ×1)	4°C	45	5 ml / 10 min × 5 times	21
	pre-4	2.5	10	MCF10A (150 mm dish ×1)	4°C	60		
	4	2.5	10	MDA-MB-231 (100 mm dish ×1)	4°C	30	7 ml / 10 min × 5 times	23
	pre-5	2.5	10	MCF10A (150 mm dish ×1)	4°C	60		
	5	2.5	10	MDA-MB-231 (100 mm dish ×1)	4°C	30	10 ml / 10 min × 5 times	21
	pre-6	1	10	MCF10A (150 mm dish ×1)	4°C	60		
	6	1	10	MDA-MB-231 (100 mm dish ×1)	4°C	30	10 ml / 10 min × 5 times	24
	pre-7	1	5	MCF10A (150 mm dish ×1)	4°C	60		
	7	1	5	MDA-MB-231 (100 mm dish ×1)	4°C	30	10 ml / 10 min × 5 times	19
Doped-05-MB231	1	50	10	MDA-MB-231 (100 mm dish ×1)	4°C	60	5 ml × 3 times	18
	2	5	10	MDA-MB-231 (100 mm dish ×1)	4°C	45	5 ml / 5 min × 3 times	21
	3	2	5	MDA-MB-231 (100 mm dish ×1)	4°C	30	5 ml / 5 min × 3 times	24
	4	1	5	MDA-MB-231 (100 mm dish ×1)	4°C	30	5 ml / 5 min × 5 times	21
Doped-07-MB231	1	50	10	MDA-MB-231 (100 mm dish ×1)	4°C	60	5ml × 3 times	15
	2	5	10	MDA-MB-231 (100 mm dish ×1)	4°C	45	5 ml / 5 min × 3 times	24
	3	2	5	MDA-MB-231 (100 mm dish ×1)	4°C	30	5 ml / 5 min × 3 times	24
	4	1	5	MDA-MB-231 (100 mm dish ×1)	4°C	30	5 ml / 5 min × 5 times	21
Doped-23-MB231	1	50	10	MDA-MB-231 (100 mm dish ×1)	4°C	60	5ml × 3 times	15
	2	5	10	MDA-MB-231 (100 mm dish ×1)	4°C	45	5 ml / 5 min × 3 times	21
	3	2	5	MDA-MB-231 (100 mm dish ×1)	4°C	30	5 ml /5 min × 3 times	25
	4	1	5	MDA-MB-231 (100 mm dish ×1)	4°C	30	5 ml / 5 min × 5 times	23

Table S4: Conditions of cell-ExSELEX targeting T-47D cells. Conditions for the initial selection and the doped selection for the isolated aptamer (03) are summarized. Pre-counter selections were performed using MCF10A cells.

Library	Round	DNA (nM)	Volume (ml)	Cell	Binding	Time (min)	Wash	PCR cycles
N42Ds	1	50	20	T-47D (100 mm dish ×1)	4 ºC	60	5 ml x 5 times	23
	2	10	10	T-47D (100 mm dish ×1)	4 ºC	60	5 ml / 5 min × 5 times	24
	pre-3	5	10	MCF10A (150 mm dish ×1)	4 ºC	60		
	3	5	10	T-47D (100 mm dish ×1)	4 ºC	30	5 ml / 10 min × 5 times	25
	pre-4	2.5	10	MCF10A (150 mm dish ×1)	4 ºC	60		
	4	2.5	10	T47D (100 mm dish ×1)	4 ºC	30	7 ml / 10 min × 5 times	26
	pre-5	2.5	10	MCF10A (150 mm dish ×1)	4 ºC	60		
	5	2.5	10	T-47D (100 mm dish ×1)	4 ºC	30	10 ml / 10 min × 5 times	25
	pre-6	1	10	MCF10A (150 mm dish ×1)	4 ºC	60		
	6	1	10	T-47D (100 mm dish ×1)	4 ºC	30	10 ml / 10 min × 5 times	24
	pre-7	1	5	MCF10A (150 mm dish ×1)	4 ºC	60		
	7	1	5	T-47D (100 mm dish ×1)	4 ºC	30	10 ml /10 min × 5 times	22
Doped-03-T47D	1	50	10	T-47D (100 mm dish ×1)	4 ºC	60	5 ml × 3 times	19
	2	5	10	T-47D (100 mm dish ×1)	4 ºC	45	5 ml / 5 min × 3 times	21
	3	2	5	T-47D (100 mm dish ×1)	4 ºC	30	5 ml / 5 min × 3 times	20
	4	1	5	T-47D (100 mm dish ×1)	4 ºC	30	5 ml / 5 min × 5 times	19

Table S5: Sequences of the barcode and the random region of each clone selected after seven rounds of the initial selection targeting MCF7, MDA-MB-231, and T-47D cells. The Ds positions (red) in each clone were determined from the corresponding barcode sequences. Sequences of the top counts (more than 2% in the analyzed reads) in each cell-ExSELEX are listed.

**MCF7**			
**Total reads**	**246,903**		
**Extracted reads**	**80,592**		
**Analyzed reads (Counts ≥2)**	**21,600**		
**Selected clones**	**Counts**		**Barcode-N42 sequences**
14A-MCF7	9,735	45.1%	tggCCACTCTGTCGTCDsGTCGAACTGCDsATGAGCGTGCTTTGGAG
	1,661	7.7%	cgtCTGGTTGGGATACDsTAGCGCATGDsACCGTTCTCGAAACCCTT
	1,282	5.9%	ccaCCAAAGAGCGCTATTDsTCGTGCATGDsACCATCCTGGACTCTT
	734	3.4%	ggtATCCCCTGGCACADsTATAGACAGGCGTGDsCTATTGGCGCGCA
	684	3.2%	ggcGCGCCCTTTTCGTTDsTTGGTTGGTGGTGDsGGAGAAAAGATGG
	669	3.1%	ggtAGCGGTGTATAGCDsTATGGACTGCAGAGDsCCTTCGCTGACTA
	551	2.6%	agaCGCTACAATAAATDsTTGATCTAACCTDsTATCCTTGGGCATGC
08B-MCF7	484	2.2%	ggcCCGGCTGGCATGTADsTCATGCCTCCTGGDsCTAAGGTTTCTAA
**Target: MDA-MB-231**			
**Total reads**	**497,341**		
**Extracted reads**	**128,607**		
**Analyzed reads (Counts ≥2)**	**95,082**		
**Selected clones**	**Counts**		**Barcode-N42 sequences**
23-MB231	47,092	49.5%	cccTCTTAAGTTTATAGDsTGTCTTTTGGTDsCCTAGGGCGTTTTAA
05-MB231	22,521	23.7%	aggGGTGTTTGTGCCGTGAGDsTGTAGCGTTTDsGAATTTAGGCTTT
07-MB231	18,234	19.2%	gggCCTAGGCAGCTTGGGDsTATCTTTCAGTCDsATTTCAGTCGGCT
	3,001	3.2%	gctTCTGTTTTTCTTTACADsCTTTGCCGDsGGAGGTCTGATTTCTC
**Target: T-47D**			
**Total reads**	**638,160**		
**Extracted reads**	**205,801**		
**Analyzed reads (Counts ≥2)**	**192,728**		
**Selected clones**	**Counts**		**Barcode-N42 sequences**
03-T47D	156,326	81.1%	cagAGTAAGAAACGAAGATCACDsACTAGGGGDsCCTCGAAGTCTAC
	16,436	8.5%	cagGCCAAAGAGCCTAACGCACDsACTAGGGGDsGTGAGCTCTACCC
	4,745	2.5%	cgtTTTTATTAAAGCCDsATTGCATGTDsGACAGACATCCTGTCAAC
